# Risk factors for carbapenem-resistant Klebsiella pneumoniae infection in hospitalized patients: a meta-analysis

**DOI:** 10.3389/fcimb.2026.1717419

**Published:** 2026-03-10

**Authors:** Chengyang Jin, Xuejiao Xiang, Qun Zhang

**Affiliations:** Department of Disease Prevention and Control, Second Affiliated Hospital of Navy Medical University, Shanghai, China

**Keywords:** carbapenem-resistance, infections, Klebsiella pneumoniae, meta-analysis, risk factor

## Abstract

**Background:**

Healthcare-associated infections due to carbapenem-resistant Klebsiella pneumoniae (CRKP) are a global public health threat with rising hospital morbidity and mortality. We conducted a meta-analysis to systematically identify CRKP infection risk factors.

**Methods:**

We searched Medline, Embase, Web of Science, and Cochrane Library for studies published January 1991–December 2024. Pooled odds ratio (OR)/95% confidence intervals (CIs) were used to assess risk factors; publication bias was evaluated via funnel plots and Egger’s test, and robustness via leave-one-out sensitivity analysis.

**Results:**

Fifty-one studies (13,860 patients: 4,711 CRKP cases, 9,149 carbapenem-susceptible K. pneumoniae controls) were included, with 43 reported risk factors. Thirty-one were significant: demographic/underlying diseases [male sex (OR = 1.31), kidney diseases (OR = 1.47), respiratory system diseases (OR = 2.69), cardiovascular diseases (OR = 1.34)]; invasive procedures [endoscopy (OR = 4.08), tracheal cannula (OR = 3.72), mechanical ventilation (OR = 3.61)]; medical environment [ICU admission (OR = 4.27), pre-infection hospital stay (mean difference=14.98 days)]; antibiotics [tigecycline (OR = 5.97), carbapenems (OR = 4.79), which may reflect disease severity, prior colonization]. Subgroup analysis showed regional heterogeneity: Western populations had higher risks with cephalosporins (OR = 2.68 vs. Eastern 1.55) and fluoroquinolones (OR = 3.58 vs. Eastern 1.89), while Eastern populations had higher risks with invasive procedures (dialysis: OR = 4.47 vs. Western 2.03). Sensitivity analysis confirmed robust results.

**Conclusions:**

This meta-analysis reports endoscopy and surgical drainage as distinct subtypes of invasive procedural factors associated with hospital-acquired CRKP infection and describes regional differences in associated factors between Eastern and Western populations. These findings, based on observational evidence, provide preliminary insights for targeted prevention strategies.

**Systematic review registration:**

https://www.crd.york.ac.uk/PROSPERO, identifier CRD42024628428.

## 1 Introduction

Carbapenem-resistant Klebsiella pneumoniae is a highly virulent pathogen that has become increasingly prevalent in healthcare settings due to the widespread use of carbapenem antibiotics (Ernst et al., 2020). Globally, CRKP accounts for 28.69% of Klebsiella pneumoniae (KP) infections, with significant regional disparities. High-income regions in North America report the lowest prevalence at 14.29%, whereas Greece has the highest at 70.61%, closely followed by India at 67.62% (Lin et al., 2024). Alarmingly, CRKP harbors multiple resistance genes, such as *bla*_KPC_ (Zhang et al., 2020; Jin et al., 2021; Tian et al., 2022), and NDM, conferring high-level resistance to carbapenems and other antibiotics. This multidrug resistance severely restricts treatment options, resulting in a mortality rate exceeding 40% (Yao et al., 2024), and posing a grave threat to human health. Since 2018, the World Health Organization (WHO) has ranked CRKP as the top-priority pathogen on its global list of antimicrobial resistance concerns (Antochevis et al., 2025).

Identifying risk factors for CRKP infections is pivotal for preventing and controlling hospital-acquired cases. Previous meta-analyses have identified several well-established risk factors, including prior antibiotic use and comorbidities (Liu et al., 2018; Li et al., 2020; Zhu et al., 2020; Zhu et al., 2023). However, recent studies have proposed additional potential risk factors, such as endoscopic procedures and surgical drainage (Zhang et al., 2021a; Cao et al., 2022; Chen et al., 2022; Huang et al., 2023). These factors remain underrepresented in existing meta-analyses, and their association with hospital-acquired CRKP infections remains controversial. Moreover, the geographical variability of risk factors contributes to inconsistent findings across studies. Notably, previous meta-analyses have neither explored region-specific risk factors nor proposed targeted preventive strategies. Therefore, well-designed studies are urgently needed to elucidate the risk factors for hospital-acquired CRKP infections.

Against this backdrop, we conducted a meta-analysis to identify the key factors associated with hospital-acquired CRKP infections, aiming to provide region-specific insights for effective prevention and control.

## 2 Methods

This meta-analysis was conducted according to the Preferred Reporting Items for Systematic Reviews and Meta-Analyses (PRISMA) guidelines. This study was registered with PROSPERO, CRD42024628428.

### 2.1 Search strategy

Two authors searched for relevant studies in Medline (through Pubmed), Embase (through Ovid), Web of Science and Cochrane Library databases that were published from January 1991 to December 2024. The primary search terms included (“klebsiella pneumoniae”[All Fields] OR “klebsiella pneumoniae” [MeSH Terms] OR “klebsiella”[All Fields] OR “klebsiella”[MeSH Terms]) AND (risk factors[MeSH Terms] OR risk factor[tiab] OR risk factors[tiab] OR predict*[tiab]), The exact search query for each database is highlighted in the **Supplementary Material**. We included case-control studies (including case-control, case-case-control, and nested case-control study) and cohort studies investigating the association between risk factors assessed at baseline and CRKP. Only studies published in English were considered. Reference lists in selected articles and relevant review articles were manually searched to identify additional studies.

### 2.2 Inclusion and exclusion criteria

Studies were enrolled if they met the following criteria: (1) they compared the risk factors for CRKP infections with those for Carbapenem-susceptible Klebsiella pneumoniae (CSKP) infections; (2) the studies were published in English; (3) infection, no infection, or colonization was explicitly defined, and antibiotic exposure was reported; (4) *K. pneumoniae* was isolated and identified, and a test for resistance to carbapenem (meropenem, imipenem, or ertapenem) with clear microbiological methods was reported; (5) the study type was either a case−control or cohort study. Excluded from this analysis were reports, letters, reviews, cross-sectional studies, meeting abstracts lacking sufficient data, as well as studies involving subjects under 18 years of age. The risk factors for surgical drainage in this study are limited to drainage surgeries related to abdominal infections and endoscopic procedures specifically refer to Bronchoscopy. Antibiotic exposure refers to the use of antibiotics occurring ≥48 hours before CRKP diagnosis (or culture positivity), excluding antibiotics initiated after the onset of infection symptoms or CRKP identification.

### 2.3 Data extraction

Two independent authors extracted data from the eligible studies. The extracted data included the first author’s name, publication year, country, study type, period, sample size(case/control), and risk factors.

### 2.4 Risk of bias assessment

Two reviewers independently assessed the quality using the Newcastle-Ottawa quality assessment scale (NOS). The full score of the scale was 9, with 0−4 points for low-quality studies, 5−6 points for moderate-quality studies, and 7 −9 points for high-quality studies. Consensus with a third reviewer was used to resolve disagreements. Risk of publication bias was assessed by visual inspection of funnel plots for all risk factors reported and was tested by Egger regression test. The heterogeneity of those variations across studies was examined with I-squared (*I^2^*) values, which provide a percentage of the proportion.

### 2.5 Statistics

Pooled odds ratio (OR) for each risk factor was calculated using the general inverse variance method, with either a random-effects or fixed-effects model applied based on the degree of heterogeneity. The results were synthesized and presented in forest plots. Heterogeneity among studies was systematically investigated using the *I²* statistic. All statistical analyses were conducted using R 4.3.3 (R Foundation for Statistical Computing).

## 3 Results

### 3.1 Study identification

Through the literature search in PubMed, Embase, Web of Science, and the Cochrane Library, we identified 2240 references. Duplicate references were identified and removed (n=1814), resulting in 426 articles. After screening titles and abstracts and search updates, 112 articles were selected for full-text review, resulting in 51 studies included in this meta-analysis (**Supplementary Figure 1**).

### 3.2 Study characteristics

A list of the 51 studies included with their characteristics is presented in **Table 1**. Of these studies, nine were cohort studies in design. The remaining 42 studies were either case-control studies (n = 39) or case-case-control studies (n = 3). About one-half of the studies (n=26, 51%) recruited patients during the years 2015 and 2024, 25 studies started recruitment between 2000 and 2015. Median study duration was 2.9 years (interquartile range, 2.5-4.8 years). Most of the studies were single-center studies (n = 41, 82.4%), whereas only ten were multicentric in design. Overall, in total 13860 people were included in this study, including 4711 CRKP infections and 9149 CSKP infections. Twenty-one studies (41.2%) were rated to be of high quality.

**Table 1:** Characteristics of the studies included in the meta-analysis.

Author and Year	Study country	Study design	Start	End	During	Case/control	Quality score
Falagas, 2007 (Falagas et al., 2007)	Greece	case-control study	1/10/2000	1/5/2006	5 years 7 months	53/53	7
Patel, 2008 (Patel et al., 2008)	USA	matched case-control study	1/7/2004	30/6/2006	1 year 11 months	99/99	6
Hussein, 2009 (Hussein et al., 2013)	Israel	case-control study	1/1/2006	1/4/2007	1 year 3 months	88/373	8
Wu, 2011 (Wu et al., 2011)	China	matched case-control study	1/7/2006	1/7/2008	2 years	39/78	6
Kritsotakis, 2011 (Kritsotakis et al., 2011)	Greece	case-case-control study	1/2/2006	1/3/2008	2 years 1 month	96/151	7
Liu, SW 2012 (Liu et al., 2012)	China	matched case-control study	1/1/2007	1/12/2009	2 years 11 months	25/50	6
Correa, 2013 (Correa et al., 2013)	Brazil.	case-control study	1/1/2006	1/8/2008	2 years 7 months	20/40	6
Hussein, 2013 (Hussein et al., 2009)	Israel	case-control study	1/1/2006	31/12/2008	2 years 11 months	103/214	7
Shilo, 2013 (Shilo et al., 2013)	Israel	case-control study	1/1/2006	1/4/2009	3 years 3 months	135/127	7
Simkins, 2014 (Simkins et al., 2014)	USA	case-control study	1/1/2006	31/12/2010	4 years 11 months	13/39	6
Candevir, 2015 (Candevir et al., 2015)	Turkey	cohort study	1/1/2012	31/12/2012	1 year	47/51	6
Pereira, 2015 (Pereira et al., 2015)	USA	cohort study	1/1/2010	31/1/2013	3 years	20/36	6
Pouch, 2015 (Pouch et al., 2015)	USA	case–control study	1/1/2007	31/12/2010	3 years 11 months	20/80	6
Vardakas, 2015 (Vardakas et al., 2015)	Greece	cohort study	1/1/2006	1/10/2009	3 years 9 months	73/18	7
Hu, 2016 (Hu et al., 2016)	China	matched case-control study	1/1/2011	30/6/2013	2 years 5 months	65/65	6
Hoxha, 2016 (Hoxha et al., 2016)	Italy	cohort study	1/11/2012	1/7/2013	8 months	49/49	8
Zheng, X 2017 (Zheng et al., 2017)	China	case-control study	1/1/2013	1/12/2014	1 year 11 months	31/17	7
Zheng, B 2017 (Zheng et al., 2017)	China	case-control study	1/1/2013	1/7/2015	2 years 6 months	51/51	7
Wang, Z 2018 (Wang et al., 2018)	China	case-control study	1/1/2010	31/12/2014	4 years 11 months	48/48	6
Xiao, TT 2018 (Xiao et al., 2018)	China	case-control study	1/1/2013	1/12/2015	2 years 11 months	135/293	6
Zheng, SH, 2018 (Zheng et al., 2018)	China	case-control study	1/1/2014	1/12/2016	2 years 11 months	59/230	6
Cienfuegos, 2019 (Cienfuegos-Gallet et al., 2019)	USA	case-control study	1/2/2014	1/9/2015	1 year 8 months	49/289	6
Liu, JL2019 (Liu et al., 2019)	China	case-control study	1/1/2014	1/9/2018	4 years 8 months	20/69	6
Pan, 2019 (Pan et al., 2019)	China	matched case-control study	1/1/2014	1/12/2014	1 year	66/132	6
Chang, 2019 (Chang et al., 2020)	China	cohort study	1/1/2014	1/7/2018	4 years 6 months	46/239	6
Wu, D 2020 (Wu et al., 2020)	China	cohort study	1/12/2012	1/7/2019	6 years 7 months	30/38	6
Xiao, 2020 (Xiao et al., 2020)	China	cohort study	1/1/2013	1/12/2015	2 years 11 months	104/267	6
Gupta, 2020 (Gupta et al., 2021)	India	case-control study	1/8/2014	1/7/2015	11 months	85/26	7
Li, YY 2020 (Li et al., 2020)	China	case-control study	1/1/2014	1/6/2019	5 years 5 months	164/328	7
Hsu, 2021 (Hsu et al., 2021)	China	matched case-control study	1/10/2017	1/12/2019	2 years 2 months	36/72	6
Zhang, HJ 2021 (Zhang et al., 2021a)	China	case-control study	1/1/2016	1/12/2018	2 years 11 months	142/142	6
Zhang, H 2021 (Zhang et al., 2021b)	China	cohort study	1/1/2017	1/6/2020	3 years 5 months	97/41	6
Dai, 2021 (Dai et al., 2021)	China	case-control study	1/1/2017	1/9/2017	8 months	91/91	8
Zuo, Y 2021 (Zuo et al., 2021)	China	matched case-control study	1/1/2015	1/6/2017	2 years 5 months	74/74	6
Li, ZYQ 2021 (Li et al., 2021)	China	case–case–control study	1/9/2016	1/8/2019	3 years	66/66	6
Cao, 2022 (Cao et al., 2022)	China	case-control study	1/1/2016	1/12/2020	4 years 11 months	84/87	7
Chen, J 2022 (Chen et al., 2022)	China	case-control study	1/1/2012	1/12/2019	7 years 11 months	212/494	7
Wu, CY 2022 (Wu et al., 2022)	China	case-control study	1/1/2019	1/10/2021	2 years 9 months	68/146	7
Chen, Y 2022 (Chen et al., 2022)	China	cohort study	1/1/2011	1/12/2020	9 years 11 months	29/223	6
Wang, L 2022 (Wang et al., 2022)	China	case-control study	1/1/2015	1/6/2018	3 years 5 months	95/238	7
Lou, 2022 (Lou et al., 2022)	China	case-control study	1/3/2017	1/7/2017	4 months	422/948	6
Panda, 2022 (Panda et al., 2022)	India	case-control study	1/1/2020	31/12/2020	1 year	108/116	7
Liang, 2022 (Liang et al., 2022)	China	case-control study	1/1/2010	1/12/2018	8 years 11 months	56/47	7
Liu, C 2022 (Liu et al., 2022)	China	case-control study	1/1/2018	1/12/2020	2 years 11 months	74/373	7
Arslan, 2023 (Arslan et al., 2023)	Turkey	case–control study	1/1/2017	1/12/2019	2 years 11 months	44/76	6
Çölkesen, 2023 (Çölkesen et al., 2023)	Turkey	case-control study	1/1/2015	1/9/2019	4 years 8 months	132/150	6
Huang, 2023 (Huang et al., 2023)	China	case-case-control study	1/1/2016	1/12/2018	2 years 11 months	494/1357	6
Li, M 2023 (Li et al., 2023)	China	case-control study	1/1/2010	1/12/2019	9 years 11 months	268/419	8
Wang, HW 2023 (Wang et al., 2023)	China	case-control study	1/1/2018	1/1/2023	4 years 11 months	174/219	6
Cheng, Y 2024 (Cheng et al., 2024)	China	case-control study	1/1/2016	1/12/2020	4 years 11 months	50/84	7
Radu, 2024 (Radu et al., 2024)	Romania	case-control study	1/1/2022	31/3/2024	2 years 2 months	62/136	6

**Figure 1:**
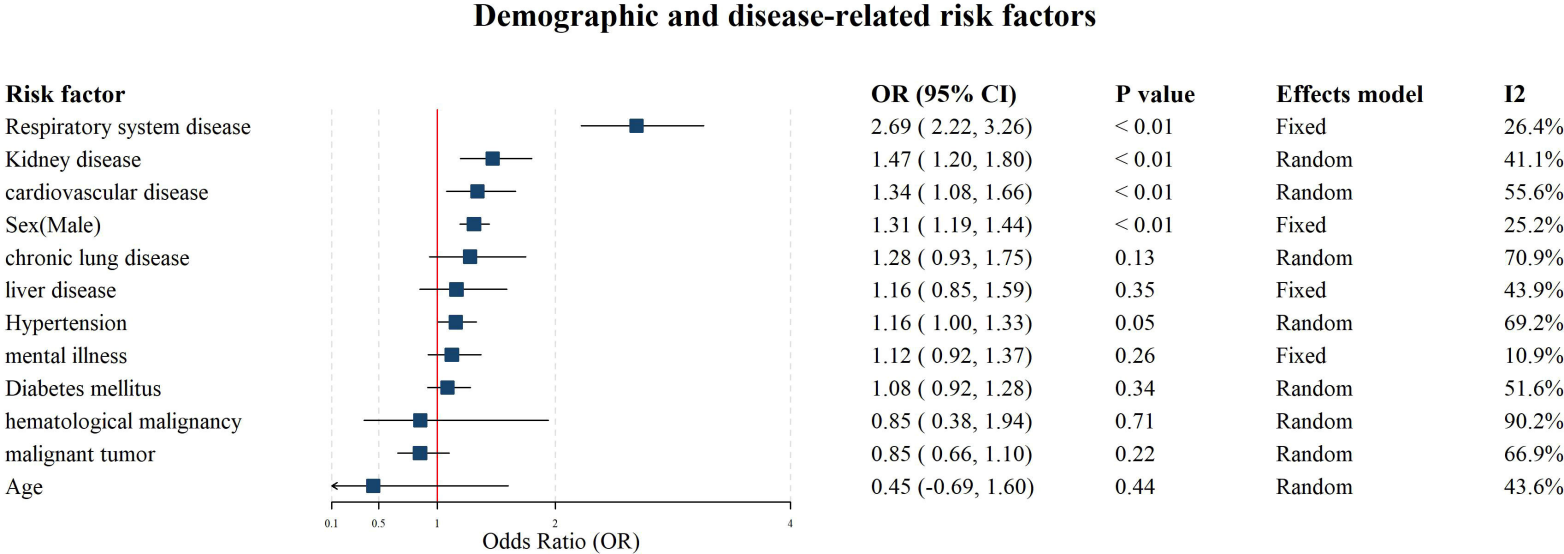
Forest plot for the meta-analysis on the associations between demographic characteristics, underlying diseases and CRKP infections. Mean difference (MD) is used for estimating association between age and carbapenem resistant Klebsiella pneumoniae infections using; the odds ratio (OR) is used for estimating the association between other factors and the infection.

**Figure 2:**
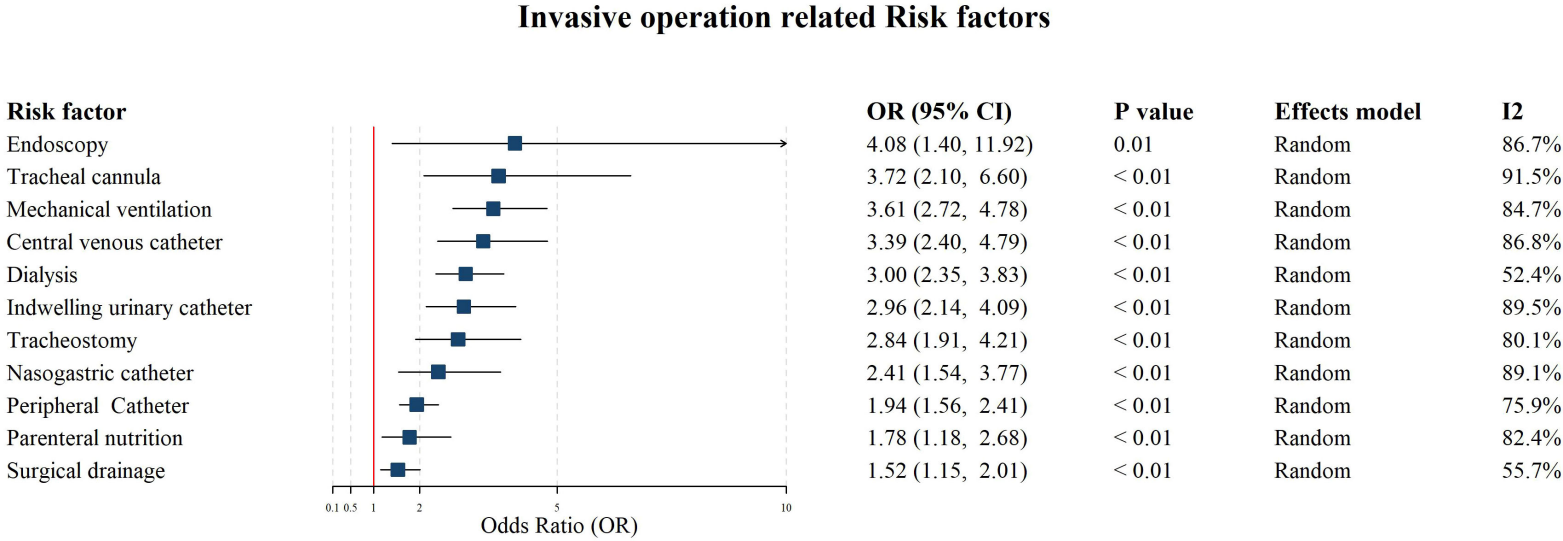
Forest plot for the meta-analysis on the associations between invasive procedures and CRKP infections.

**Figure 3:**
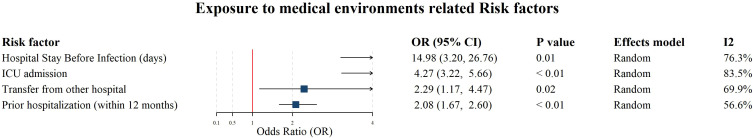
Forest plot for the meta-analysis on the associations between medical environment and CRKP infections. Mean difference (MD) estimation is used for estimating association between Hospital Stay Before Infection (days) and carbapenem resistant Klebsiella pneumoniae infections using; the odds ratio (OR) is used for estimating the association between other factors and the infection.

**Figure 4:**
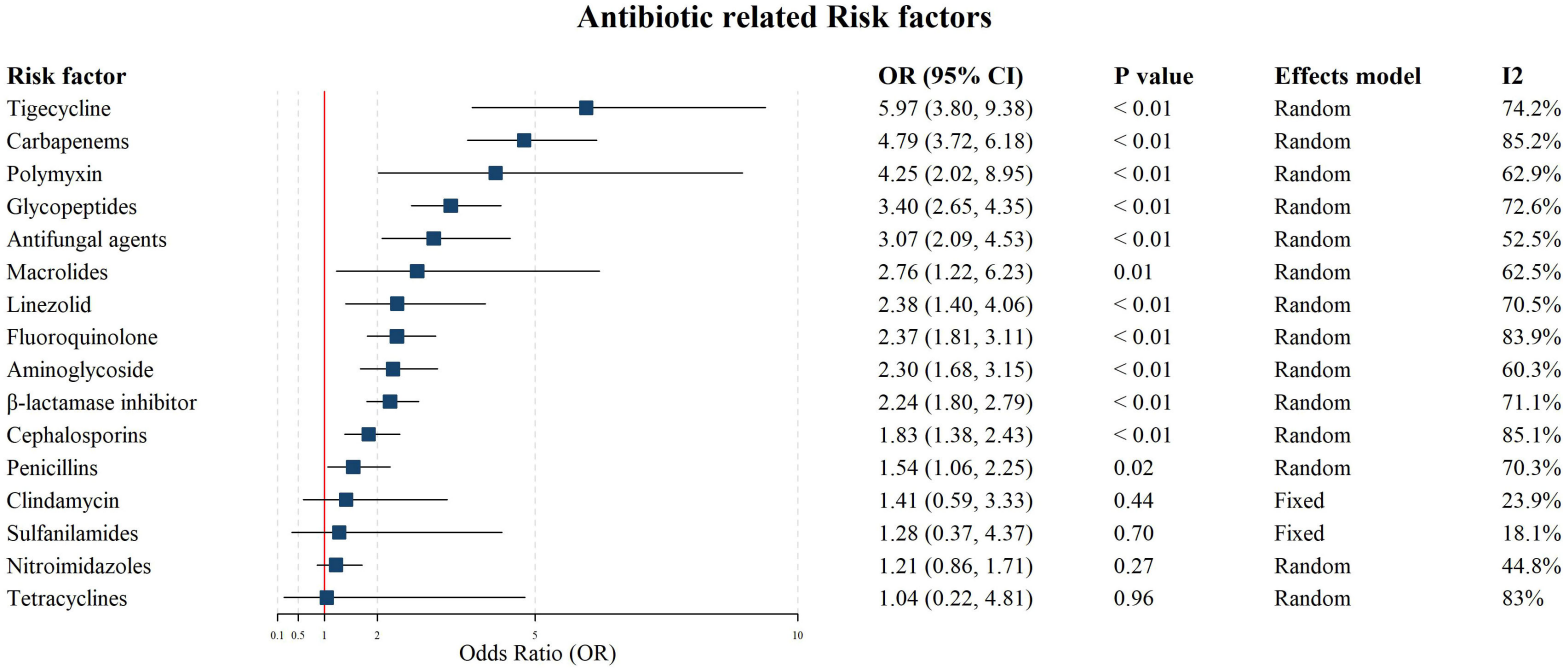
Forest plot for the meta-analysis on the associations between antibiotic exposures and CRKP infections.

A total of 43 factors were identified: 12 related to demographics or underlying diseases, 11 associated with invasive procedures, 4 linked to the medical environment, and 16 associated with antibiotic use. The meta-analysis results for all risk factors are illustrated in **Figures 1**–**4**.

### 3.3 Demographic characteristics and underlying diseases

The meta-analysis encompassed 39 studies investigating the age, sex-male, hypertension, diabetes mellitus, kidney disease, chronic lung disease, liver disease, respiratory system disease, mental illness, cardiovascular disease, malignant tumor, hematological malignancy, respectively. Among these, sex-male (OR: 1.31, 95% CI = 1.19-1.44), kidney diseases (OR: 1.47, 95% CI = 1.20-1.80), respiratory system diseases (OR: 2.69, 95% CI = 2.22-3.26) and cardiovascular diseases (CVD) (OR: 1.34, 95% CI = 1.08-1.66) were significantly associated with hospital-acquired CRKP infections. In contrast, hypertension, diabetes mellitus, chronic lung disease, liver disease, mental illness, malignant tumor, and hematological malignancy did not show significant associations (P ≥ 0.05). Significant heterogeneity is present in the hematological malignancy, chronic lung disease, hypertension, cardiovascular disease, and malignant tumor (*I^2^* = 90.20%, 70.90%, 69.2%, and 66.9%, respectively). Moreover, none of the risk factors exhibited significant publication bias (Egger’s test: P > 0.05, **Supplementary Figures 2**-**13**). The results of all meta-analyses involving demographic characteristics and underlying diseases are presented in **Figure 1**.

### 3.4 Invasive procedures

A meta-analysis involving 35 studies was performed on various invasive procedures, including endoscopy (OR: 4.08, 95% CI: 1.40 - 11.92), tracheal cannula (OR: 3.72, 95% CI: 2.10 - 6.60), mechanical ventilation (OR: 3.61, 95% CI: 2.72 - 4.78), central venous catheter (OR: 3.39, 95% CI: 2.40 - 4.79), dialysis (OR: 3.00, 95% CI: 2.35 - 3.83), indwelling urinary catheter (OR: 2.96, 95% CI: 2.14 - 4.09), tracheostomy (OR: 2.84, 95% CI: 1.91 - 4.21), nasogastric catheter (OR: 2.41, 95% CI: 1.54 - 3.77), peripheral catheter (OR: 1.94, 95% CI: 1.56 - 2.41), parenteral nutrition (OR: 1.78, 95% CI: 1.18 - 2.68), and surgical drainage (OR: 1.52, 95% CI: 1.15 - 2.01). The results demonstrated that all these invasive procedures significantly increased the risk of CRKP infection in hospitalized patients. Egger’s test results (P > 0.05; **Supplementary Figures 18**-**28**) indicated no significant publication bias across these studies. However, heterogeneity was observed among the studies for most factors (*I²* ≥ 50.0%, P < 0.05). The heterogeneity of invasive procedures including (Chronic lung disease, Mechanical ventilation, and ICU admission) was attributed to study design (**Supplementary Table 44**, cohort study vs. case-control study). The detailed meta-analysis results for invasive procedure-related factors are presented in **Figure 2**.

### 3.5 Medical environment

A total of 34 studies were included in the meta-analysis of medical environment-related factors, including hospital stay before infection (mean difference: 14.98, 95% CI = 3.20 - 26.76), ICU admission (OR: 4.27, 95% CI = 3.22 - 5.66), prior hospitalization (OR: 2.08, 95% CI: 1.67 - 2.60), and transfer from other hospital (OR: 2.29, 95% CI: 1.17 - 4.47). All these factors were significantly associated with hospital-acquired CRKP infections. Significant heterogeneity was detected among the studies (*I²* ≥ 50.0%, P < 0.05), while Egger’s test (P > 0.05; **Supplementary Figures 14**-**17**) showed no evidence of publication bias. The comprehensive results of meta-analyses for medical environmental factors are illustrated in **Figure 3**.

### 3.6 Antibiotics exposure

The meta-analysis on antibiotic exposure factors covered 16 types of antibiotics. Results showed that tigecycline (OR: 5.97, 95% CI = 3.80-9.38), carbapenems (OR: 4.79, 95% CI = 3.72-6.18), polymyxin(OR: 4.25, 95% CI = 2.02-8.95), glycopeptides(OR: 3.40, 95% CI = 2.65-4.35), antifungal agents(OR: 3.07, 95% CI = 2.09-4.53), macrolides (OR: 2.76, 95% CI = 1.22-6.23), linezolid (OR: 2.38, 95% CI = 1.40-4.06), fluoroquinolone (OR: 2.37, 95% CI = 1.81-3.11), aminoglycoside (OR: 2.30, 95% CI = 1.68-3.15), β-lactam/β-lactamase inhibitor (BL/BLI) (OR = 2.24, 95% CI = 1.80–2.79), cephalosporins(OR: 1.83, 95% CI = 1.38-2.43), and penicillin (OR: 1.54, 95% CI = 1.06-2.25) revealed that these antibiotic exposures are significantly associated with CRKP infection in hospitalized patients, which may be attributable to disease severity, prior colonization. In contrast, clindamycin (OR: 1.41, 95% CI: 0.59 - 3.33), sulfanilamide (OR: 1.28, 95% CI: 0.37 - 4.37), nitroimidazoles (OR: 1.21, 95% CI: 0.86 - 1.71), and tetracyclines (OR: 1.04, 95% CI: 0.22 - 4.81) did not show significant associations (P ≥ 0.05). Except clindamycin and sulfanilamide, analyses of other factors exhibited considerable heterogeneity (*I^2^* ≥ 50.0% and P<0.05). The high heterogeneity antibiotic-related (including “Cephalosporins”, “Fluoroquinolone”, and “Glycopeptides”) was driven by the time period (**Supplementary Table 44**, Pre-2015 vs. After-2015). Egger’s test indicated evidence of publication bias in studies on BL/BLI (P = 0.003) and penicillins (P = 0.029; **Supplementary Figures 29**-**44**). The detailed meta-analysis results for antibiotic exposure factors are presented in **Figure 4**.

### 3.7 Subgroup analysis

To explore the sources of heterogeneity and evaluate the impact of geographic location on the risk of hospital-acquired CRKP infections, subgroup meta-analyses were performed based on different geographical regions (Classification criteria in **Supplementary Material**, **Supplementary Table 1**.1). The subgroup analysis demonstrated that most risk factors remained significantly associated with hospital-acquired CRKP infections across diverse regions(**Supplementary Material**, **Supplementary Table 45**), including sex-male, kidney diseases, central venous catheterization, dialysis, tracheotomy, endoscopy, urethral catheterization, mechanical ventilation, parenteral nutrition, ICU admission, prior hospitalization, and exposure to antibiotics (glycosides, carbapenems, fluoroquinolones, macrolides, aminoglycosides, BL/BLI). There are differences in the effects of nasogastric catheter and penicillin on hospital-acquired CRKP infections between Eastern and Western populations (Penicillin has no effect on CRKP infection in Eastern populations, OR = 1.14 [0.59; 2.19], while Nasogastric catheter has no effect in Western populations, OR = 1.69 [0.42; 6.77]). Notable regional variations were observed in the magnitude of these associations between Western and Eastern populations. Sex-male was more strongly associated with CRKP infection in Western populations (OR = 1.49) compared to Eastern populations (OR = 1.19). Western populations exhibited higher risks with cephalosporins (OR _Western_ = 2.68 vs. OR _Eastern_ = 1.55) and fluoroquinolones (OR _Western_ = 3.58 vs. OR _Eastern_ = 1.89). In contrast, Eastern populations had significantly higher risks for invasive procedures such as dialysis (OR _Eastern_ = 4.47 vs. OR _Western_ = 2.03), mechanical ventilation (OR _Eastern_ = 4.22 vs. OR _Western_ = 2.34), and polymyxin use (OR _Eastern_ = 7.11 vs. OR _Western_ = 3.02). Respiratory diseases, hypertension, cardiovascular diseases, and nasogastric catheter use were exclusively linked to CRKP in Eastern populations, while liver disease increased risk by 60% in Western populations (OR: 1.60, 95% CI = 1.09-2.34) but showed no association in Eastern groups. Duration of ICU stay was a significant risk factor in Western populations but not in Eastern populations.

### 3.8 Sensitivity analysis

Sensitivity analyses were performed by sequentially excluding each included reference to evaluate the robustness of OR. The results remained stable for most factors (**Supplementary Material**, **Supplementary Tables 1**-**S42**), with exceptions observed for length of hospital stay before infection, transfer from other hospitals, chronic lung disease, hypertension, macrolide use, and penicillin use. These exceptions were attributed to high heterogeneity (*I²* > 75%) or small sample sizes in the excluded studies. This further supports the overall consistency of the main study conclusions.

To evaluate the impact of study quality on our results, we conducted a sensitivity analysis by excluding all low-quality studies (Newcastle-Ottawa Scale score ≤ 6), and compared the pooled odds ratios (OR), heterogeneity (*I^2^*). Exclusion of low-quality studies did not alter the overall directional trend of associations for most risk factors. However, notable changes in statistical significance were observed for two factors: the initially significant associations of Macrolides and Nasogastric catheter with the outcome were attenuated to non-significance after removing low-quality studies. Overall, the meta-analysis results demonstrated robustness and stability, with no substantial shifts in effect estimates following individual study removal.

## 4 Discussion

This comprehensive systematic review and meta-analysis evaluate the association between risk factors and hospital-acquired CRKP infections across Eastern and Western populations. The study contributes new evidence in two distinct areas: first, the study further validates and refines invasive procedure-related risk factors for CRKP infection, incorporating subtypes such as endoscopy and surgical drainage into the analysis to supplement evidence on CRKP risk stratification. Second, our analysis highlights the differences in risk factors for hospital-acquired CRKP infections between Eastern and Western populations.

The study identified a total of 31 risk factors for hospital-acquired CRKP infections, which can be classified into four categories: demographic information and underlying diseases, medical environment exposure, invasive procedures, and antibiotic use. With regard to demographic information and underlying diseases, interestingly, although our study did not find a statistically significant association between age and CRKP infection, the median age of CRKP-infected patients appeared to be lower than that of non-infected patients. This finding seems counterintuitive, as older individuals typically have more comorbidities, compromised immune function, and increased susceptibility to infections. However, similar observations have been reported in previous studies, suggesting that other factors might be at play (Wang et al., 2023). We identified sex-male as a potential risk factor for CRKP infection in our study, a finding not consistently observed in other studies (Li et al., 2020). We speculate that this discrepancy may be due to differences in the study populations included in our analysis compared with those in previous meta-analyses. For instance, the meta-analysis conducted by Li (Li et al., 2020) did not restrict the control group, which might have influenced the results. Respiratory diseases, cardiovascular diseases, and liver disease were significantly associated with hospital-acquired CRKP infections. Mechanistically, respiratory diseases increase susceptibility through multiple pathways: chronic lung impairment facilitates biofilm formation, while invasive interventions (e.g., tracheostomy, mechanical ventilation) disrupt mucosal barriers. CVD patients, often requiring prolonged hospitalization and vascular access, face elevated risks of nosocomial transmission. Liver disease, particularly cirrhosis, impairs reticuloendothelial function, compromising bacterial clearance and promoting systemic inflammation. Notably, liver disease showed no association in Eastern populations but increased risk by 60% in Western cohorts (OR = 1.60, 95% CI [1.09, 2.34]), possibly reflecting differences in etiologies (e.g., alcohol-related vs. viral hepatitis) or healthcare access.

In terms of medical environment, prolonged hospital stays, and ICU admission are identified as risk factors for CRKP infection. This conclusion is consistent with previous findings reported by Li and Liu (Liu et al., 2018; Li et al., 2020). This association is primarily attributed to two mechanisms: First, healthcare environments often serve as reservoirs for CRKP (Hu et al., 2022), with prolonged hospitalization increasing patient exposure to colonized surfaces, medical devices, or healthcare workers. Extended stays also enhance person-to-person transmission via contaminated hands or fomites, as demonstrated by cross-transmission studies in ward settings (Wu et al., 2023). Second, ICU patients typically have severe underlying conditions, requiring prolonged invasive interventions (e.g., mechanical ventilation, central venous catheters) and broad-spectrum antibiotic therapy. These interventions disrupt mucosal barriers, deplete protective microbiota, and create selective pressure for CRKP colonization. Additionally, the high acuity of care in ICUs often involves shared equipment and close patient-provider interactions, further elevating transmission risks. These findings highlight the role of healthcare infrastructure in driving CRKP epidemiology, underscoring the need for targeted infection control measures in high-risk settings.

Invasive medical interventions represent a critical risk factor for CRKP infection. Our meta-analysis identified mechanical ventilation, dialysis, endoscopy, and indwelling catheter use (urinary/central venous) as major drivers of CRKP acquisition, consistent with prior studies linking these procedures to healthcare-associated infections (Li et al., 2020; Zhu et al., 2020). These procedures not only disrupt the body’s normal physiological barriers, creating opportunities for opportunistic pathogens to invade, but also increase the risk of CRKP infection due to the potential for pathogen colonization on the surfaces of medical devices. Pathogens can form robust biofilms on these surfaces, which can lead to severe infections when the host’s immune defenses are compromised. For example, endoscopy—a newly identified risk factor (OR = 4.08, 95% CI = 1.40–11.92)—poses unique challenges due to the complex lumen structure of endoscopic devices. Microbiological studies have demonstrated that CRKP can form biofilms on the inner surfaces of endoscope channels, where nutrient-limited environments and intermittent use create ideal conditions for bacterial persistence. These biofilms are protected by extracellular polymeric substances, rendering them resistant to standard disinfection protocols and promoting cross-transmission across patients. Therefore, healthcare workers should strictly adhere to sterile techniques, carefully evaluate the indications for catheter placement, and enhance hand hygiene, environmental cleanliness, and the disinfection of medical equipment. Additionally, it is crucial to minimize unnecessary invasive procedures based on the patient’s clinical course. When such procedures are deemed necessary, they should be performed with strict control over the duration to reduce the risk of infection.

The relationship between antibiotic exposure and resistance is well-documented in studies (Van Duin et al., 2014; Hu et al., 2024). In this study, exposure to broad-spectrum antimicrobials—including fluoroquinolones, carbapenems, and cephalosporins—emerged as a key risk factor for CRKP infection, aligning with prior evidence of antibiotic-driven selection in K. pneumoniae populations (Zhu et al., 2023). These observed associations should not be interpreted simplistically as direct causal links, but rather more appropriately as surrogate markers reflecting underlying factors such as greater disease severity, prolonged hospital stay, and prior colonization with resistant strains. The patients receiving broad-spectrum antibiotics typically represent a population with more severe underlying conditions, longer hospitalizations, and higher exposure to healthcare-associated environments—all of which independently increase the risk of colonization and subsequent infection with multidrug-resistant organisms like CRKP. Antibiotic use, in this context, acts as a proxy for the overall burden of illness and healthcare exposure rather than being the sole or direct causal agent. Moreover, prior colonization with resistant strains often precedes clinical infection, and broad-spectrum antibiotics may simply facilitate the overgrowth of already colonized resistant bacteria rather than *de novo* resistance development. Thus, the observed association between antibiotic exposure and CRKP infection reflects a complex interplay of host vulnerability, healthcare exposure, and microbial ecology, rather than a unidirectional causal pathway.

The subgroup analysis of our study has revealed significant differences in the risk factors for CRKP infection among populations in different regions. The results show that a wide range of factors, including sex-male, kidney disease, central venous catheterization, dialysis, tracheostomy, endoscopy, urinary catheterization, mechanical ventilation, parenteral nutrition, ICU admission, history of hospitalization within the past year, and the use of various antibiotics (aminoglycosides, carbapenems, penicillins, fluoroquinolones, macrolides, aminoglycosides, BL/BLI), are still statistically associated with CRKP infection across different regional populations. These findings are consistent with previous studies, further confirming the significant role of these factors in CRKP infections. Notably, however, regional variation emerged in the strength of association for specific exposures. Cephalosporin (OR _Western_: 2.68, 95% CI: 1.89–3.76 vs. OR _Eastern_: 1.55, 95% CI: 1.12–2.15) and fluoroquinolone (OR _Western_: 3.58, 95% CI: 2.74–4.67 vs. OR _Eastern_: 1.89, 95% CI: 1.43–2.50) exposures exhibited stronger associations with CRKP in Western populations, potentially linked to higher community and hospital prescribing rates. This may be associated with the more frequent use of these antibiotics in Western countries, particularly in community and hospital settings. Conversely, invasive interventions (dialysis: OR _Eastern_ 4.47, 95% CI: 3.12–6.41 vs. OR _Western_ 2.03, 95% CI: 1.51–2.73; mechanical ventilation: OR _Eastern_ 4.22, 95% CI: 3.05–5.83 vs. OR _Western_ 2.34, 95% CI: 1.89–2.91) and polymyxin use (OR _Eastern_ 7.11, 95% CI: 5.09–9.93 vs. OR _Western_ 3.02, 95% CI: 2.18–4.18) showed stronger associations in Eastern populations. These disparities suggest that we should implement differentiated intervention measures in different regional populations. For example, in the Eastern population, more attention should be paid to the risk of CRKP infection caused by invasive procedures, while in the Western population, more attention should be paid to the rational use of antibiotics.

Our meta-analysis identified substantial to high heterogeneity (I2>70%) across most risk factors, which we contextualized through subgroup analyses of study design and time period. For exposures like mechanical ventilation and ICU admission, heterogeneity was largely driven by study design: case-control studies (prone to recall or selection bias) contributed to elevated *I^2^*, whereas cohort studies (with prospective exposure measurement) exhibited low heterogeneity. This highlights the importance of study design in estimating associations for healthcare-related risk factors, as observational biases may skew effect sizes in non-cohort designs. Temporal shifts (Pre-2015 vs. After-2015) also emerged as a key heterogeneity source for antimicrobial and device-related exposures. For example, reduced heterogeneity in post-2015 subgroups of cephalosporins and fluoroquinolones aligns with global efforts to restrict broad-spectrum antibiotic use—suggesting that evolving clinical guidelines have standardized exposure patterns and reduced between-study variability.

Our study also has certain limitations. Reliance on univariate analyses instead of multivariate models may have introduced residual confounding. A subset of the included studies (primarily those published pre-2015) did not perform multivariate regression analyses, rendering the extraction of adjusted odds ratio (aOR) data objectively unfeasible. Furthermore, substantial methodological heterogeneity was observed in confounder adjustment strategies across the remaining studies—marked differences existed in both the types and quantities of covariates adjusted for CRKP infection risk. Statistically, the pooling of effect sizes relies on the homogeneity of individual study estimates. The aORs derived from disparate adjustment frameworks reflect association strengths under distinct analytical conditions; pooling such heterogeneous aORs would introduce methodological confounding bias and yield misleading results. Therefore, our primary analysis opted to pool unadjusted ORs to ensure the reliability and comparability of the synthesized findings. Nevertheless, the ORs synthesized in our study may still be subject to bias, and the pooled estimates should not be fully utilized for individual-level risk prediction.

## 5 Conclusions

This meta-analysis report endoscopy and surgical drainage as distinct subtypes of invasive procedural factors associated with hospital-acquired CRKP infection and highlights regional differences in associated factors. Future prevention strategies may benefit from tailoring to regional contexts, e.g., optimizing invasive procedure use in Eastern populations and antimicrobial stewardship in Western populations—though these recommendations are based on observational evidence and require validation in prospective studies.

## Data Availability

The data analyzed in this study is subject to the following licenses/restrictions: The datasets used and/or analyzed during the current study are available from the corresponding author on reasonable request. Requests to access these datasets should be directed to 20211020187@fudan.edu.cn.
