# Supplementary material for: Risk factors for carbapenem-resistant Klebsiella pneumoniae infection in hospitalized patients: a meta-analysis
Source: Front Cell Infect Microbiol. 2026 Mar 10;16:1717419. doi: 10.3389/fcimb.2026.1717419 (PMC13008901; doi:10.3389/fcimb.2026.1717419)
Supplement: Supplementary file 1 [file Table1.docx]

Catelog list

[Figure S1 Flow chart of the search for relevant references 6](#_Toc221180311)

[Retrieval formula 7](#_Toc221180312)

[Retrieval formula in Pubmed (Medline) 7](#_Toc221180313)

[Retrieval formula in Web of Science 9](#_Toc221180314)

[Retrieval formula in Cochrane Library 10](#_Toc221180315)

[Retrieval formula in Embase (OVID) 11](#_Toc221180316)

[Figure S2 Funnel plot for publication bias of association between Age and CRKP infections 13](#_Toc221180317)

[Figure S3 Funnel plot for publication bias of association between Sex and CRKP infections 14](#_Toc221180318)

[Figure S4 Funnel plot for publication bias of association between Respiratory system disease and CRKP infections 15](#_Toc221180319)

[Figure S5 Funnel plot for publication bias of association between kidney disease and CRKP infections 16](#_Toc221180320)

[Figure S6 Funnel plot for publication bias of association between cardiovascular disease and CRKP infections 17](#_Toc221180321)

[Figure S7 Funnel plot for publication bias of association between- chronic lung disease and CRKP infections 18](#_Toc221180322)

[Figure S8 Funnel plot for publication bias of association between liver disease and CRKP infections 19](#_Toc221180323)

[Figure S9 Funnel plot for publication bias of association between Hypertension and CRKP infections 20](#_Toc221180324)

[Figure S10 Funnel plot for publication bias of association between mental illness and CRKP infections 21](#_Toc221180325)

[Figure S11 Funnel plot for publication bias of association between- Diabetes mellitus and CRKP infections 22](#_Toc221180326)

[Figure S12 Funnel plot for publication bias of association between- hematological malignancy and CRKP infections 23](#_Toc221180327)

[Figure S13 Funnel plot for publication bias of association between malignant tumor and CRKP infections 24](#_Toc221180328)

[Figure S14 Funnel plot for publication bias of association between Hospital Stay Before Infection (days) and CRKP infections 25](#_Toc221180329)

[Figure S15 Funnel plot for publication bias of association between-ICU admission and CRKP infections 26](#_Toc221180330)

[Figure S16 Funnel plot for publication bias of association between Transfer from other hospital and CRKP infections 27](#_Toc221180331)

[Figure S17 Funnel plot for publication bias of association between Prior hospitalization (within 12 months) and CRKP infections 28](#_Toc221180332)

[Figure S18 Funnel plot for publication bias of association between- Nasogastric catheter and CRKP infections 29](#_Toc221180333)

[Figure S19 Funnel plot for publication bias of association between- Parenteral nutrition and CRKP infections 30](#_Toc221180334)

[Figure S20 Funnel plot for publication bias of association between Mechanical ventilation and CRKP infections 31](#_Toc221180335)

[Figure S21 Funnel plot for publication bias of association between- Indwelling urinary catheter and CRKP infections 32](#_Toc221180336)

[Figure S22 Funnel plot for publication bias of association between Endoscopy and CRKP infections 33](#_Toc221180337)

[Figure S23 Funnel plot for publication bias of association between Tracheal cannula and CRKP infections 34](#_Toc221180338)

[Figure S24 Funnel plot for publication bias of association between Tracheostomy and CRKP infections 35](#_Toc221180339)

[Figure S25 Funnel plot for publication bias of association between Dialysis and CRKP infections 36](#_Toc221180340)

[Figure S26 Funnel plot for publication bias of association between Surgical drainage and CRKP infections 37](#_Toc221180341)

[Figure S27 Funnel plot for publication bias of association between Peripheral Catheter and CRKP infections 38](#_Toc221180342)

[Figure S28 Funnel plot for publication bias of association between Central venous catheter and CRKP infections 39](#_Toc221180343)

[Figure S29 Funnel plot for publication bias of association betweenβ-lactamase inhibitor and CRKP infections 40](#_Toc221180344)

[Figure S30 Funnel plot for publication bias of association between Aminoglycoside and CRKP infections 41](#_Toc221180345)

[Figure S31 Funnel plot for publication bias of association between Macrolides and CRKP infections 42](#_Toc221180346)

[Figure S32 Funnel plot for publication bias of association between Polymyxin and CRKP infections 43](#_Toc221180347)

[Figure S33 Funnel plot for publication bias of association between Linezolid and CRKP infections 44](#_Toc221180348)

[Figure S35 Funnel plot for publication bias of association between Glycopeptides and CRKP infections 45](#_Toc221180349)

[Figure S36 Funnel plot for publication bias of association between Sulfanilamides and CRKP infections 46](#_Toc221180350)

[Figure S37 Funnel plot for publication bias of association between Antifungal agents and CRKP infections 47](#_Toc221180351)

[Figure S38 Funnel plot for publication bias of association between Clindamycin and CRKP infections 48](#_Toc221180352)

[Figure S39 Funnel plot for publication bias of association between Penicillins and CRKP infections 49](#_Toc221180353)

[Figure S40 Funnel plot for publication bias of association between Tigecycline and CRKP infections 50](#_Toc221180354)

[Figure S41 Funnel plot for publication bias of association between Carbapenems and CRKP infections 51](#_Toc221180355)

[Figure S42 Funnel plot for publication bias of association between Tetracyclines and CRKP infections 52](#_Toc221180356)

[Figure S43 Funnel plot for publication bias of association between Cephalosporins and CRKP infections 53](#_Toc221180357)

[Figure S44 Funnel plot for publication bias of association between Nitroimidazoles and CRKP infections 54](#_Toc221180358)

[Table S1 Sensitivity analysis for associations between Age and CRKP infections in enrolled studies 55](#_Toc221180359)

[Table S2 Sensitivity analysis for associations between sex and CRKP infections in enrolled studies 56](#_Toc221180360)

[Table S3 Sensitivity analysis for associations between ICU admission and CRKP infections in enrolled studies 58](#_Toc221180361)

[Table S4 Sensitivity analysis for associations between Hospital Stay Before Infection (days) and CRKP infections in enrolled studies 60](#_Toc221180362)

[Table 5 Sensitivity analysis for associations between Transfer from other hospital and CRKP infections in enrolled studies 61](#_Toc221180363)

[Table S6 Sensitivity analysis for associations between Prior hospitalization (within 12 months)and CRKP infections in enrolled studies 62](#_Toc221180364)

[Table S7 Sensitivity analysis for associations between prior hospitalization (within 12 months) and CRKP infections in enrolled studies 63](#_Toc221180365)

[Table S8 Sensitivity analysis for associations between Nasogastric catheter and CRKP infections in enrolled studies 64](#_Toc221180366)

[Table S9 Sensitivity analysis for associations between Parenteral nutrition and CRKP infections in enrolled studies 65](#_Toc221180367)

[Table S10 Sensitivity analysis for associations between Mechanical ventilation and CRKP infections in enrolled studies 66](#_Toc221180368)

[Table S11 Sensitivity analysis for associations between Indwelling urinary catheter and CRKP infections in enrolled studies 68](#_Toc221180369)

[Table S12 Sensitivity analysis for associations between Endoscopy and CRKP infections in enrolled studies 70](#_Toc221180370)

[Table S13 Sensitivity analysis for associations between Tracheal cannula and CRKP infections in enrolled studies 71](#_Toc221180371)

[Table S14 Sensitivity analysis for associations between Tracheostomy and CRKP infections in enrolled studies 72](#_Toc221180372)

[Table S15 Sensitivity analysis for associations between Dialysis and CRKP infections in enrolled studies 73](#_Toc221180373)

[Table S16 Sensitivity analysis for associations between Surgical drainage and CRKP infections in enrolled studies 74](#_Toc221180374)

[Table S17 Sensitivity analysis for associations between Peripheral Catheter and CRKP infections in enrolled studies 75](#_Toc221180375)

[Table S18 Sensitivity analysis for associations between Central venous catheter and CRKP infections in enrolled studies 76](#_Toc221180376)

[Table S19 Sensitivity analysis for associations between cardiovascular disease and CRKP infections in enrolled studies 78](#_Toc221180377)

[Table S20 Sensitivity analysis for associations between Diabetes mellitus and CRKP infections in enrolled studies 80](#_Toc221180378)

[Table S21 Sensitivity analysis for associations between Kidney disease and CRKP infections in enrolled studies 82](#_Toc221180379)

[Table S22 Sensitivity analysis for associations between chronic lung disease and CRKP infections in enrolled studies 84](#_Toc221180380)

[Table S23 Sensitivity analysis for associations between mental illness and CRKP infections in enrolled studies 85](#_Toc221180381)

[Table S24 Sensitivity analysis for associations between Respiratory system disease and CRKP infections in enrolled studies 86](#_Toc221180382)

[Table S25 Sensitivity analysis for associations between Hypertension and CRKP infections in enrolled studies 87](#_Toc221180383)

[Table S26 Sensitivity analysis for associations between liver disease and CRKP infections in enrolled studies 88](#_Toc221180384)

[Table S27 Sensitivity analysis for associations between β-lactamase inhibitor and CRKP infections in enrolled studies 89](#_Toc221180385)

[Table S28 Sensitivity analysis for associations between Aminoglycoside and CRKP infections in enrolled studies 91](#_Toc221180386)

[Table S29 Sensitivity analysis for associations between Macrolides and CRKP infections in enrolled studies 93](#_Toc221180387)

[Table S30 Sensitivity analysis for associations between Polymyxin and CRKP infections in enrolled studies 94](#_Toc221180388)

[Table S31 Sensitivity analysis for associations between Linezolid and CRKP infections in enrolled studies 95](#_Toc221180389)

[Table S32 Sensitivity analysis for associations between Fluoroquinolone and CRKP infections in enrolled studies 96](#_Toc221180390)

[Table S33 Sensitivity analysis for associations between Tigecycline and CRKP infections in enrolled studies 98](#_Toc221180391)

[Table S34 Sensitivity analysis for associations between Sulfanilamides and CRKP infections in enrolled studies 99](#_Toc221180392)

[Table S35 Sensitivity analysis for associations between Antifungal agents and CRKP infections in enrolled studies 100](#_Toc221180393)

[Table S36 Sensitivity analysis for associations between Clindamycin and CRKP infections in enrolled studies 101](#_Toc221180394)

[Table S37 Sensitivity analysis for associations between Penicillins and CRKP infections in enrolled studies 102](#_Toc221180395)

[Table S38 Sensitivity analysis for associations between Tetracyclines and CRKP infections in enrolled studies 103](#_Toc221180396)

[Table S39 Sensitivity analysis for associations between Carbapenems and CRKP infections in enrolled studies 104](#_Toc221180397)

[Table S40 Sensitivity analysis for associations between Glycopeptides and CRKP infections in enrolled studies 106](#_Toc221180398)

[Table S41 Sensitivity analysis for associations between Cephalosporins and CRKP infections in enrolled studies 108](#_Toc221180399)

[Table S42 Sensitivity analysis for associations between Nitroimidazoles and CRKP infections in enrolled studies 110](#_Toc221180400)

[Table S43 Pooled results of risk factors for CRKP infection after excluding low-quality studies 111](#_Toc221180401)

[Table S44 Pooled Odds Ratios and Heterogeneity (I²) of Risk Factors for CRKP infections by Time Period and Study Design 113](#_Toc221180402)

[Table S45 Pooled Odds Ratios and Heterogeneity (I²) of Risk Factors for CRKP infections by region 114](#_Toc221180403)


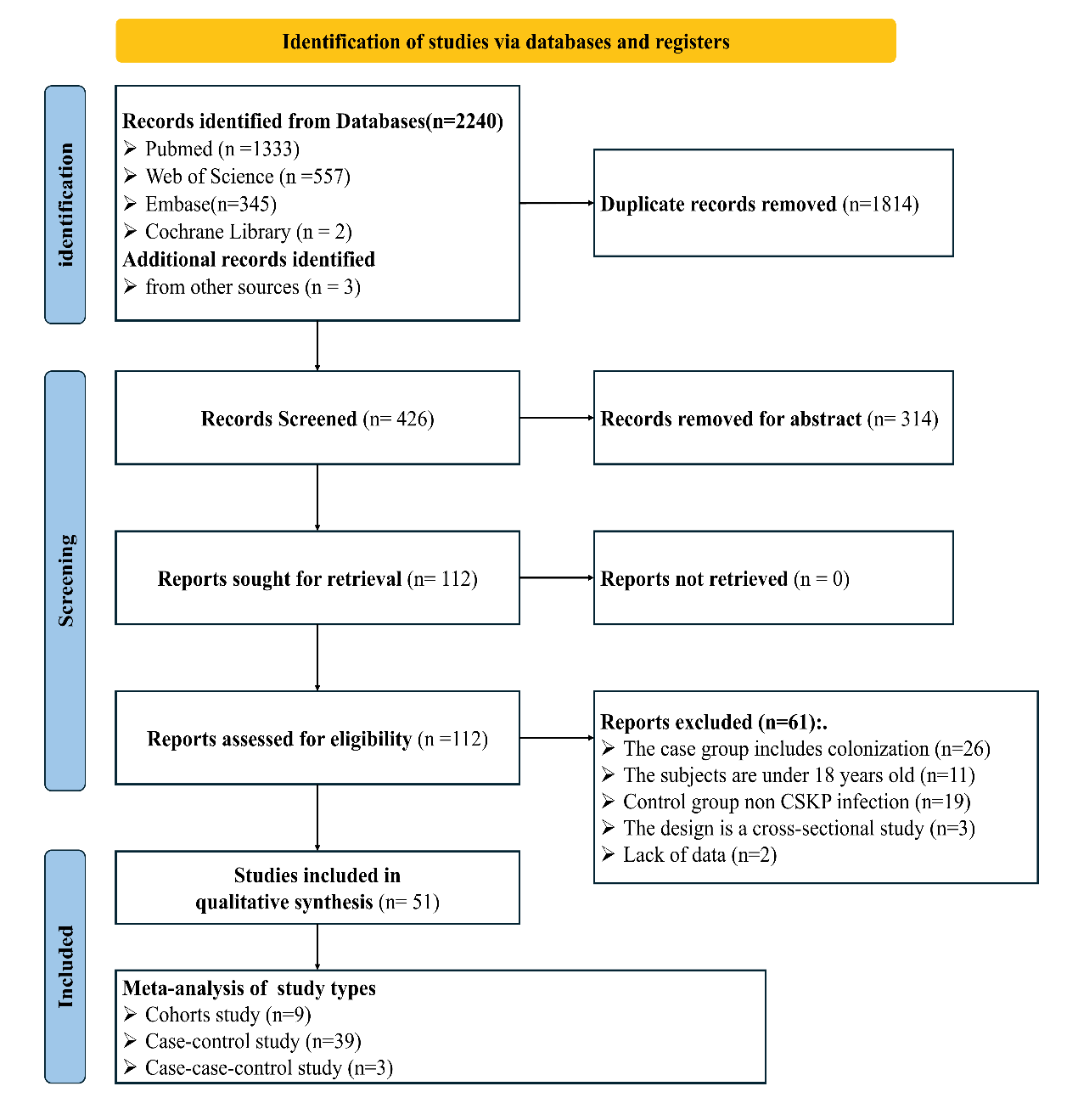


# **Figure S1 Flow chart of the search for relevant references**

# **Retrieval formula**

## **Retrieval formula in Pubmed (Medline)**

(

(

"klebsiella pneumoniae"[All Fields] OR "klebsiella pneumoniae"[MeSH Terms] OR "klebsiella"[All Fields] OR "klebsiella"[MeSH Terms]

)

AND

(

"carbapenem resistant"[All Fields] OR "carbapenem-resistant"[All Fields] OR"multidrug resistant"[All Fields] OR "extensively drug resistant"[All Fields] OR "pandrug resistant"[All Fields] OR carbapenem OR imipenem OR meropenem OR ertapenem OR carbapenemase-producing

)

AND

(

Causality[Mesh] OR cause[tiab] OR causes[tiab] OR Epidemiology[Mesh] OR factor[tiab] OR factors[tiab] OR Incidence[Mesh] OR incidence[tiab] OR incidences[tiab] OR Prevalence[Mesh] OR prevalence[tiab] OR Probability[Mesh] OR probability[tiab] OR Risk[Mesh] OR risk[tiab] OR risks[tiab] OR Risk Assessment[Mesh] OR risk assessment[tiab] OR risk assessments[tiab] OR risk factors[mesh] OR risk factor[tiab] OR risk factors[tiab] OR predict*[tiab] OR health correlates[tiab] OR correlates, health[tiab]

)

AND

(

"infection"[All Fields] OR "infections"[MeSH Terms] OR "bloodstream infection"[All Fields] OR "sepsis"[MeSH Terms] OR septic shock[All Fields] OR septicemia[All Fields] OR septic*[All Fields] OR urosepsis[All Fields] OR "urinary tract infection"[All Fields] OR "urinary tract infections"[MeSH Terms] OR "pneumonia"[All Fields] OR "pneumonia"[MeSH Terms] OR "ventilator-associated pneumonia"[All Fields] OR "trauma infection"[All Fields] OR "wounds and injuries"[MeSH Terms] OR "catheter infection"[All Fields] OR "catheter related infections"[MeSH Terms]

)

AND

(

"cohort studies"[MeSH Terms] OR "case-control studies"[MeSH Terms]

)

)

## **Retrieval formula in Web of Science**

TS=(klebsiella pneumoniae)

AND

TS=(carbapenem resistant OR carbapenem-resistant OR carbapenem OR imipenem OR meropenem OR ertapenem OR carbapenemase-producing)

AND

TS=(cause OR causes OR factor OR factors OR incidence OR incidences OR prevalence OR probability OR risks OR risk OR risk assessment OR risk assessments OR risk factors OR predict OR health correlates OR correlates, health)

AND

TS=(infection OR infections OR bloodstream infection OR sepsis OR septic shock OR septicemia OR septic OR urosepsis OR urinary tract infection OR urinary tract infections OR pneumonia OR ventilator-associated pneumonia OR trauma infection OR wounds and injuries OR catheter infection OR catheter related infections)

AND

TI=(case-control or case-cohort or case-case or case crossover or Retrospective OR cohort or prospective or incidence or follow-up or longitudinal)

AND

**NOT (TS=meta-analysis OR TS=review)**

## **Retrieval formula in Cochrane Library**

ID Search Hits

#1 (klebsiella pneumoniae):ti,ab,kw (Word variations have been searched) 816

#2 (carbapenem resistant):ti,ab,kw (Word variations have been searched) 501

#3 Risk Factors 109399

#4 #1 and #2 and #3 9

## **Retrieval formula in Embase (OVID)**

#1 ((klebsiella pneumoniae) and (carbapenem resistant or carbapenem or imipenem or meropenem or ertapenem) and (cause or causes or factor or factors or incidence or incidences or prevalence or probability or risks or risk or risk assessment or risk assessments or risk factors or predict or health correlates or correlates, health)).tw.
#2 (case-control or case-cohort or case-case or case crossover or Retrospective).ti.
#3 (cohort or prospective or incidence or follow-up or longitudinal).ti.

#1 and ( #2 OR #3)

**Table S1.1 Subgroup Analysis Classification by Eastern and Western Populations**

| Category | Specific Regions |
| --- | --- |
| Eastern Population-related Regions | China, India |
| Western Population-related Regions | United States of America, Greece, Italy, Romania, Turkey, Brazil, Colombia, Israel, Puerto Rico |


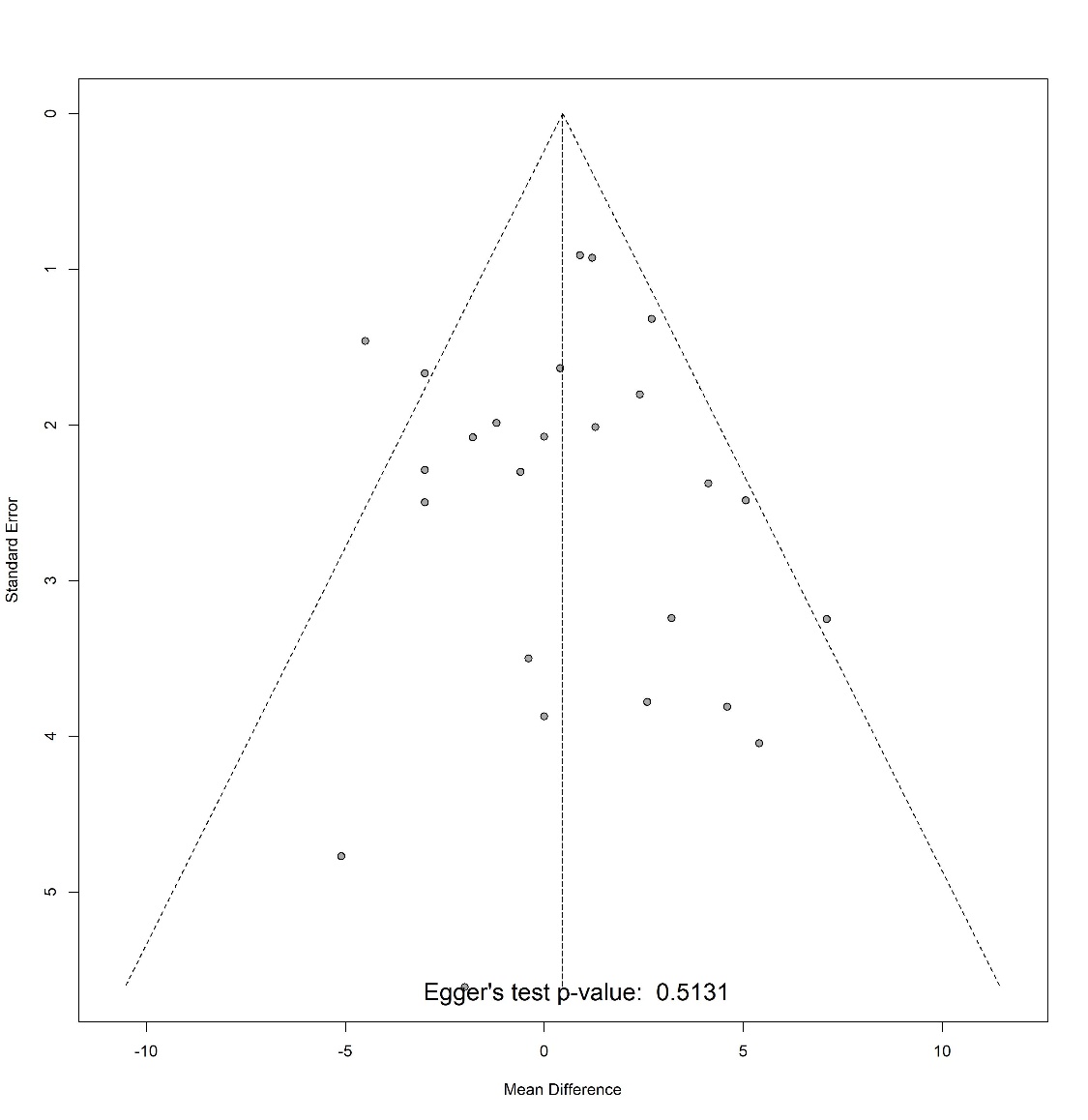


# **Figure S2 Funnel plot for publication bias of association between Age and CRKP infections**

# **Figure S3 Funnel plot for publication bias of association between Sex and CRKP infections**


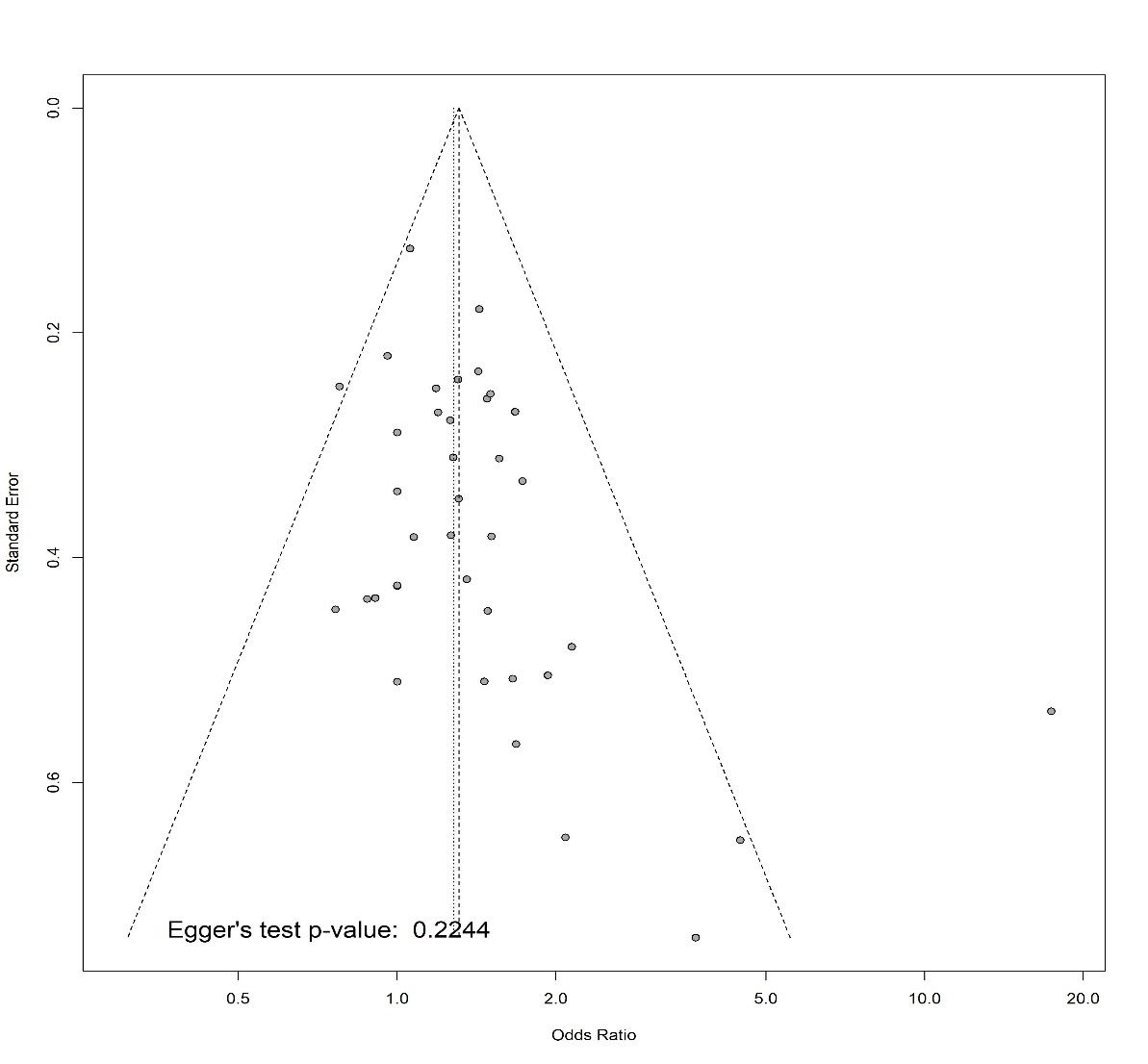


# **Figure S4 Funnel plot for publication bias of association between Respiratory system disease and CRKP infections**


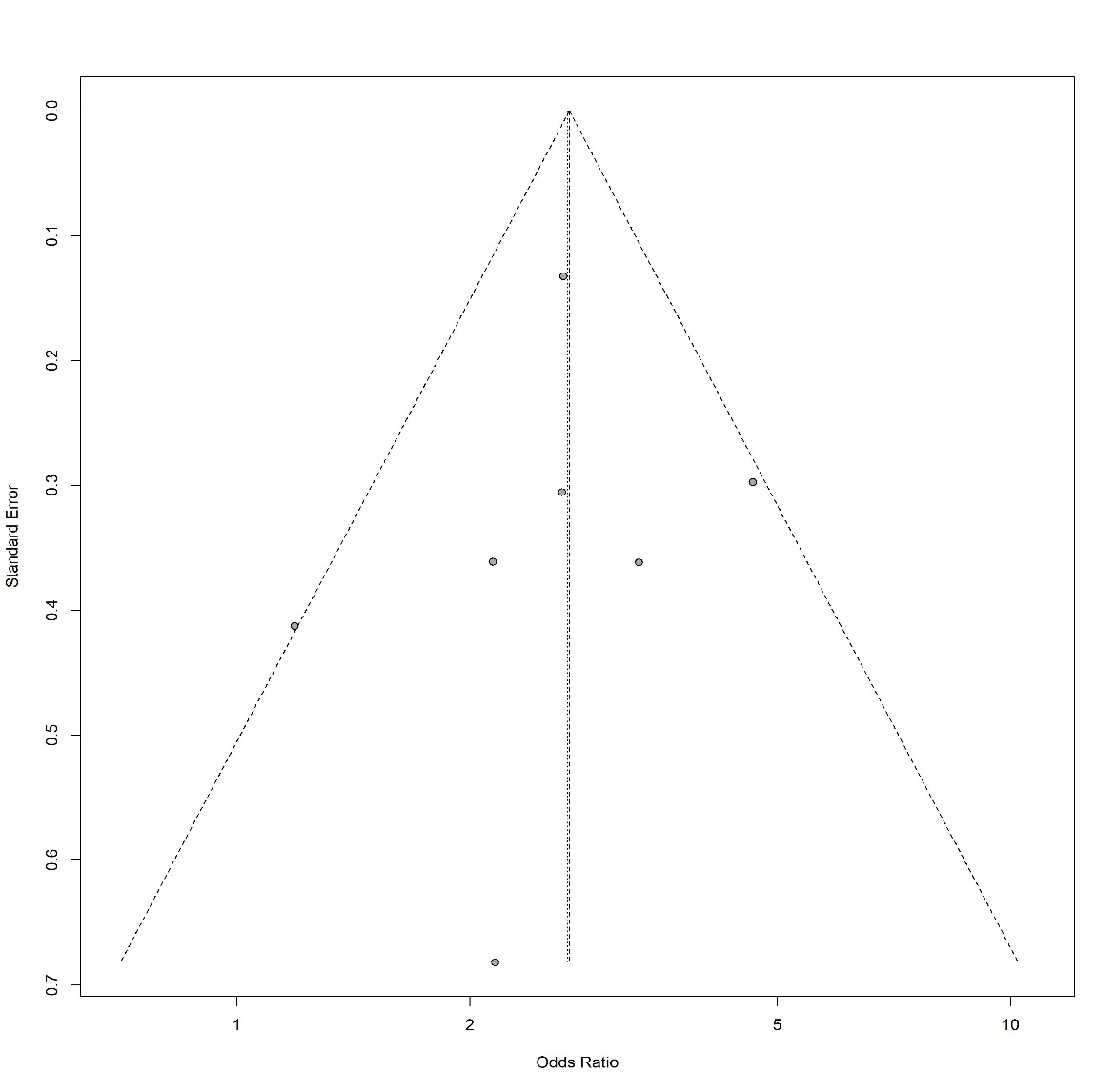


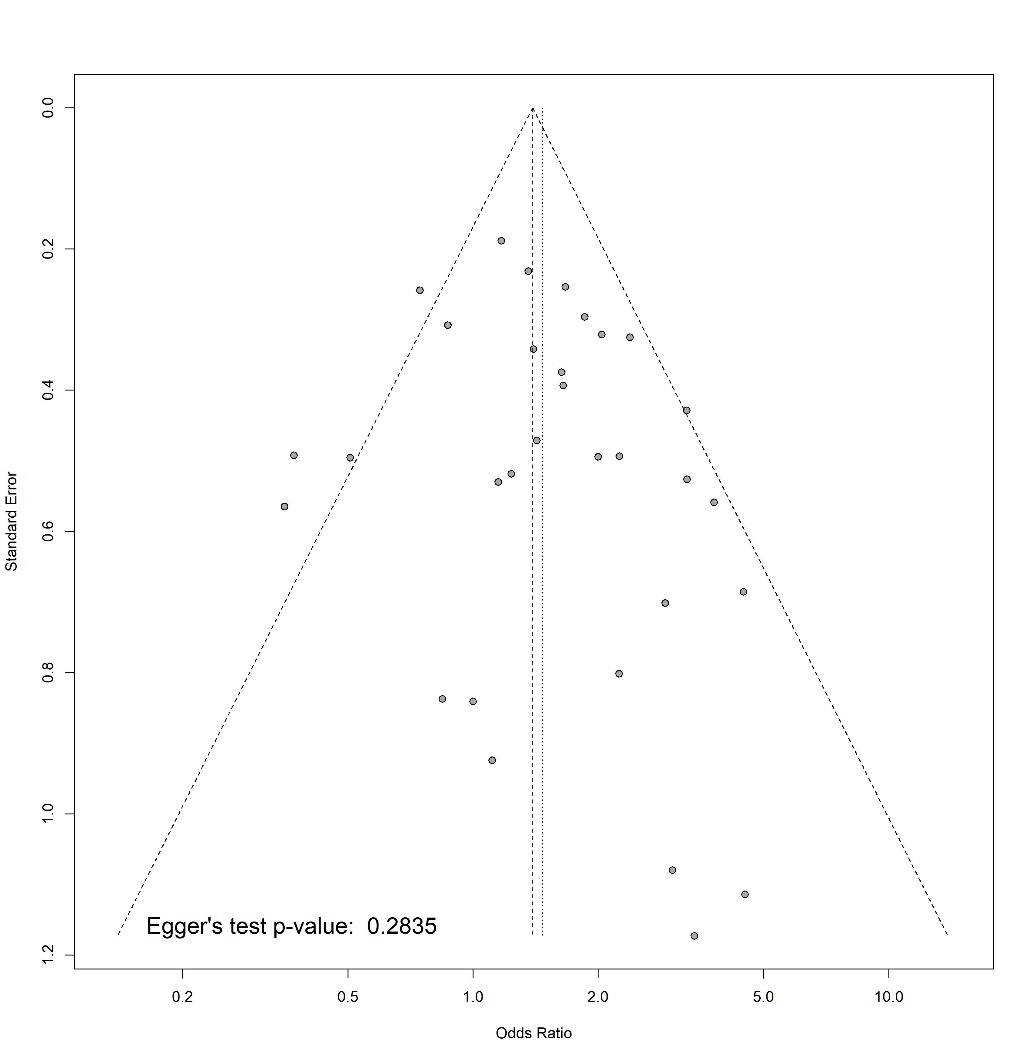


# **Figure S5 Funnel plot for publication bias of association between kidney disease and CRKP infections**


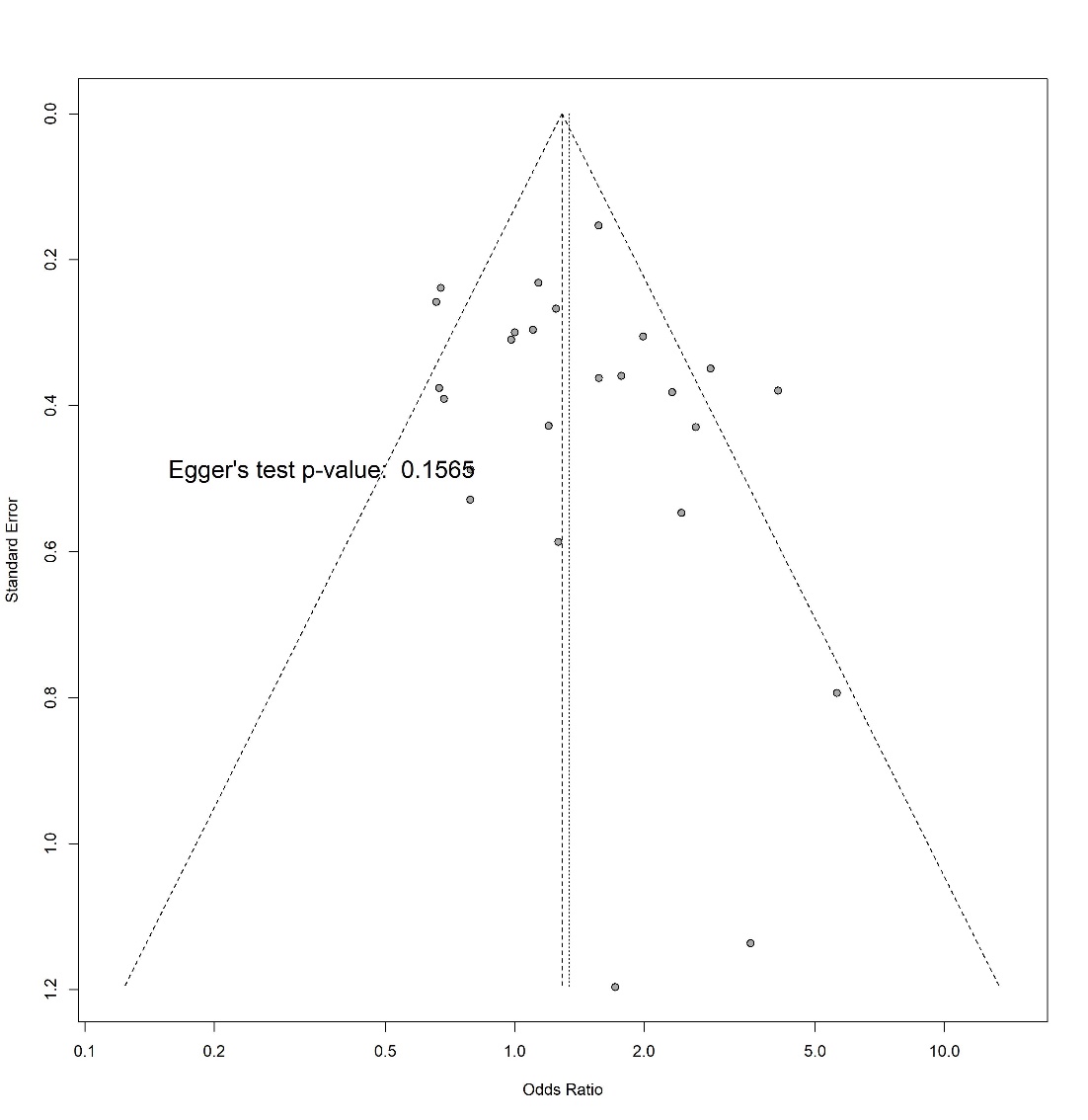


# **Figure S6 Funnel plot for publication bias of association between cardiovascular disease and CRKP infections**


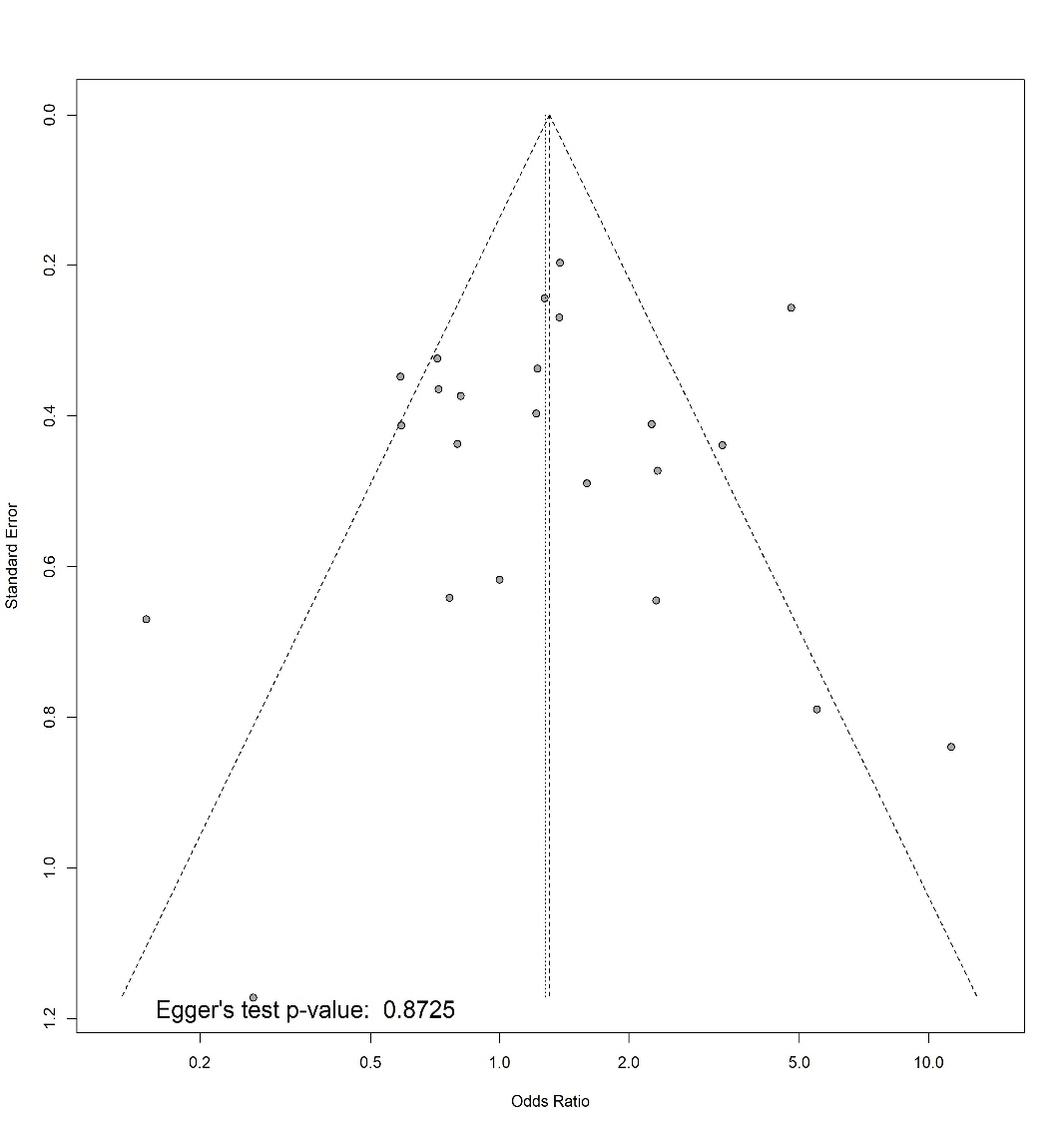


# **Figure S7 Funnel plot for publication bias of association between- chronic lung disease and CRKP infections**


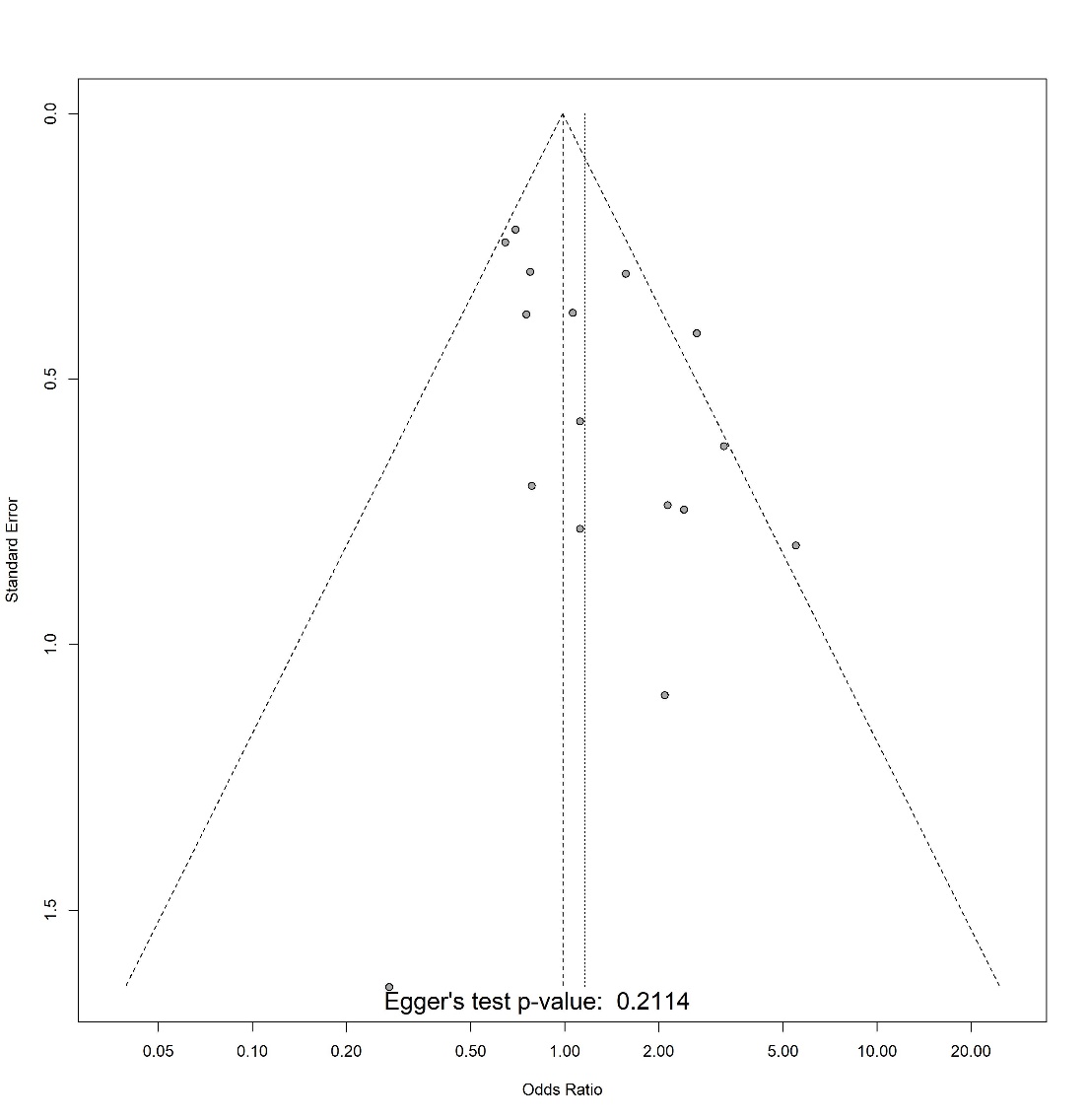


# **Figure S8 Funnel plot for publication bias of association between liver disease and CRKP infections**


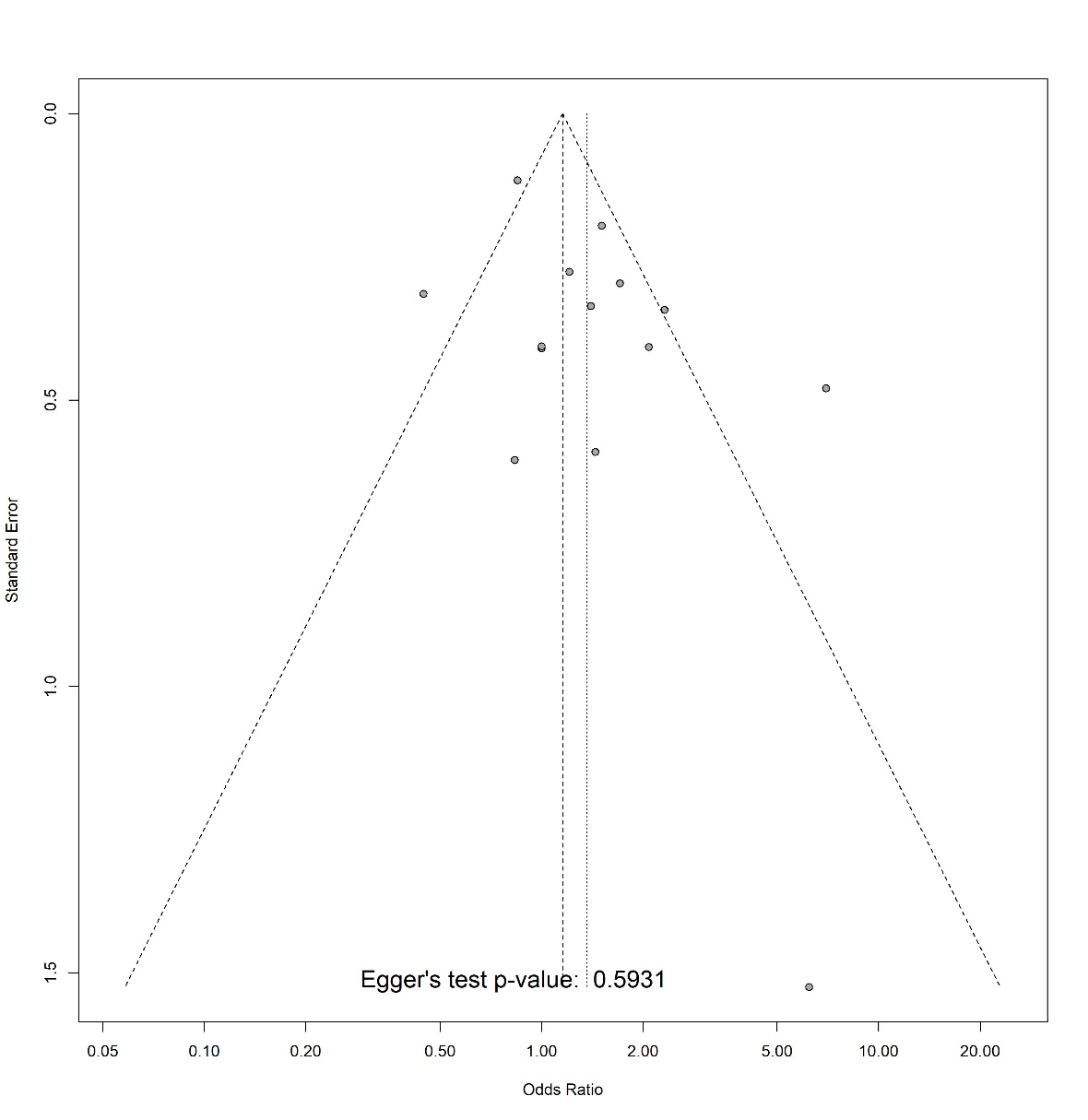


# **Figure S9 Funnel plot for publication bias of association between Hypertension and CRKP infections**


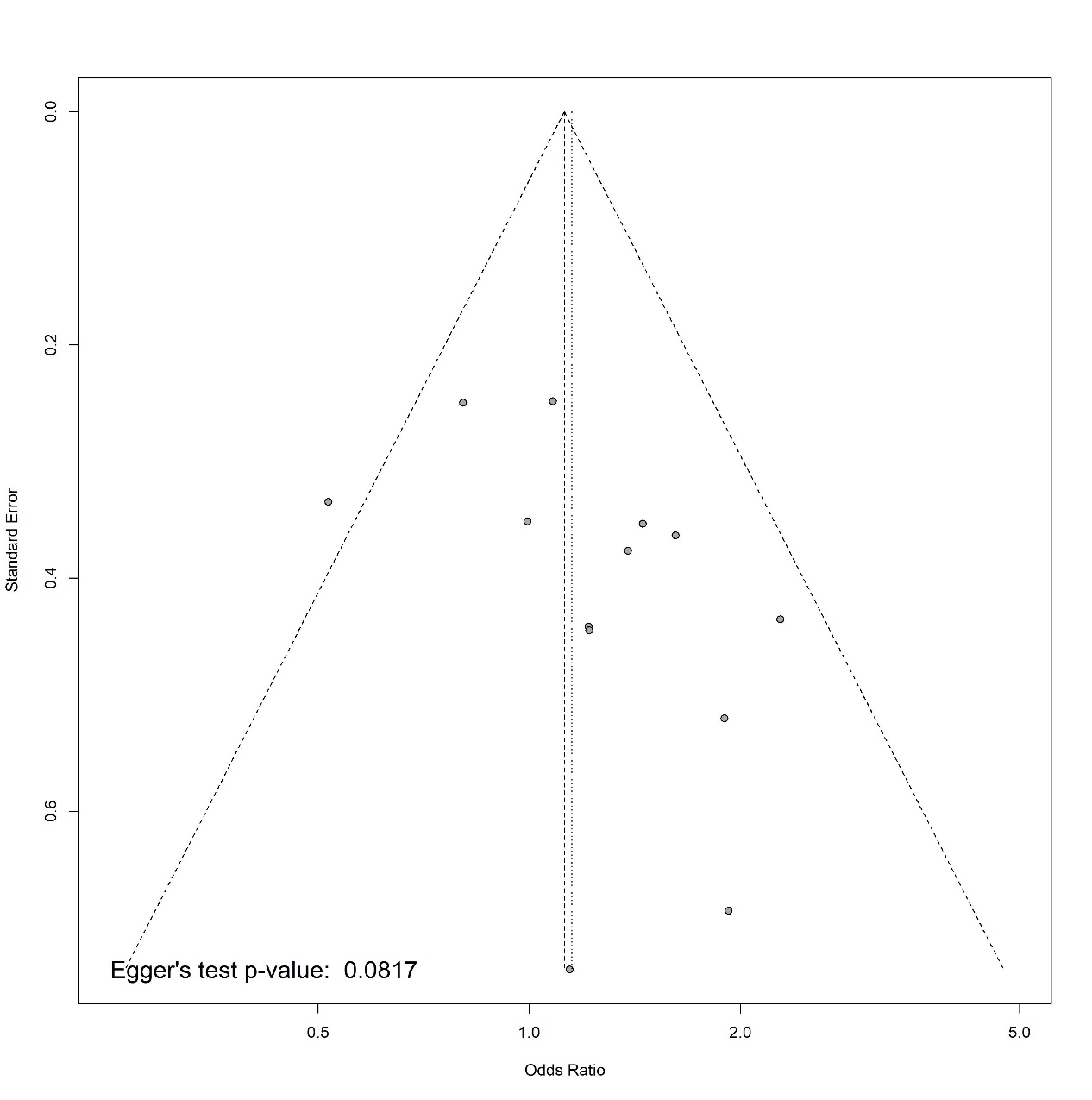

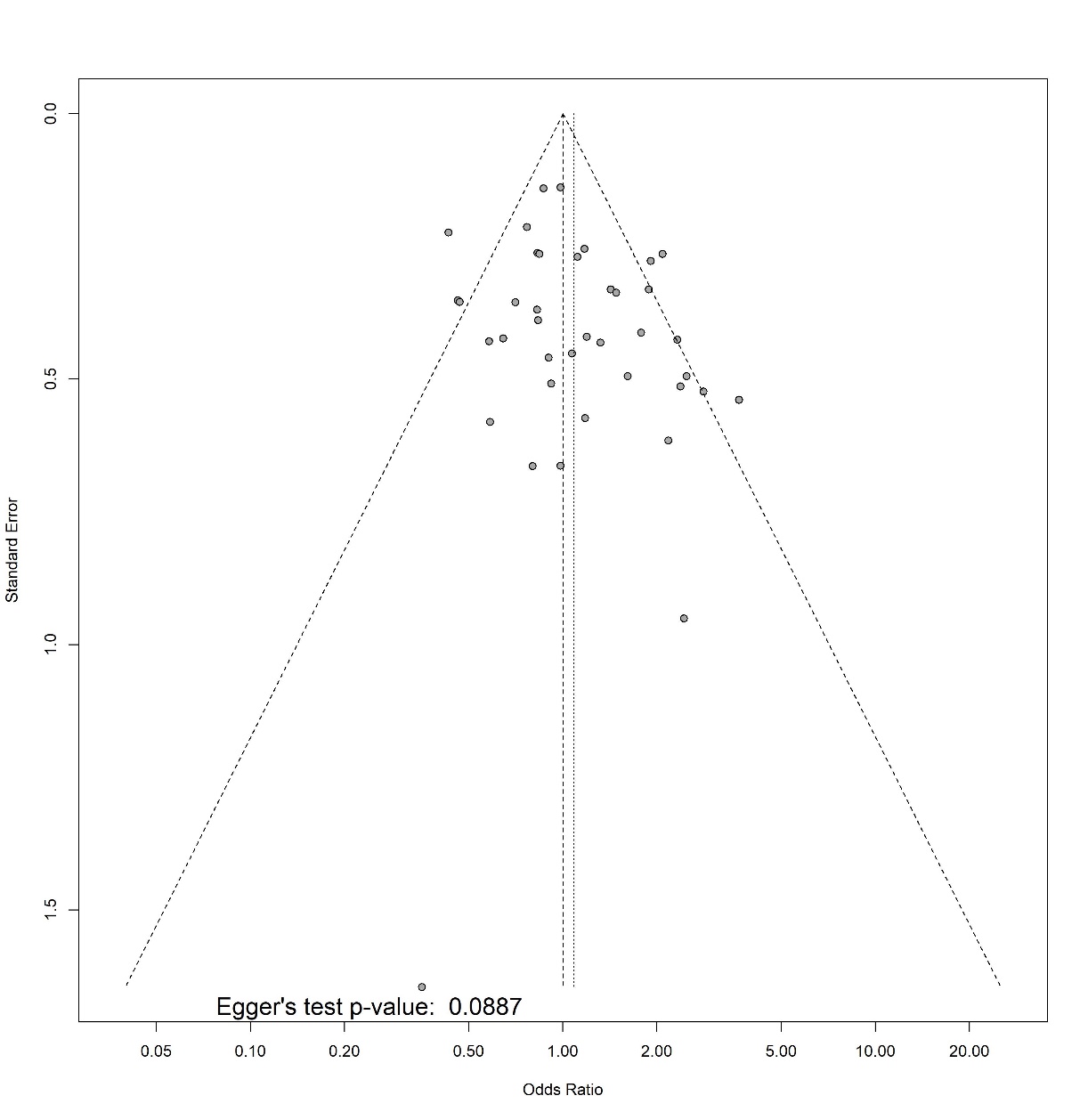

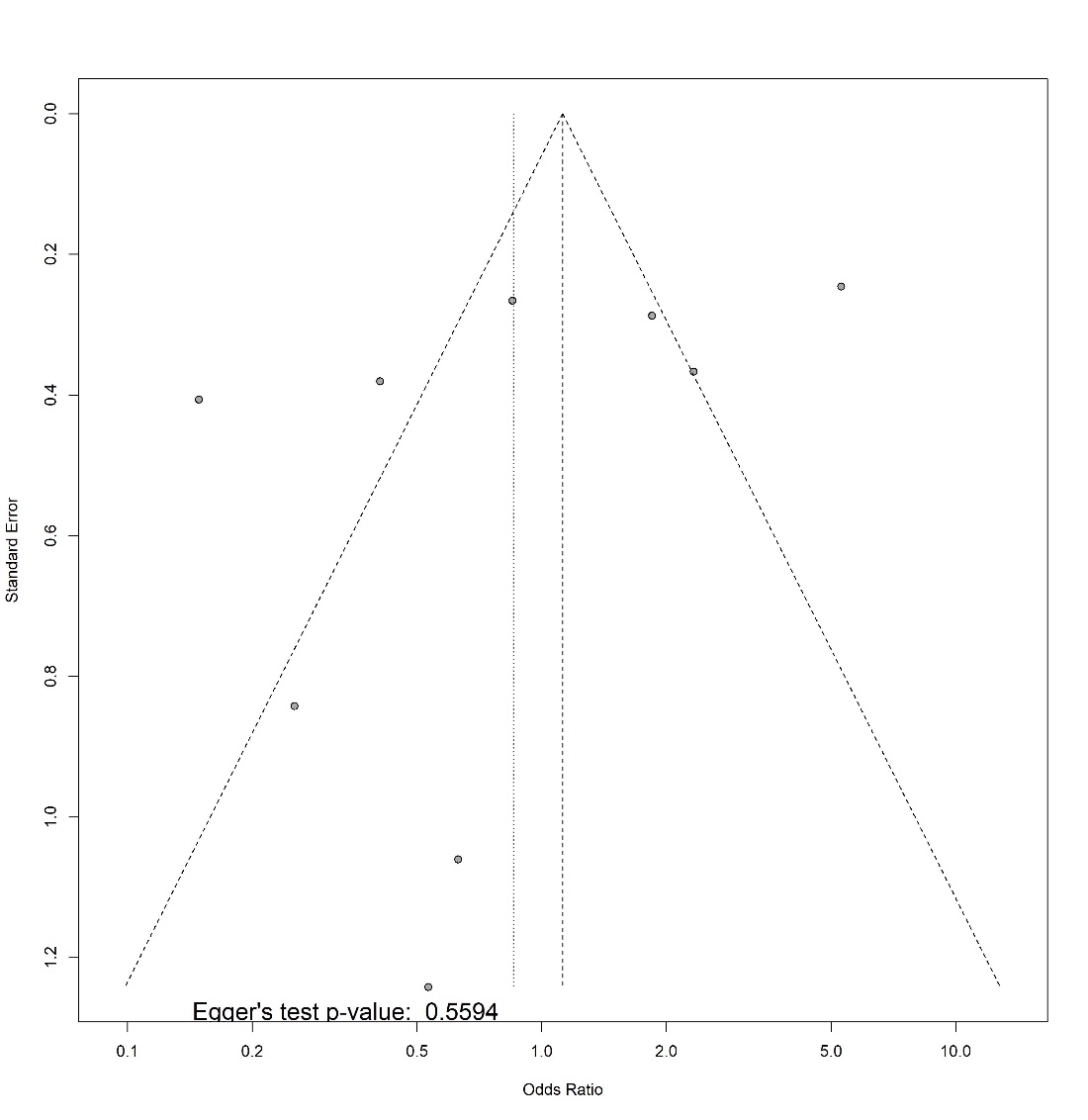


# **Figure S10 Funnel plot for publication bias of association between mental illness and CRKP infections**

# **Figure S11 Funnel plot for publication bias of association between- Diabetes mellitus and CRKP infections**

# **Figure S12 Funnel plot for publication bias of association between- hematological malignancy and CRKP infections**


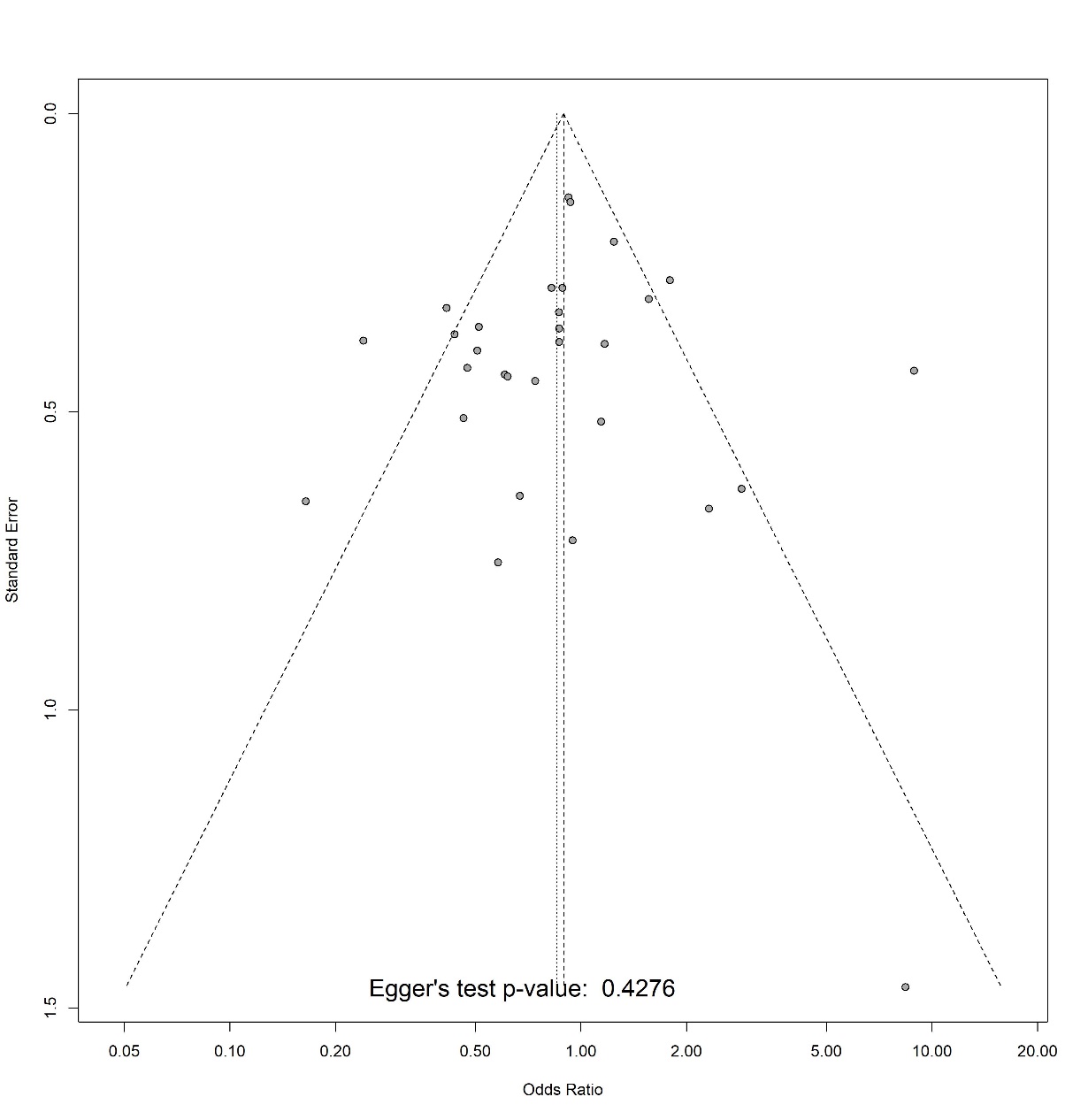


# **Figure S13 Funnel plot for publication bias of association between malignant tumor and CRKP infections**

**
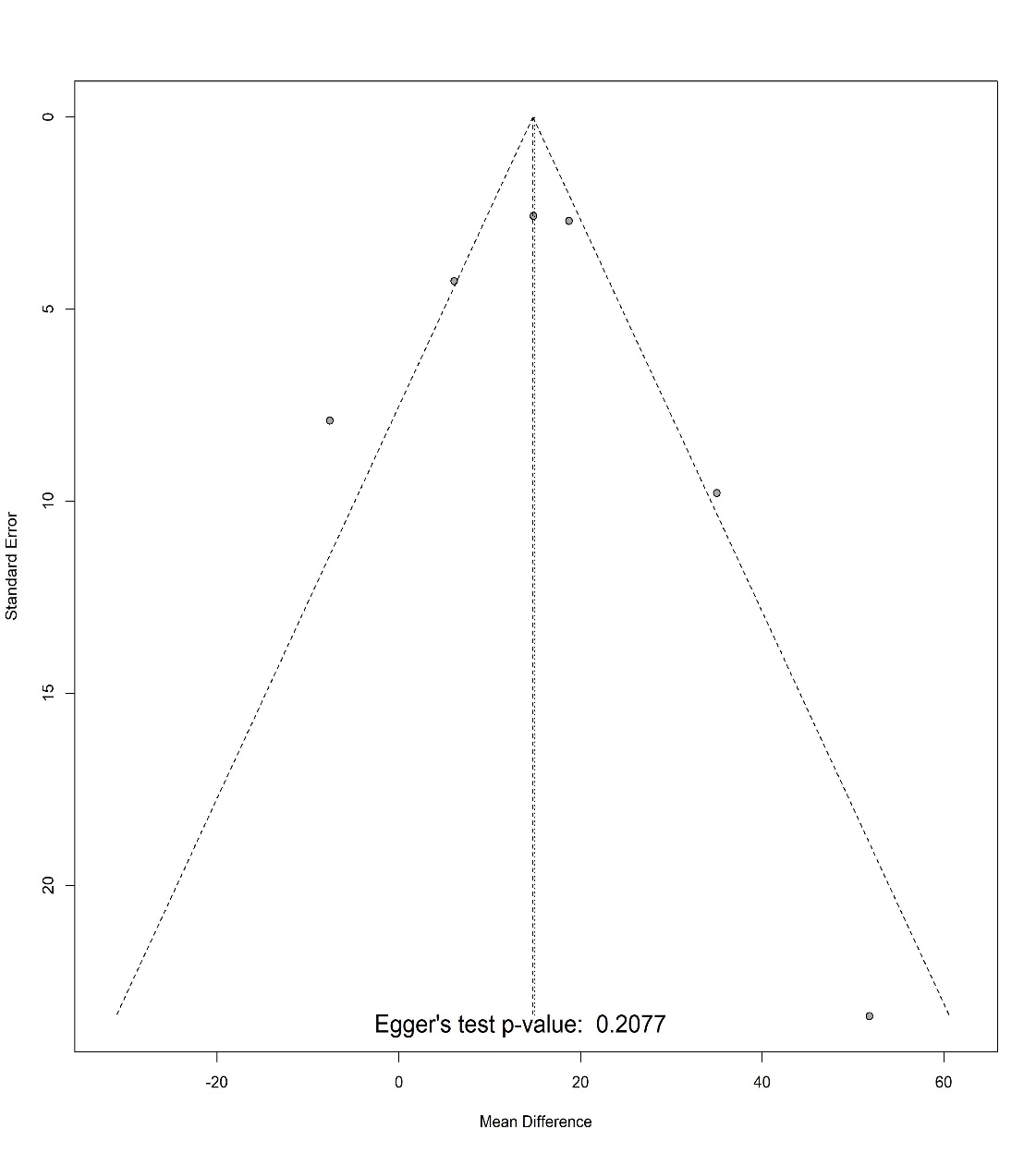
**

# **Figure S14 Funnel plot for publication bias of association between Hospital Stay Before Infection (days) and CRKP infections**


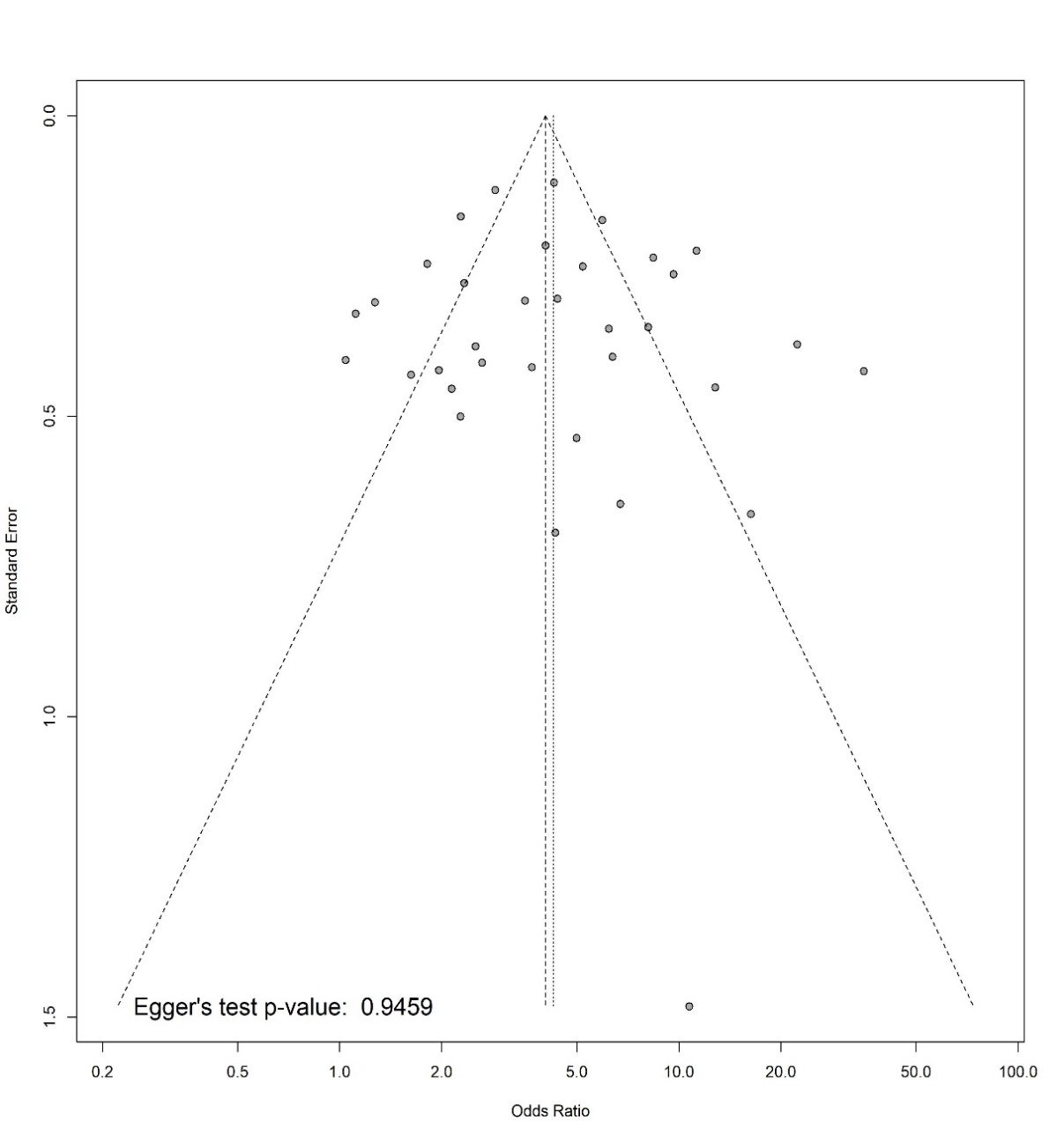


# **Figure S15 Funnel plot for publication bias of association between-ICU admission and CRKP infections**


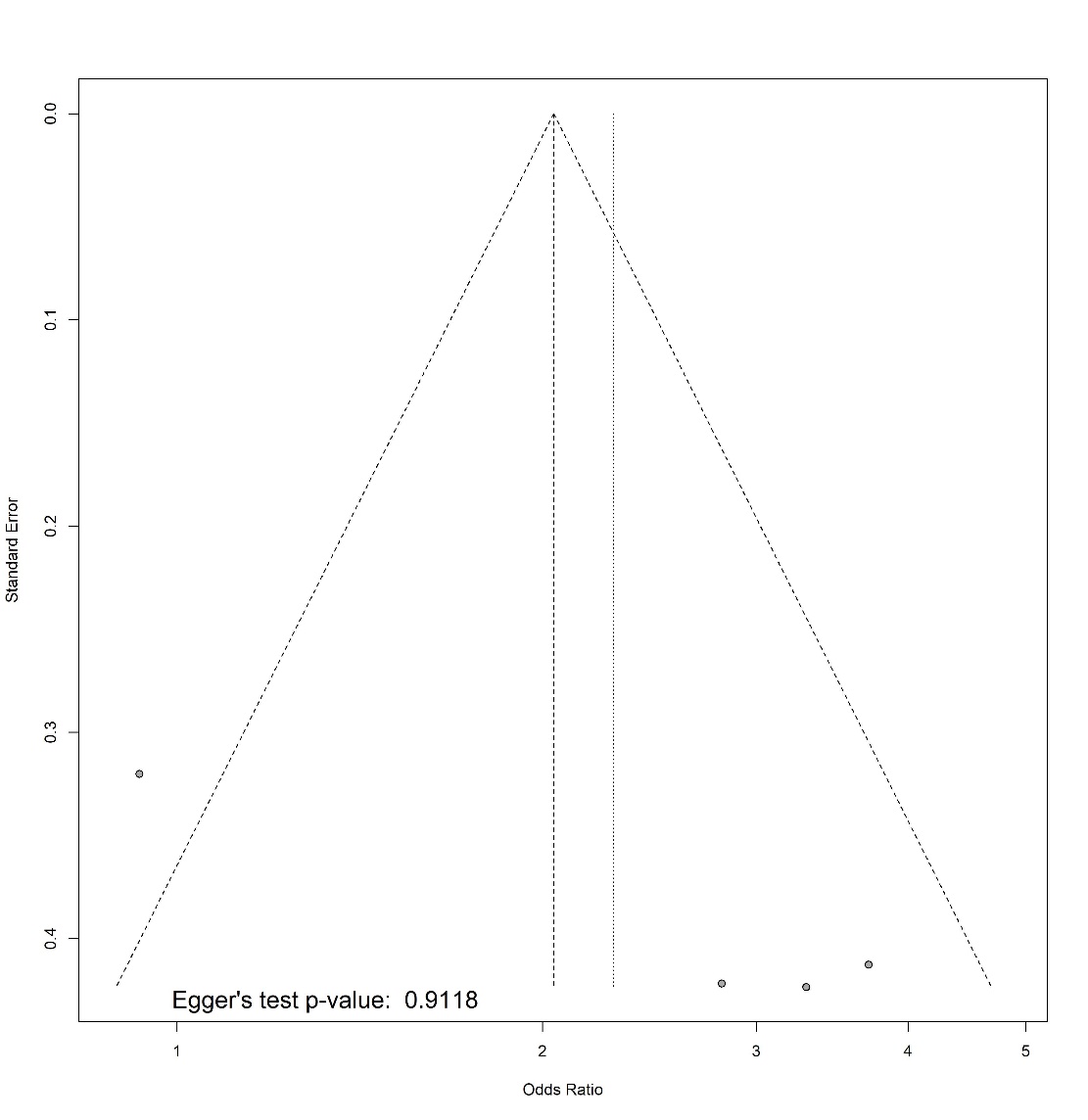

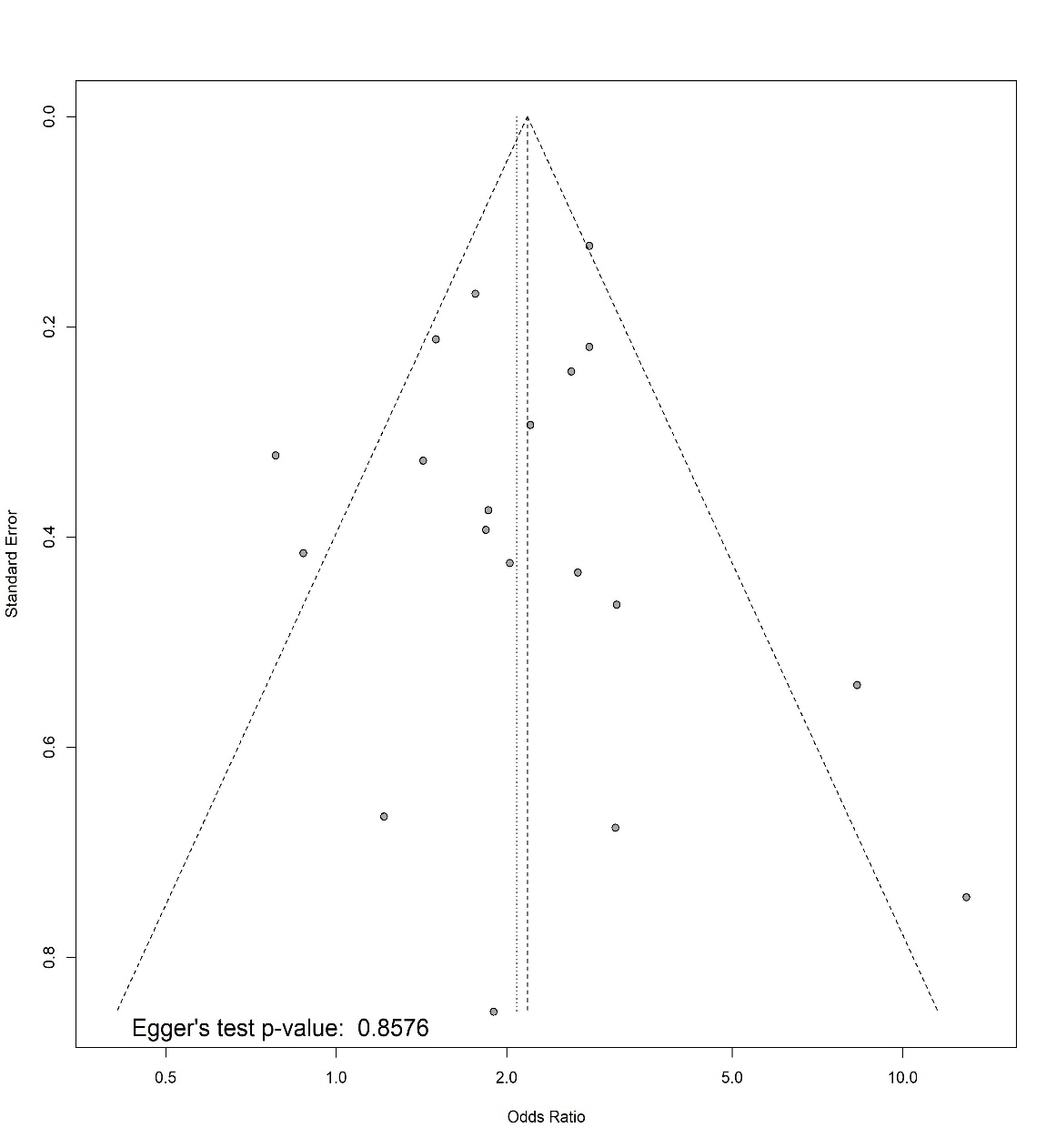


# **Figure S16 Funnel plot for publication bias of association between Transfer from other hospital and CRKP infections**

# **Figure S17 Funnel plot for publication bias of association between Prior hospitalization (within 12 months) and CRKP infections**


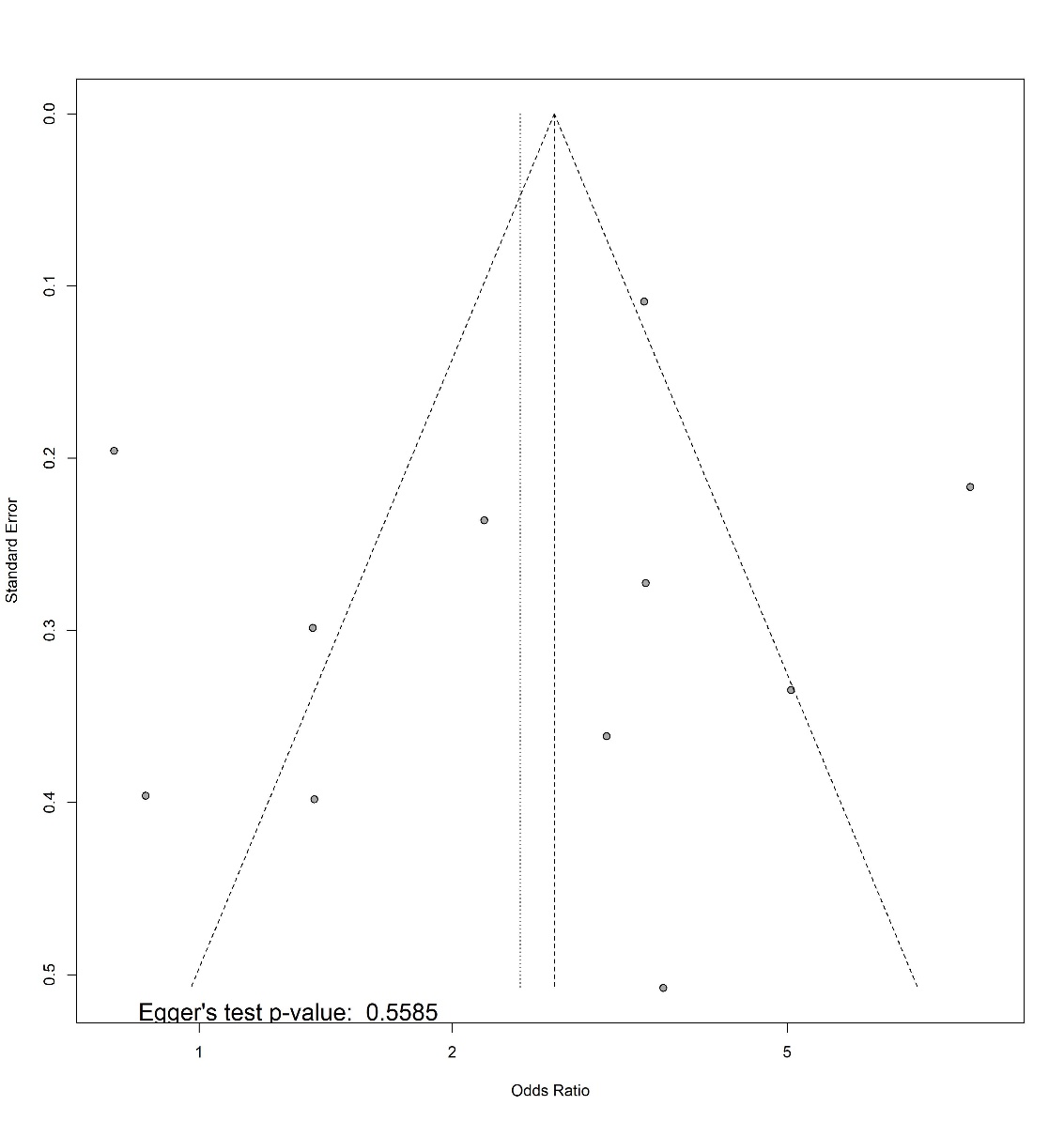


# **Figure S18 Funnel plot for publication bias of association between- Nasogastric catheter and CRKP infections**


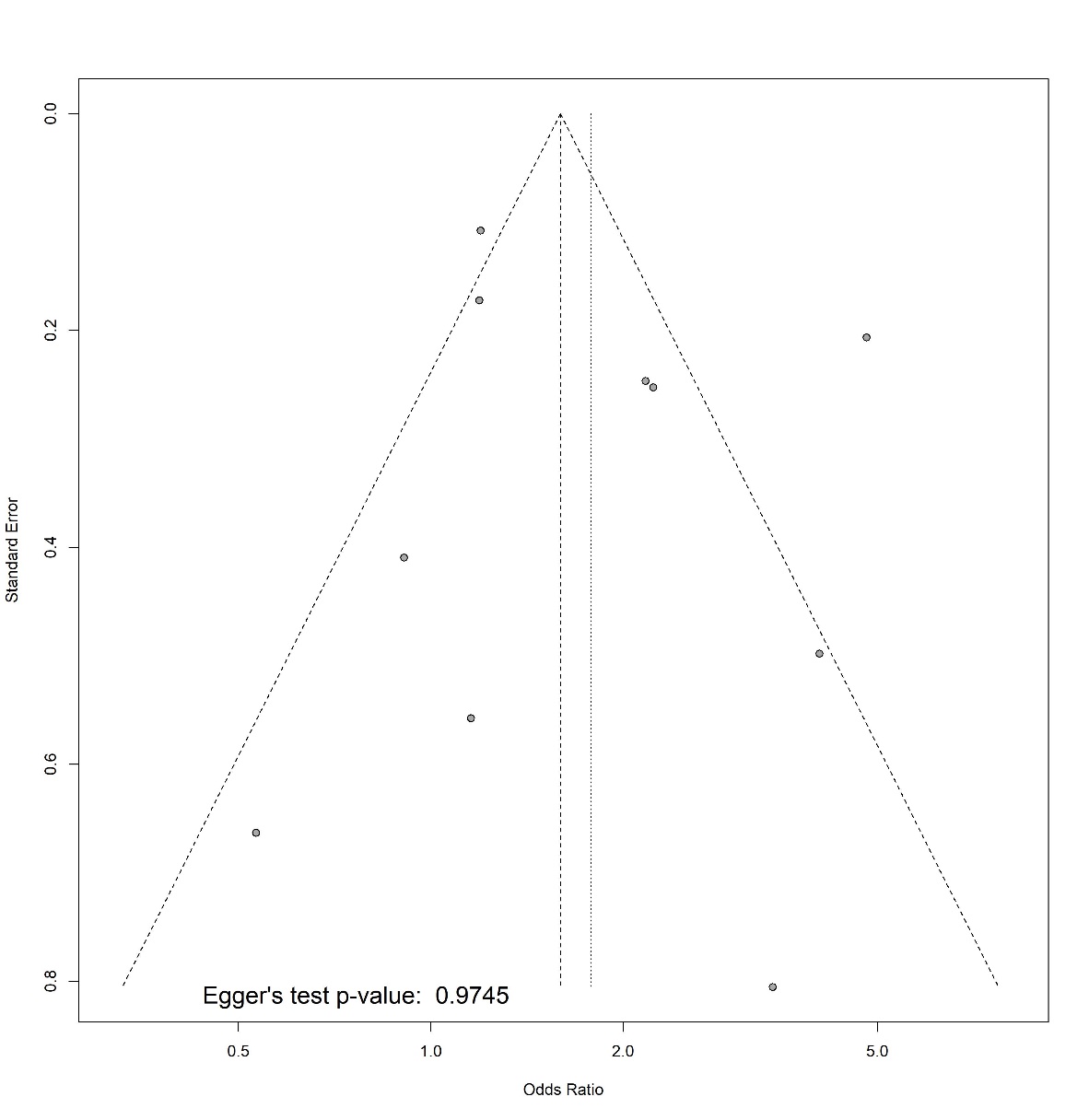


# **Figure S19 Funnel plot for publication bias of association between- Parenteral nutrition and CRKP infections**


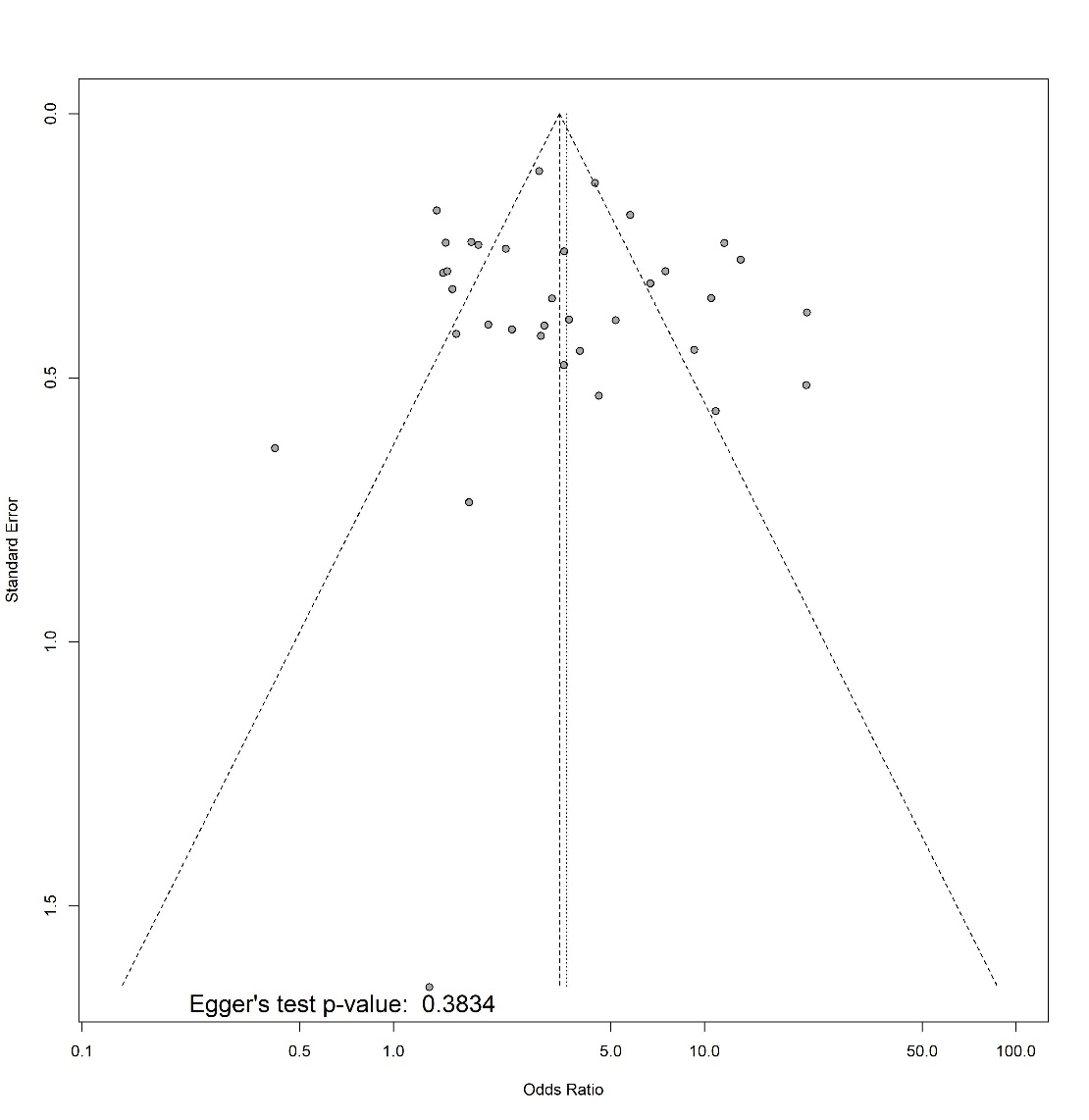


# **Figure S20 Funnel plot for publication bias of association between Mechanical ventilation and CRKP infections**


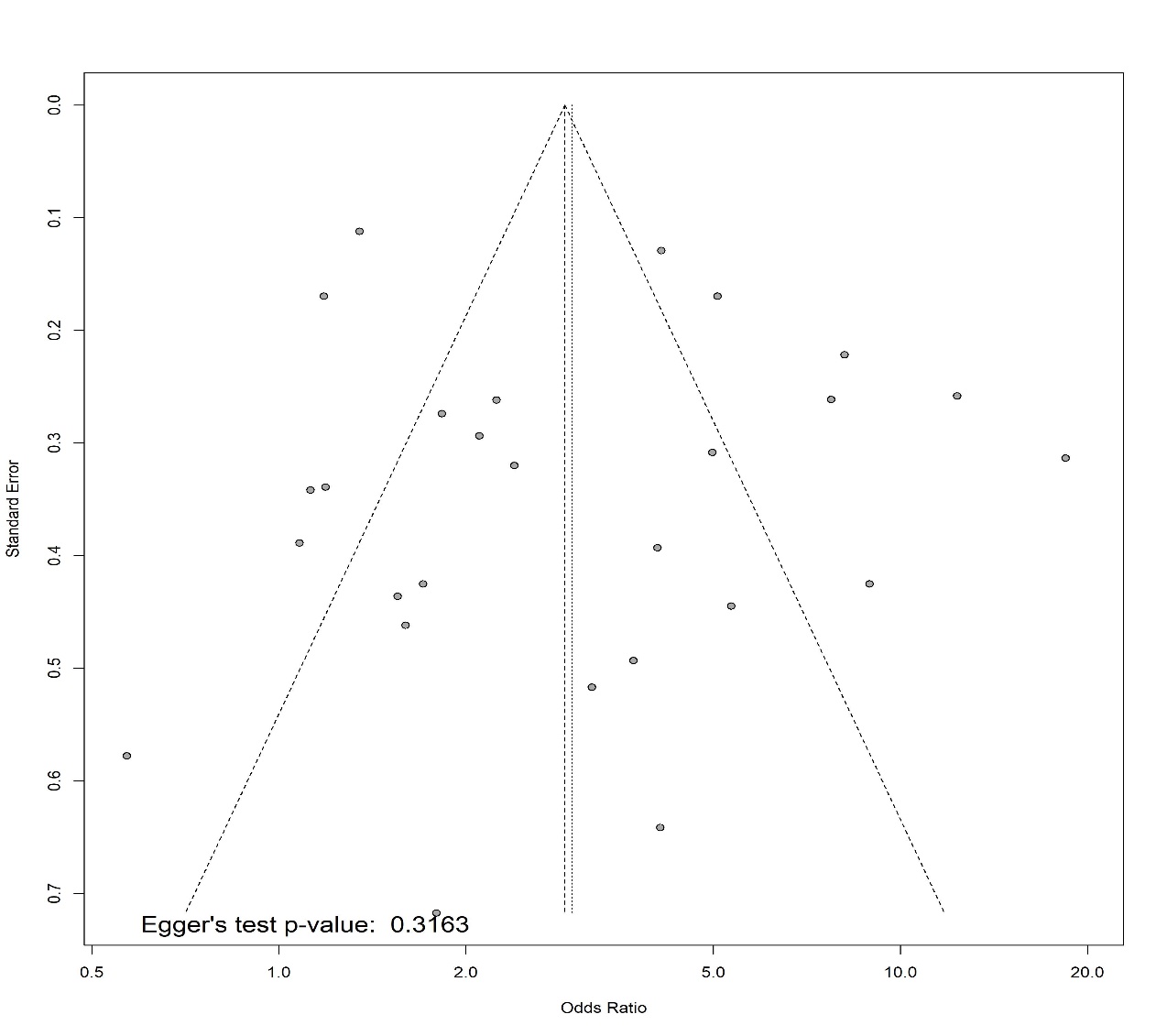


# **Figure S21 Funnel plot for publication bias of association between- Indwelling urinary catheter and CRKP infections**


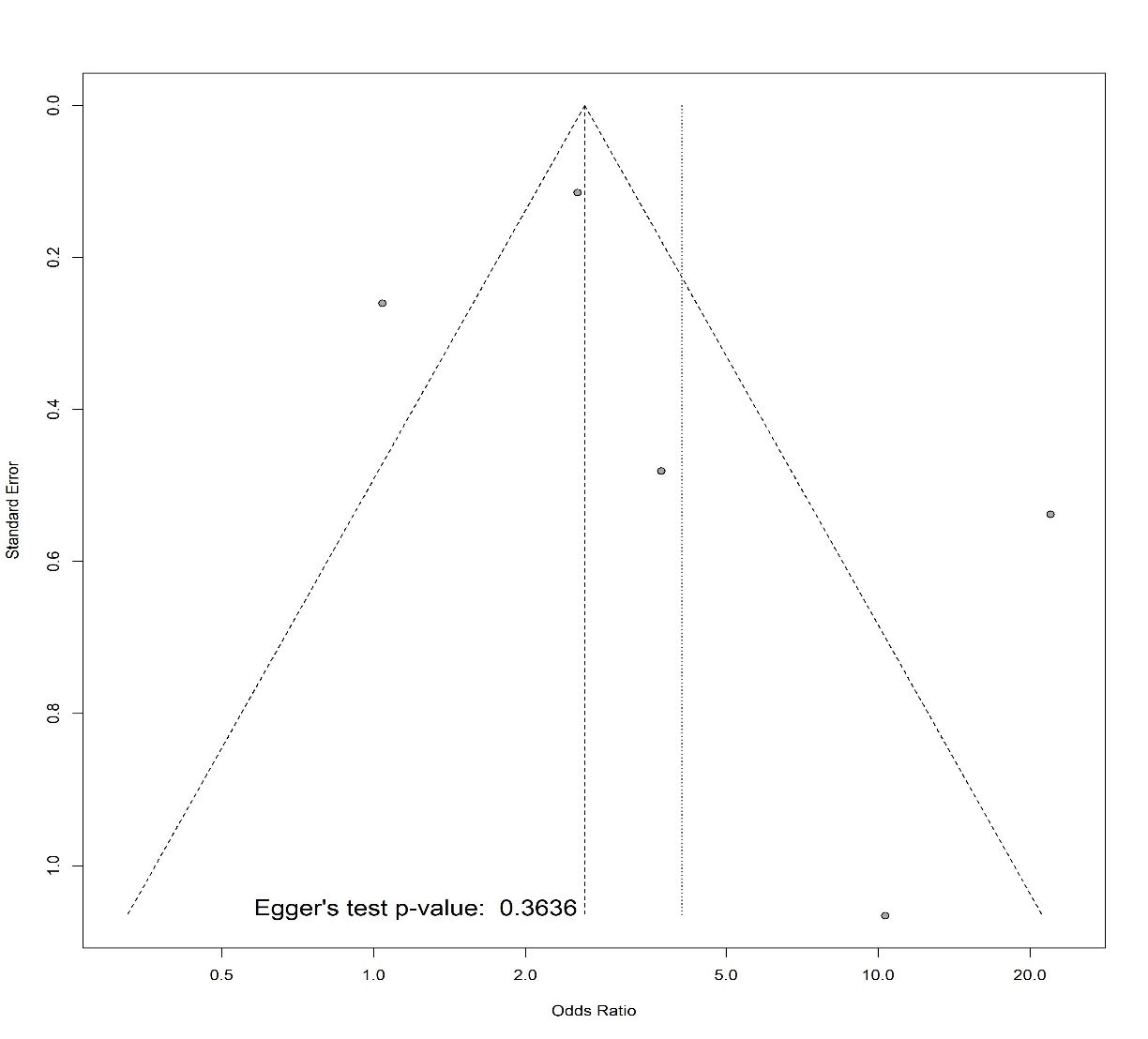


# **Figure S22 Funnel plot for publication bias of association between Endoscopy and CRKP infections**


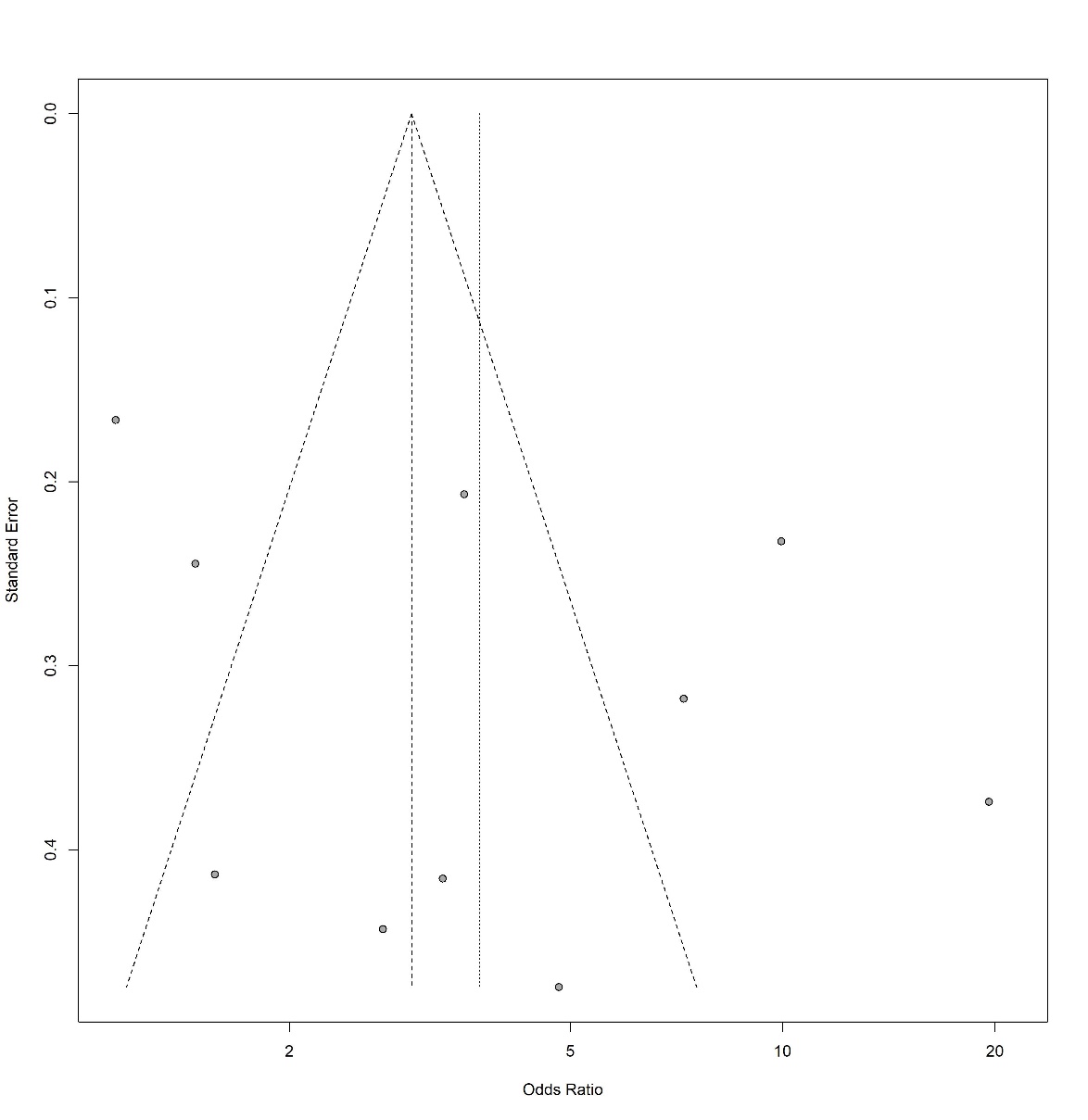


# **Figure S23 Funnel plot for publication bias of association between Tracheal cannula and CRKP infections**


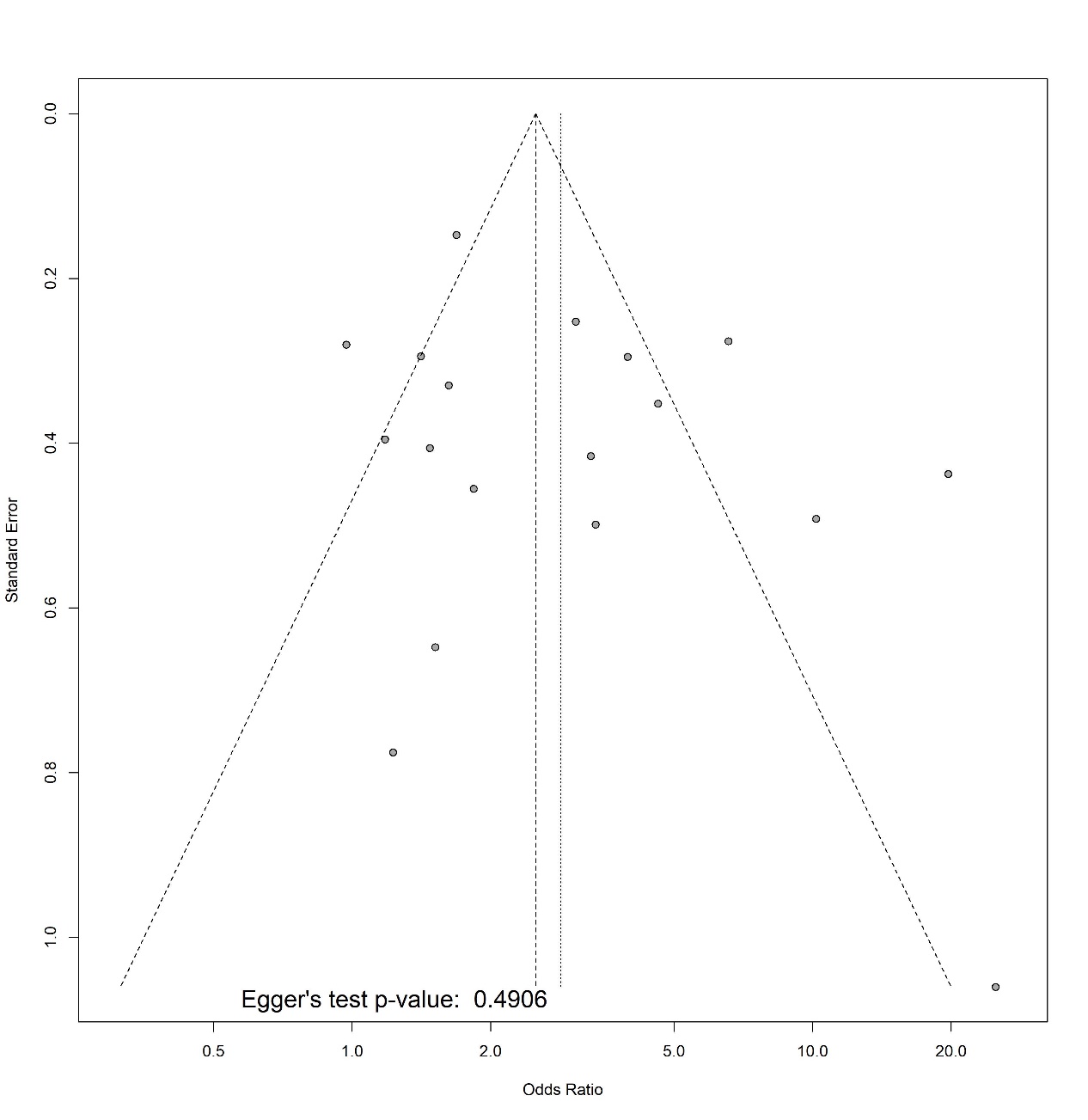


# **Figure S24 Funnel plot for publication bias of association between Tracheostomy and CRKP infections**


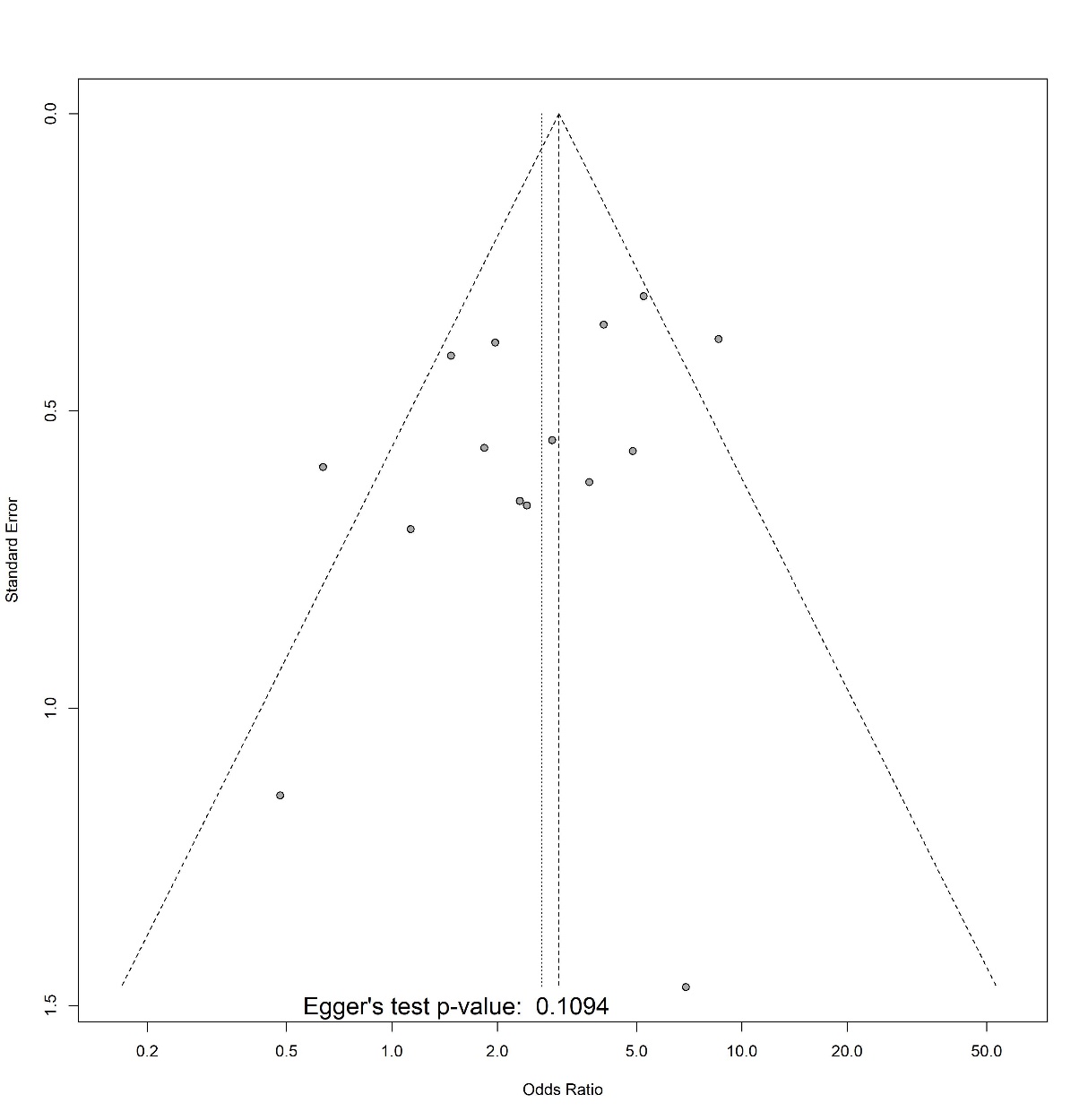


# **Figure S25 Funnel plot for publication bias of association between Dialysis and CRKP infections**


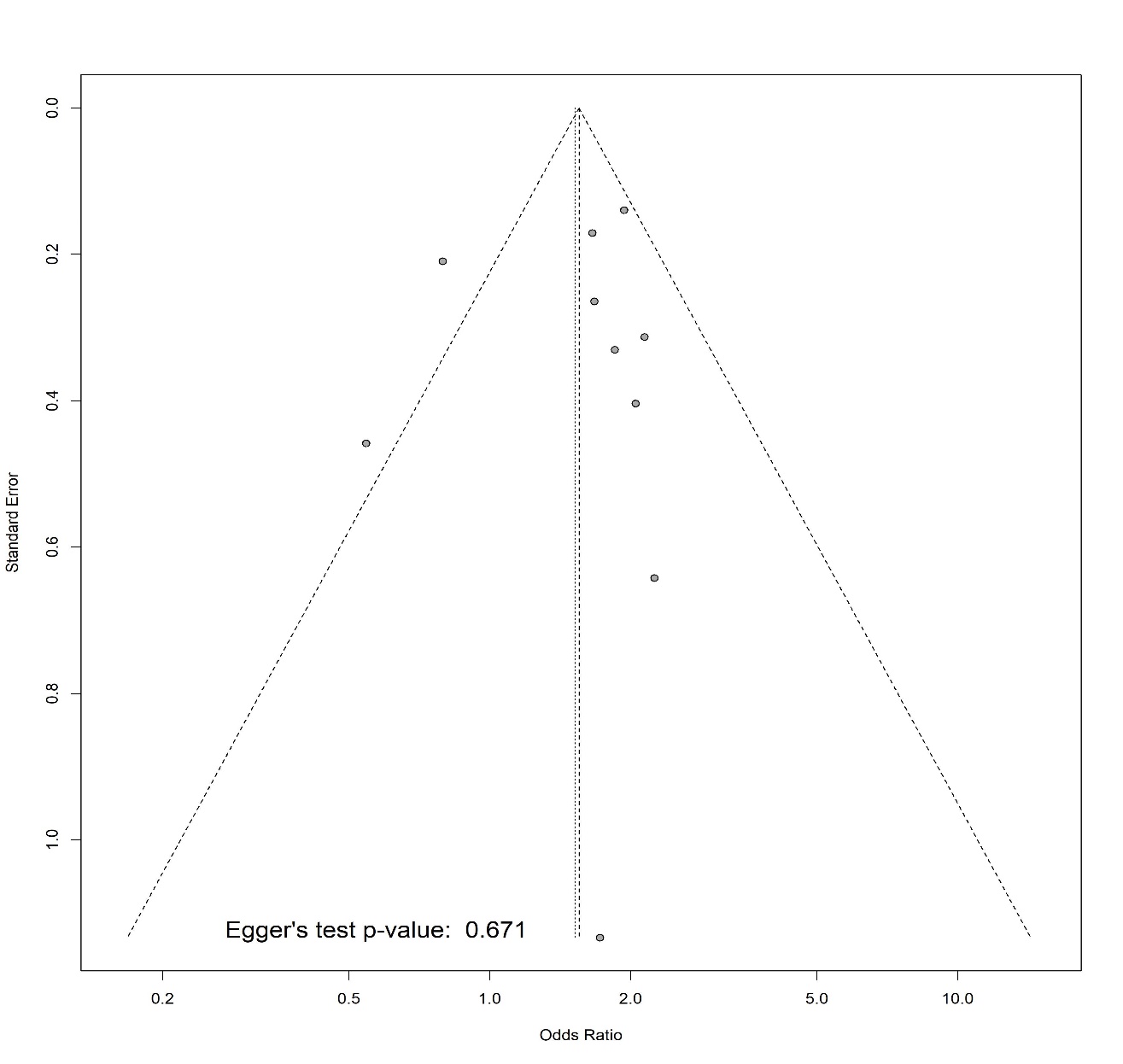


# **Figure S26 Funnel plot for publication bias of association between Surgical drainage and CRKP infections**


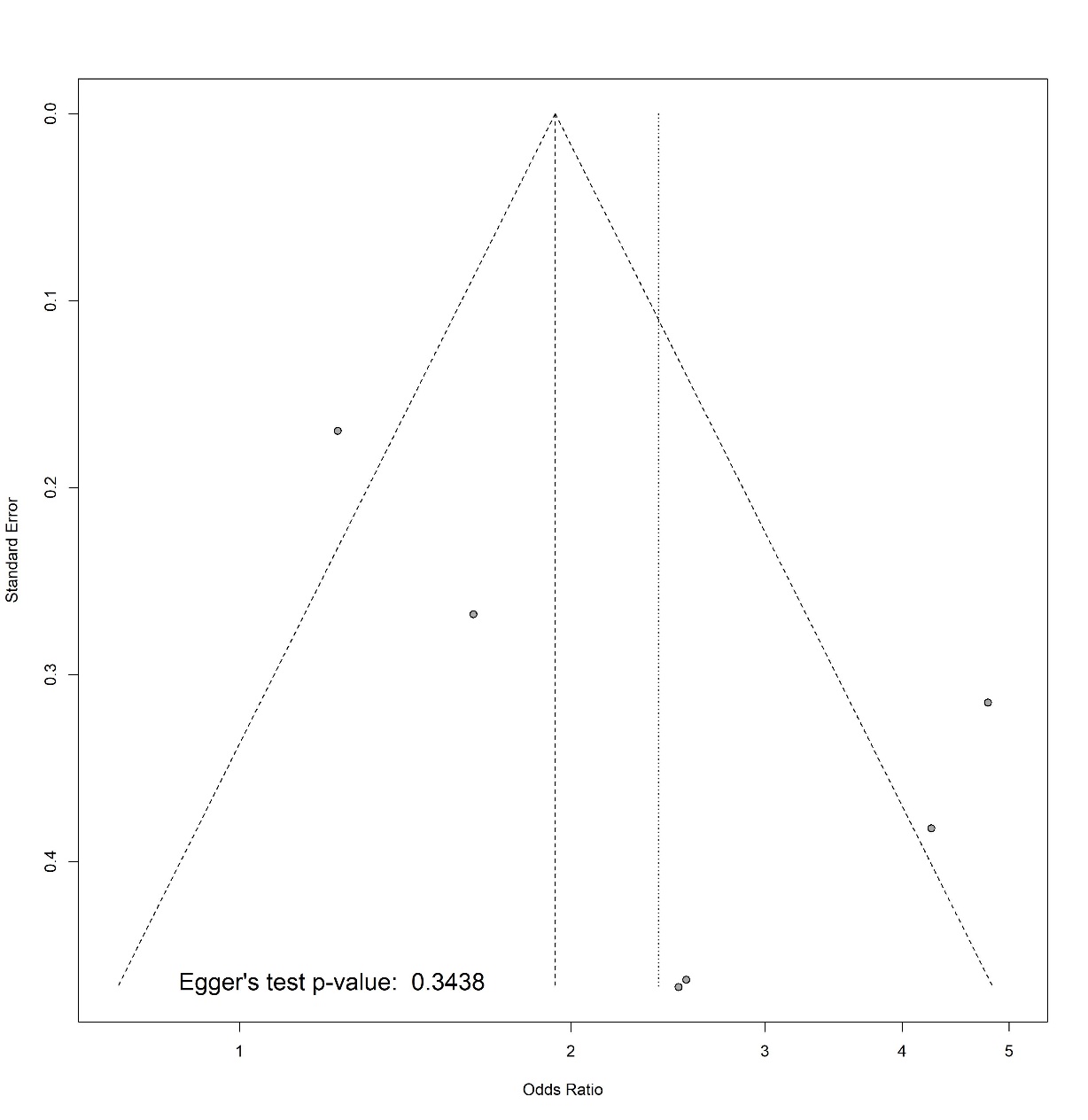


# **Figure S27 Funnel plot for publication bias of association between Peripheral Catheter and CRKP infections**


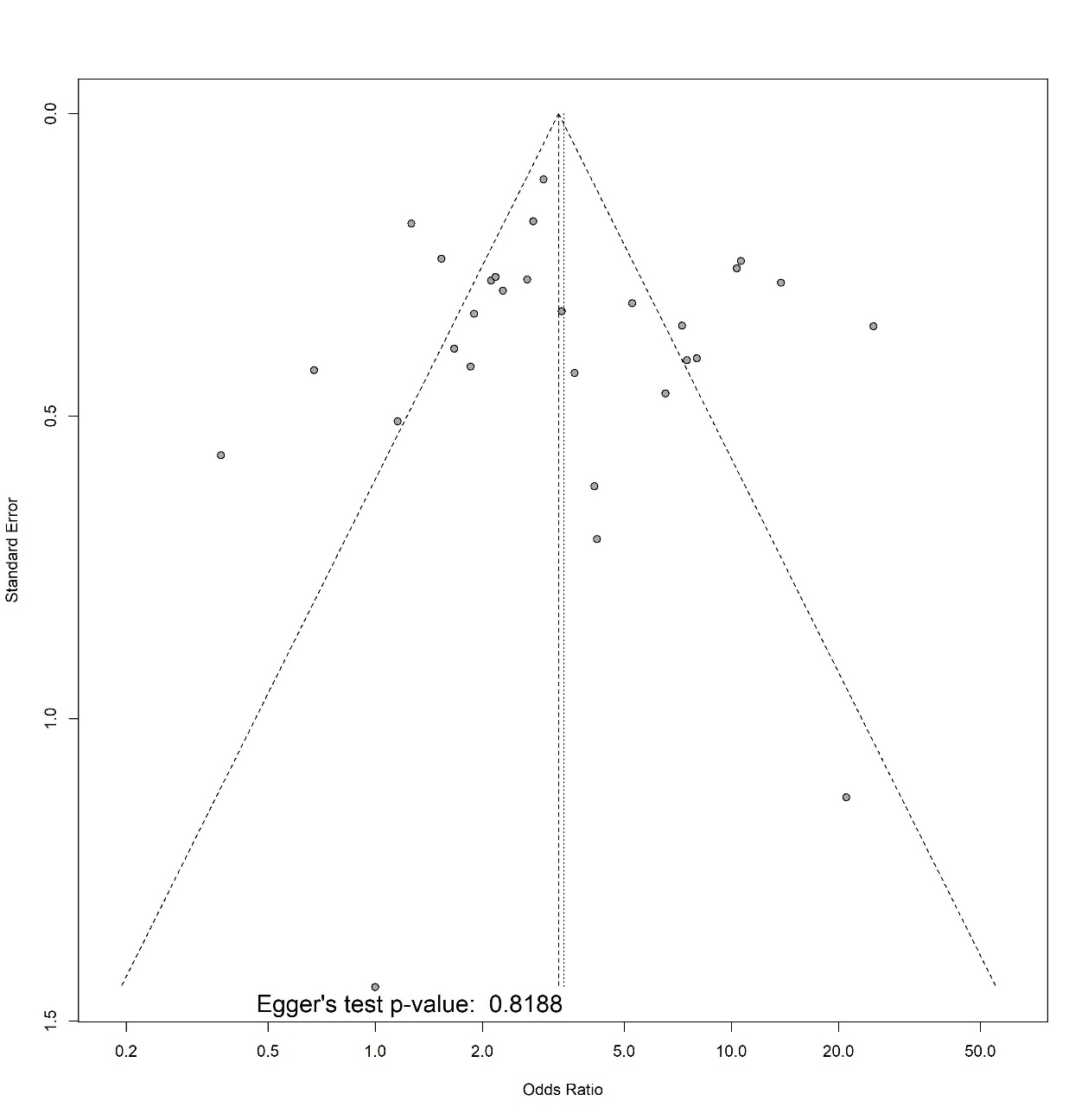


# **Figure S28 Funnel plot for publication bias of association between Central venous catheter and CRKP infections**


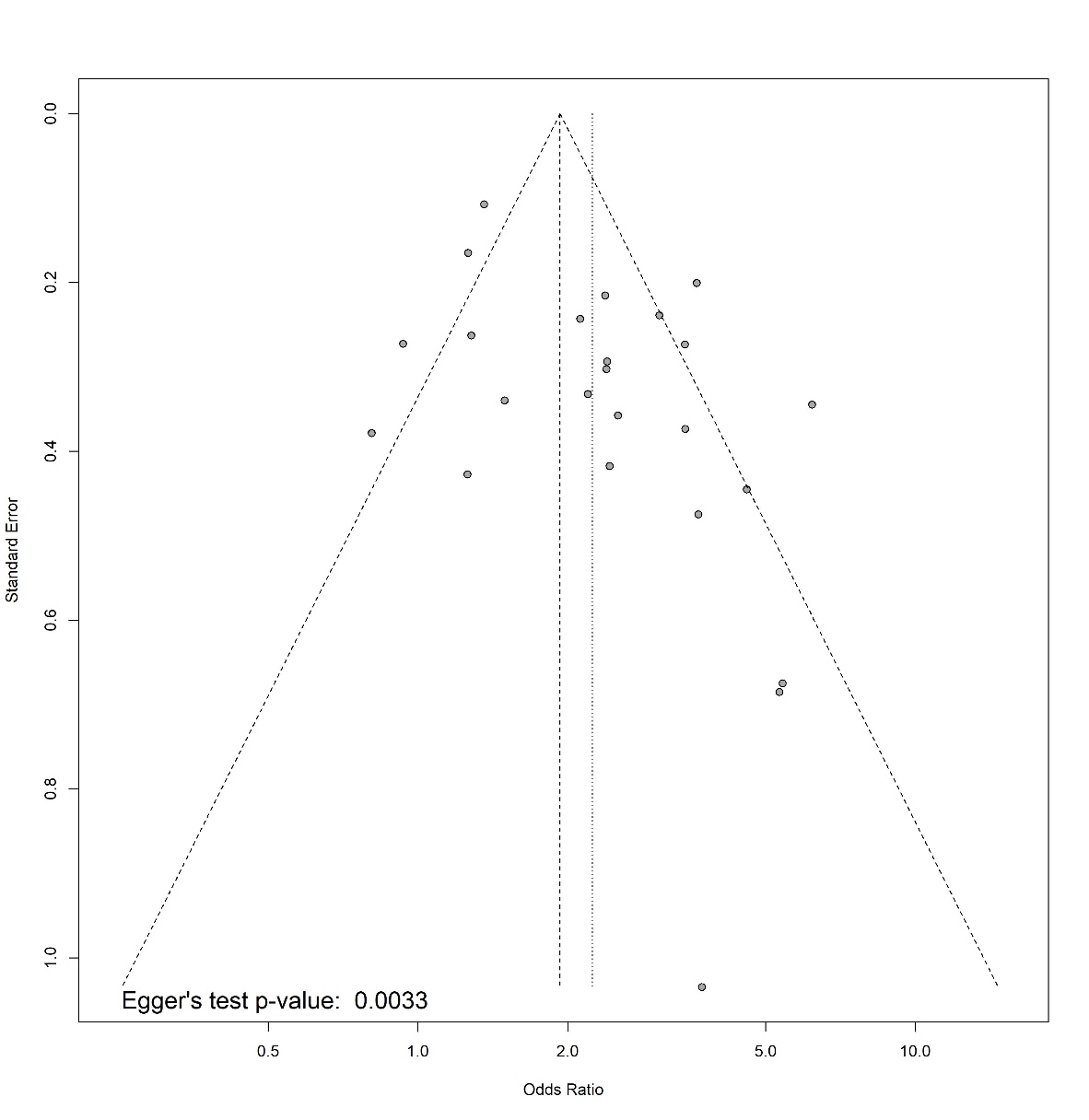


# **Figure S29 Funnel plot for publication bias of association betweenβ-lactamase inhibitor and CRKP infections**


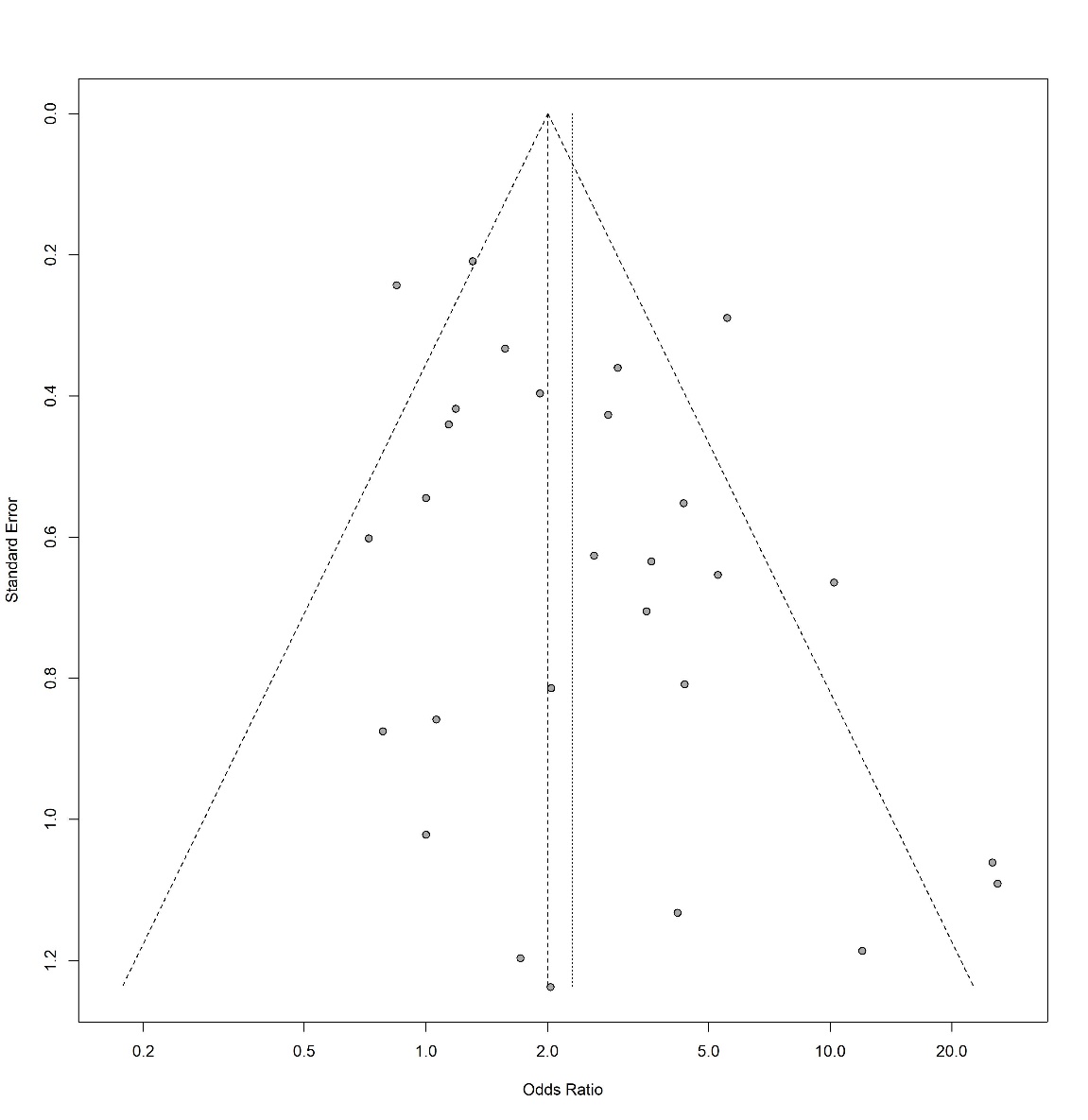


# **Figure S30 Funnel plot for publication bias of association between Aminoglycoside and CRKP infections**


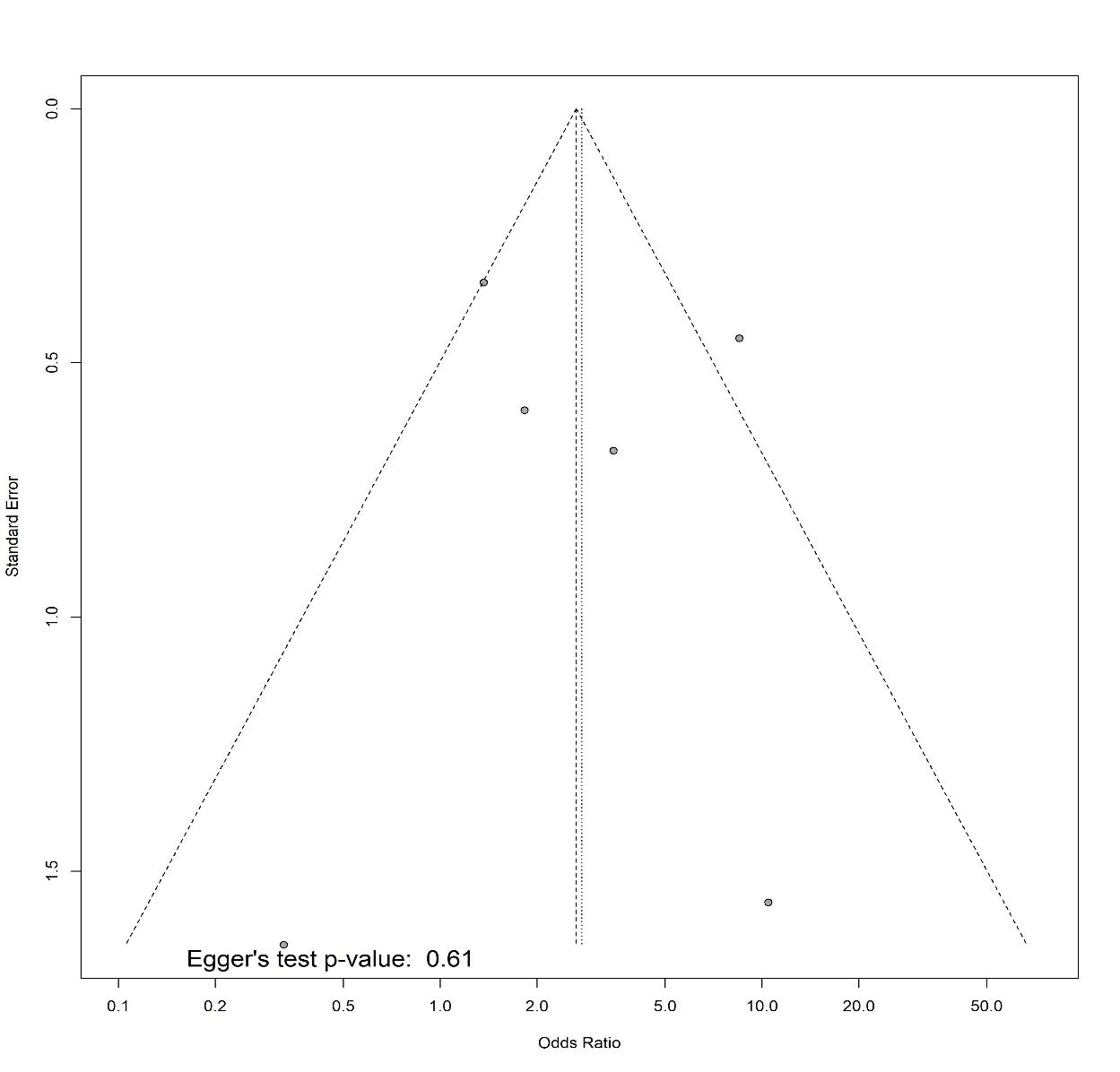


# **Figure S31 Funnel plot for publication bias of association between Macrolides and CRKP infections**


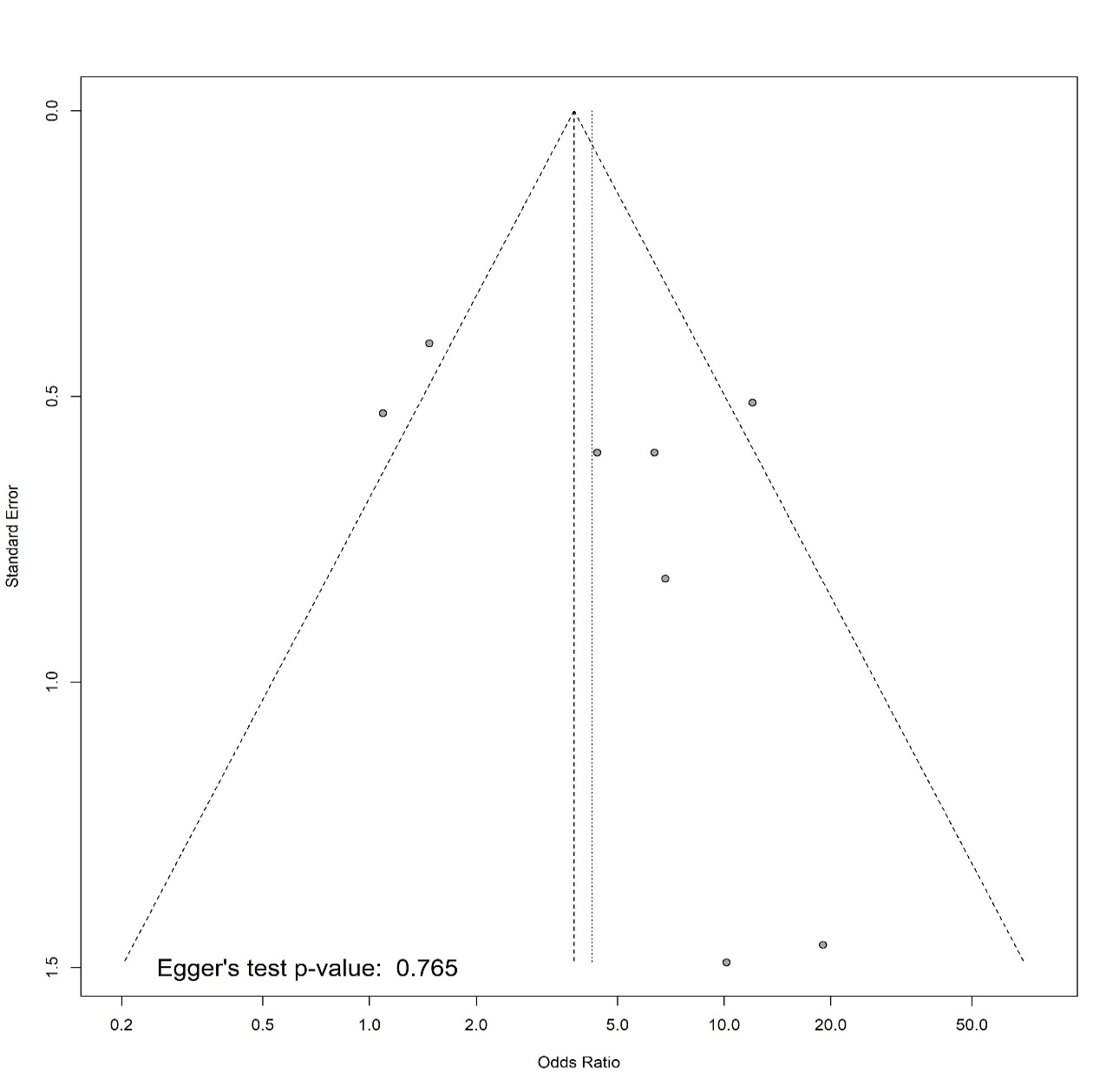


# **Figure S32 Funnel plot for publication bias of association between Polymyxin and CRKP infections**


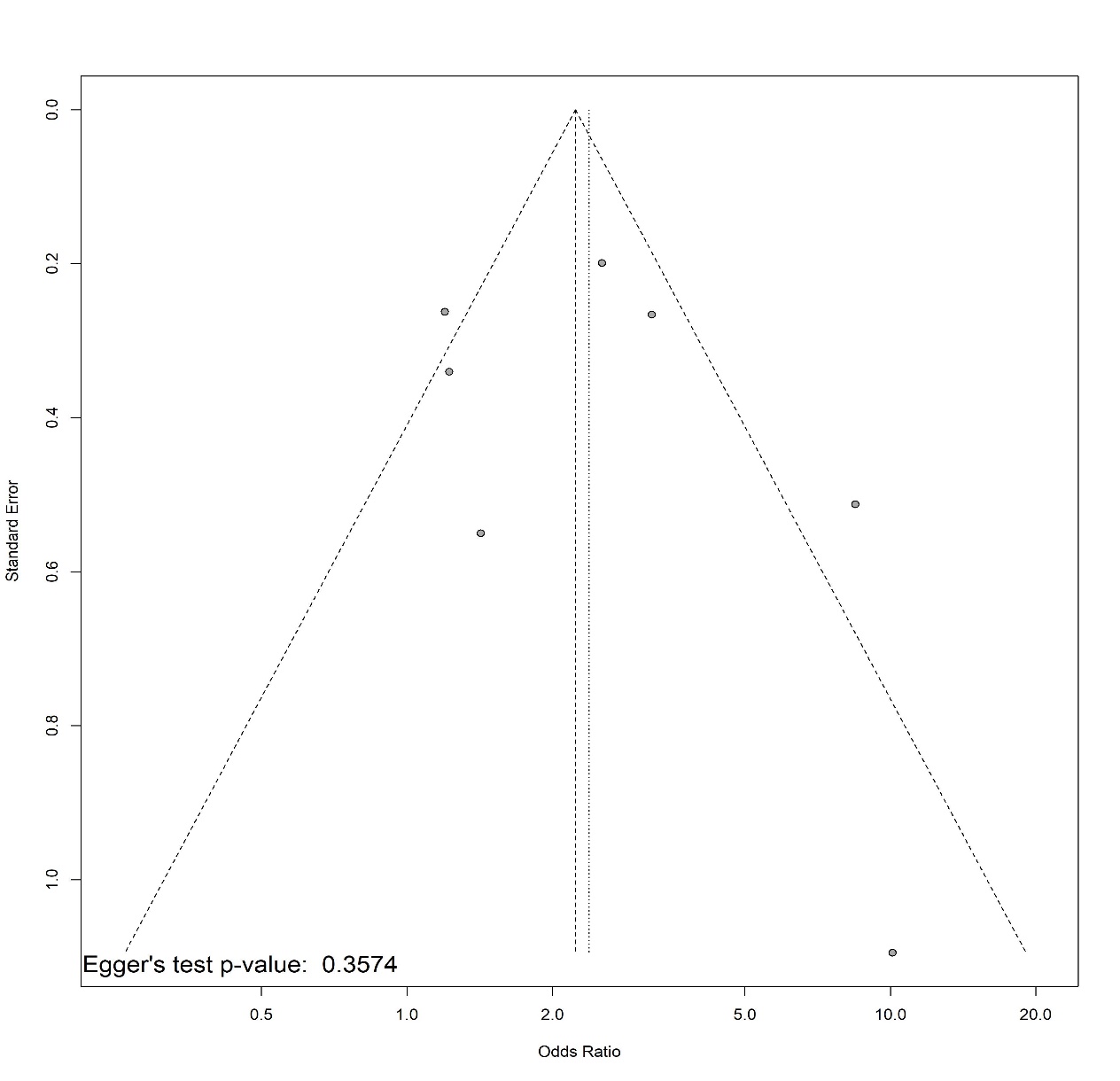


# **Figure S33 Funnel plot for publication bias of association between Linezolid and CRKP infections**


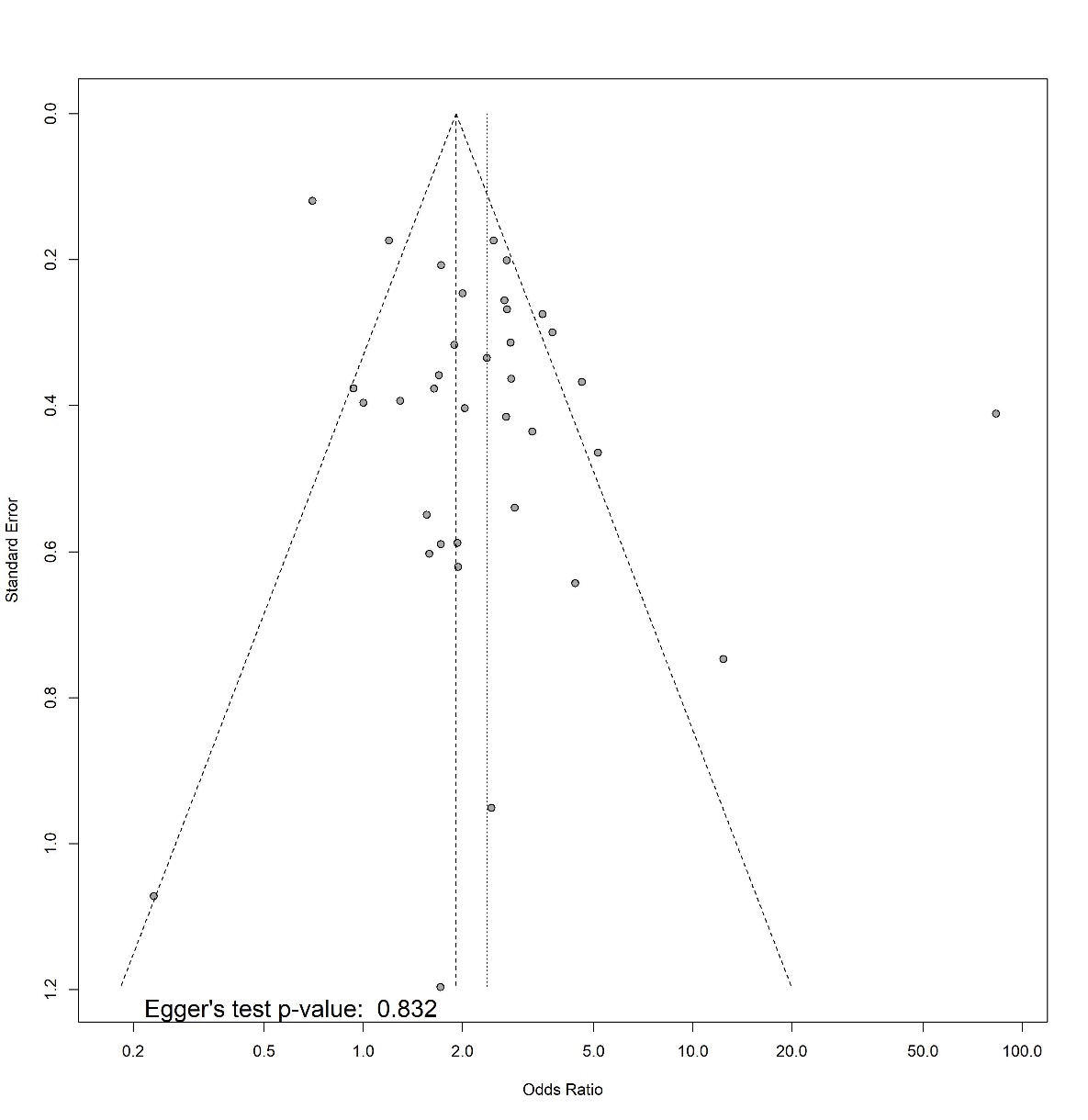

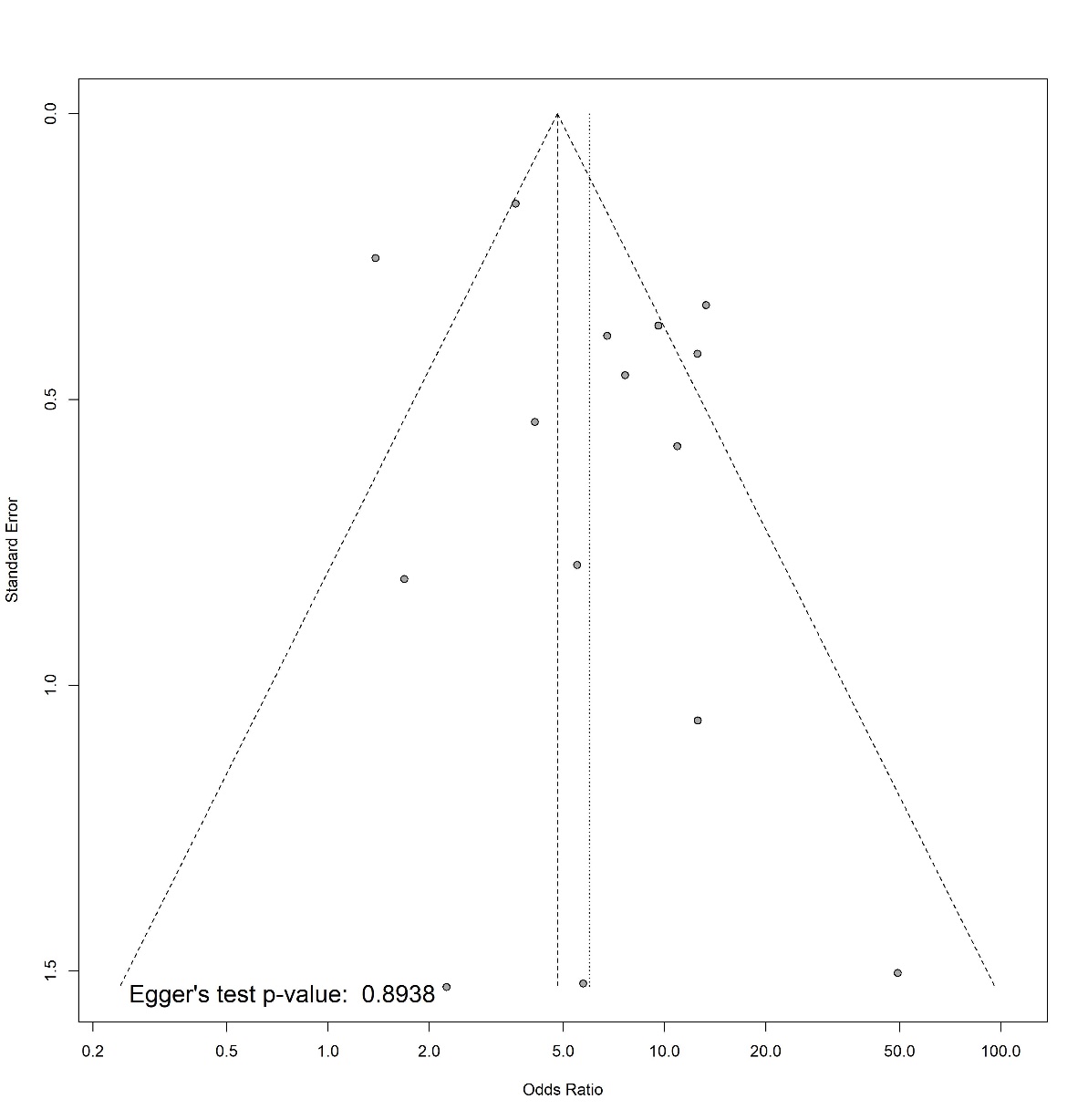


Figure S35 funnel plot for publication bias of association between Fluoroquinolone and CRKP infections

# **Figure S35 Funnel plot for publication bias of association between Glycopeptides and CRKP infections**


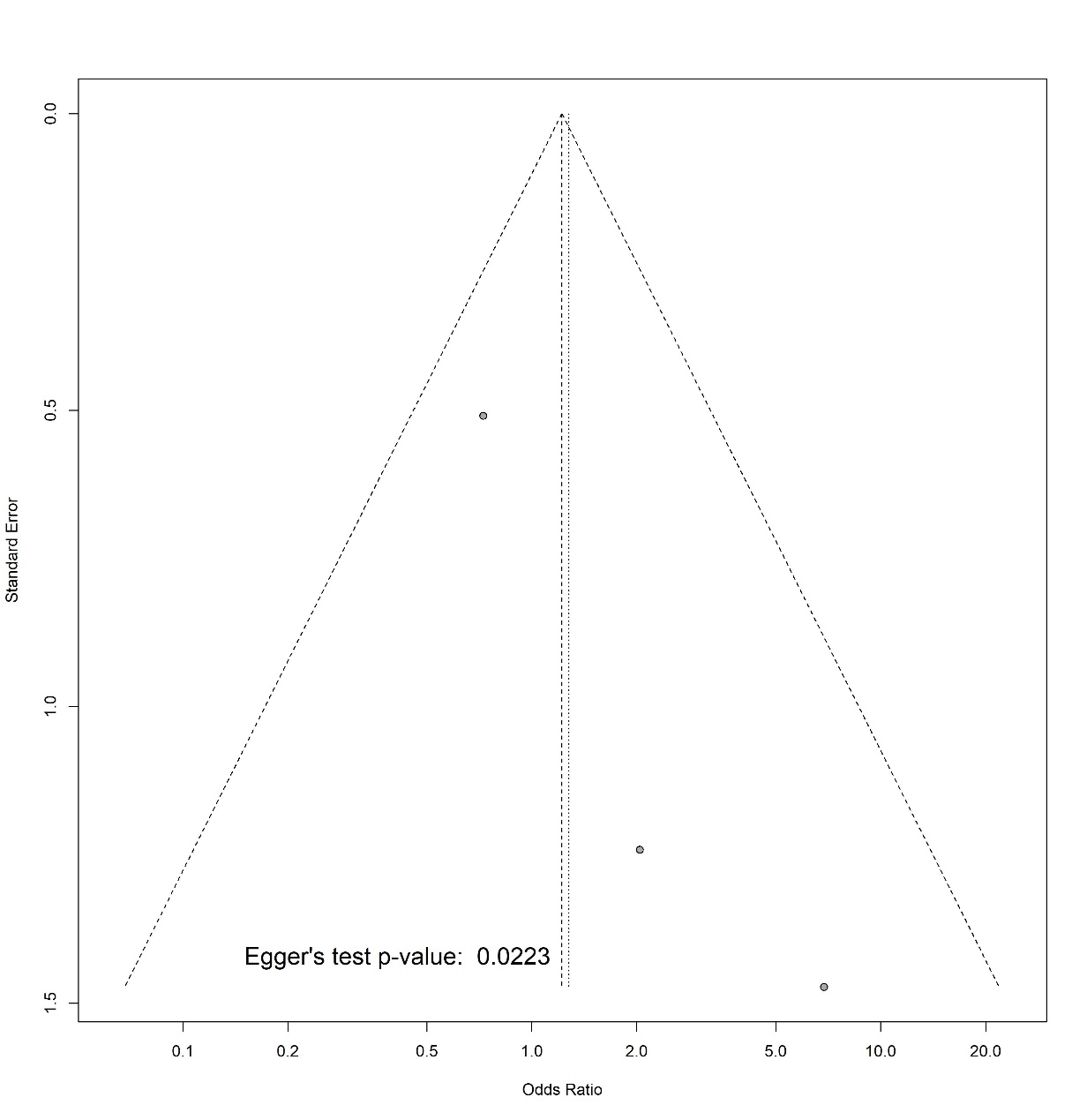


# **Figure S36 Funnel plot for publication bias of association between Sulfanilamides and CRKP infections**


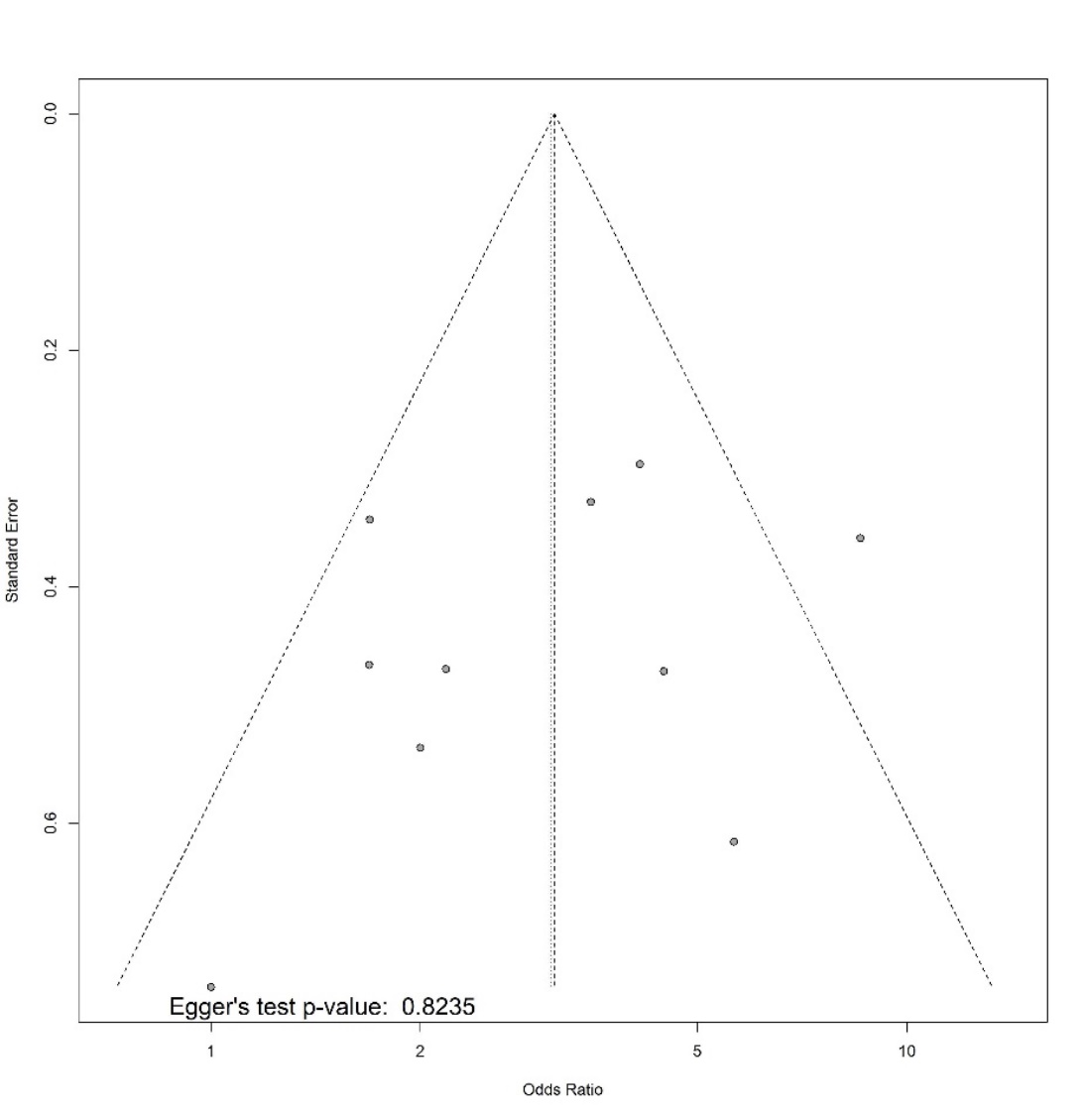


# **Figure S37 Funnel plot for publication bias of association between Antifungal agents and CRKP infections**


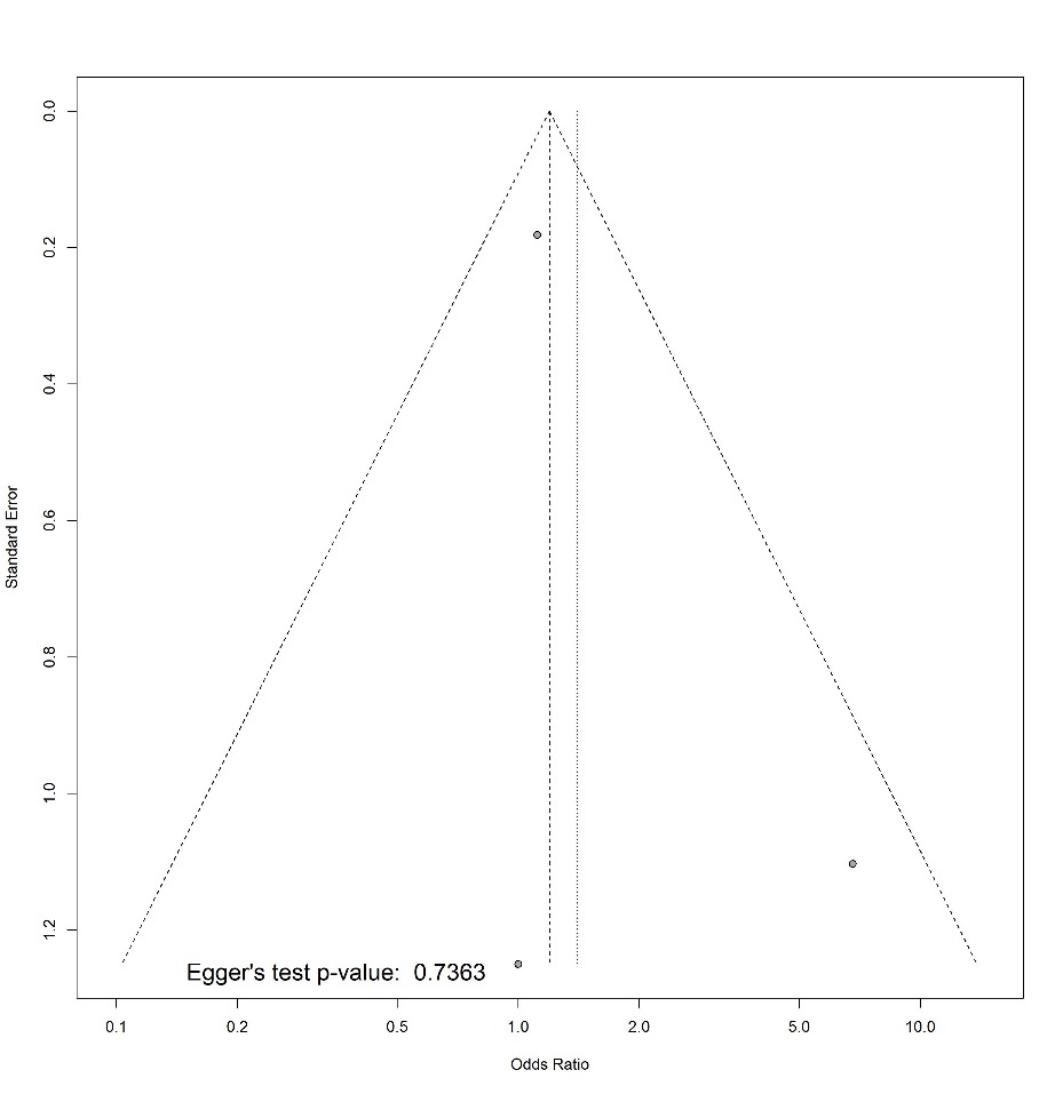


# **Figure S38 Funnel plot for publication bias of association between Clindamycin and CRKP infections**


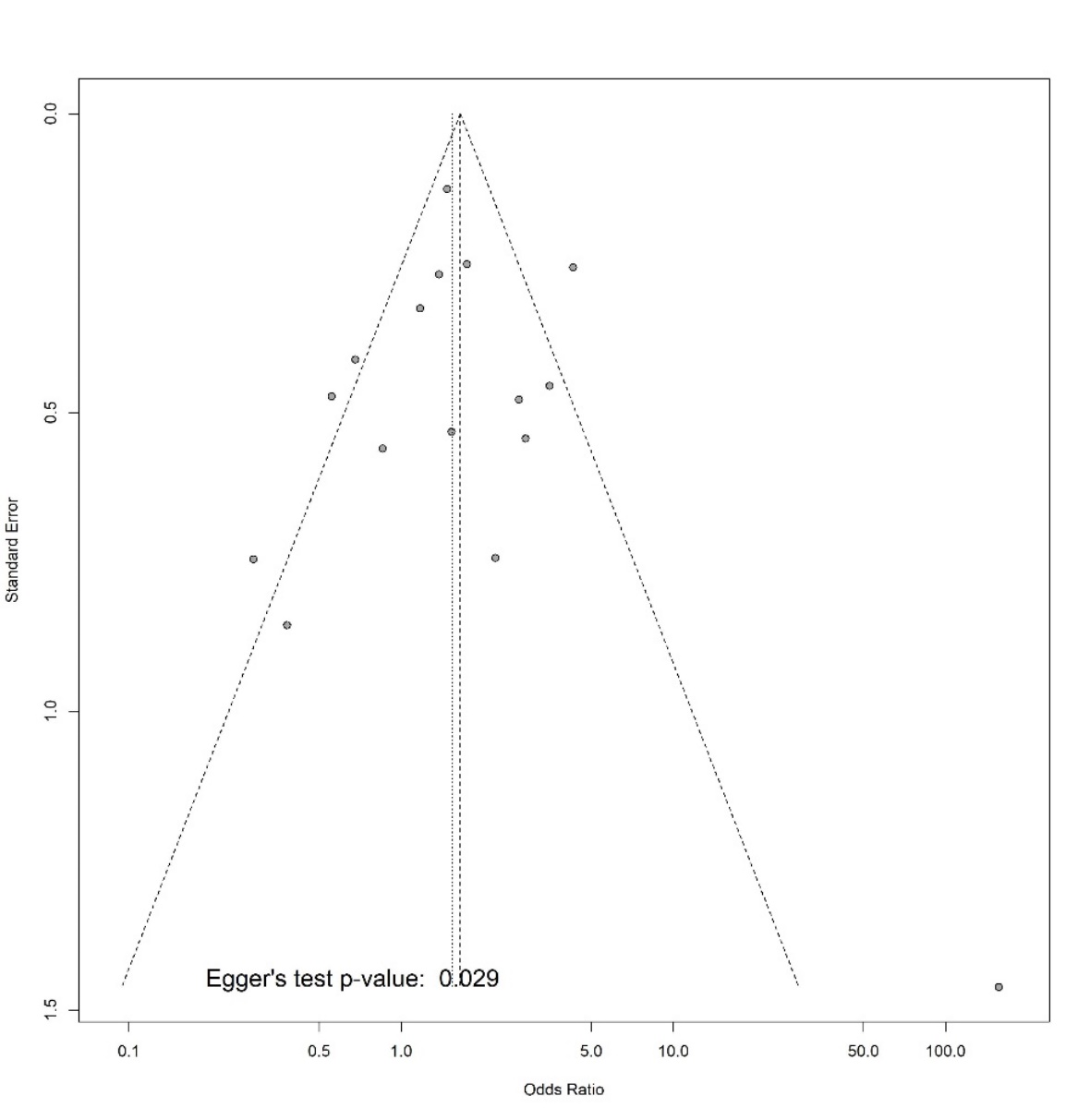


# **Figure S39 Funnel plot for publication bias of association between Penicillins and CRKP infections**


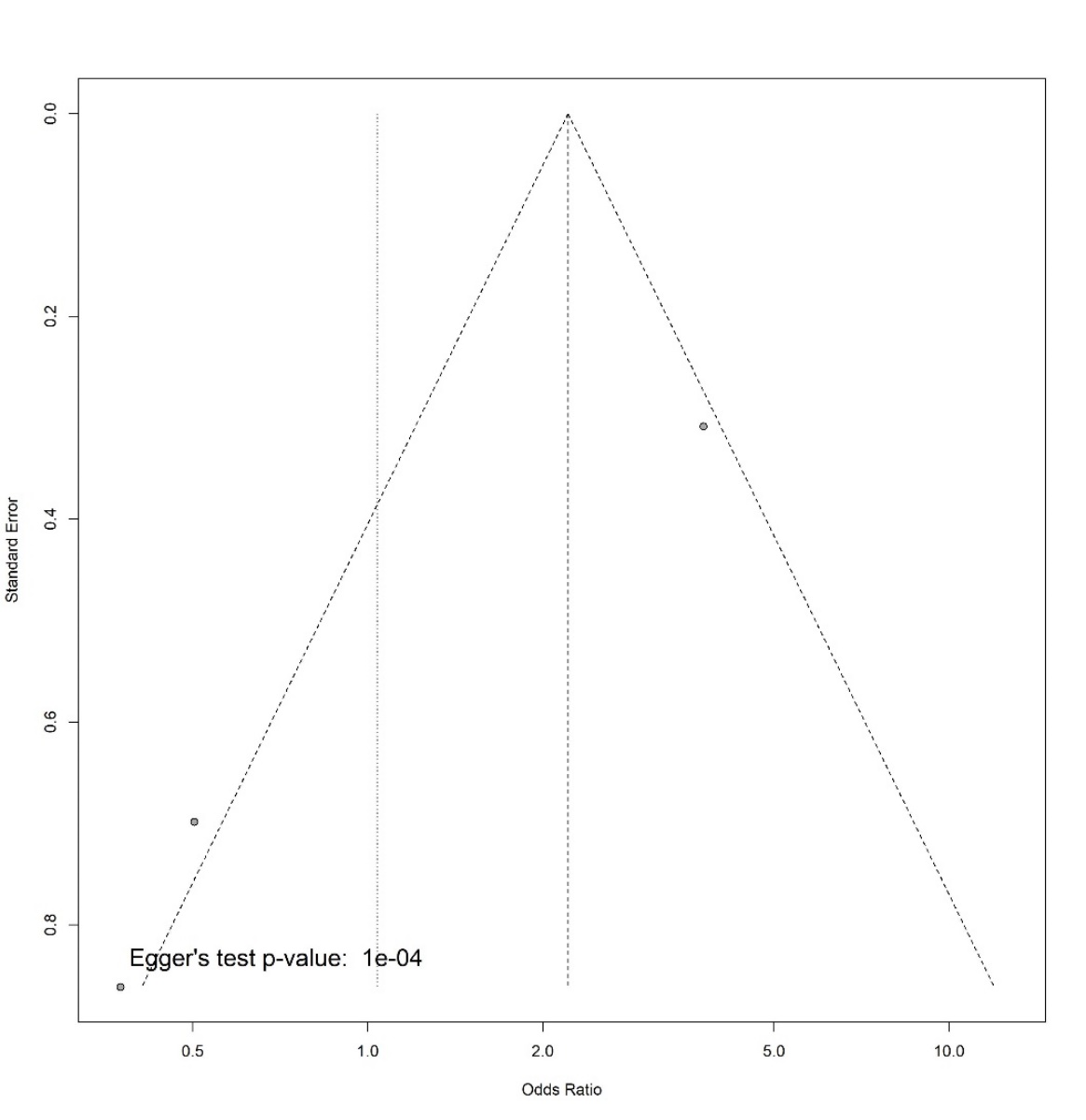


# **Figure S40 Funnel plot for publication bias of association between Tigecycline and CRKP infections**


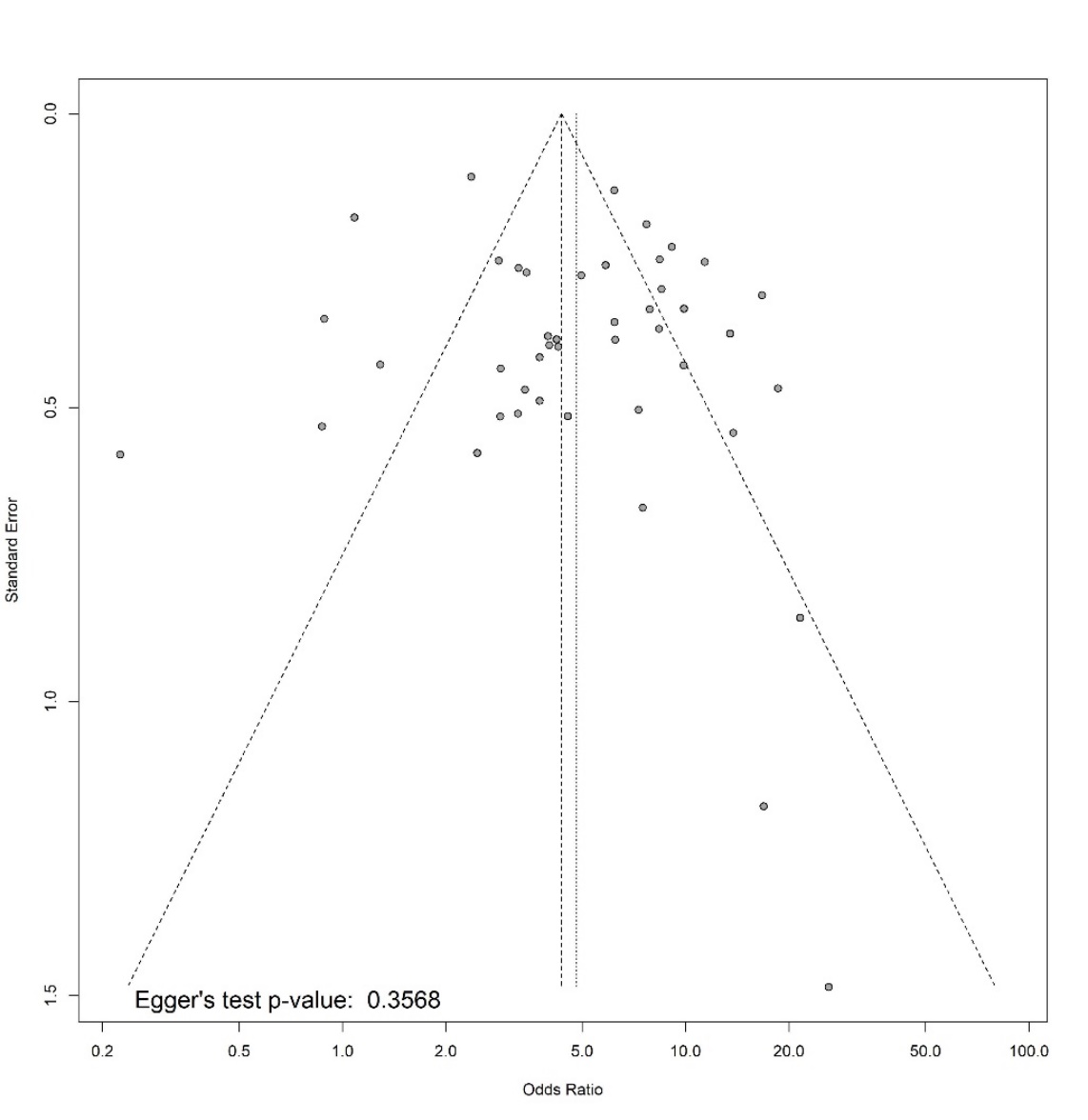


# **Figure S41 Funnel plot for publication bias of association between Carbapenems and CRKP infections**


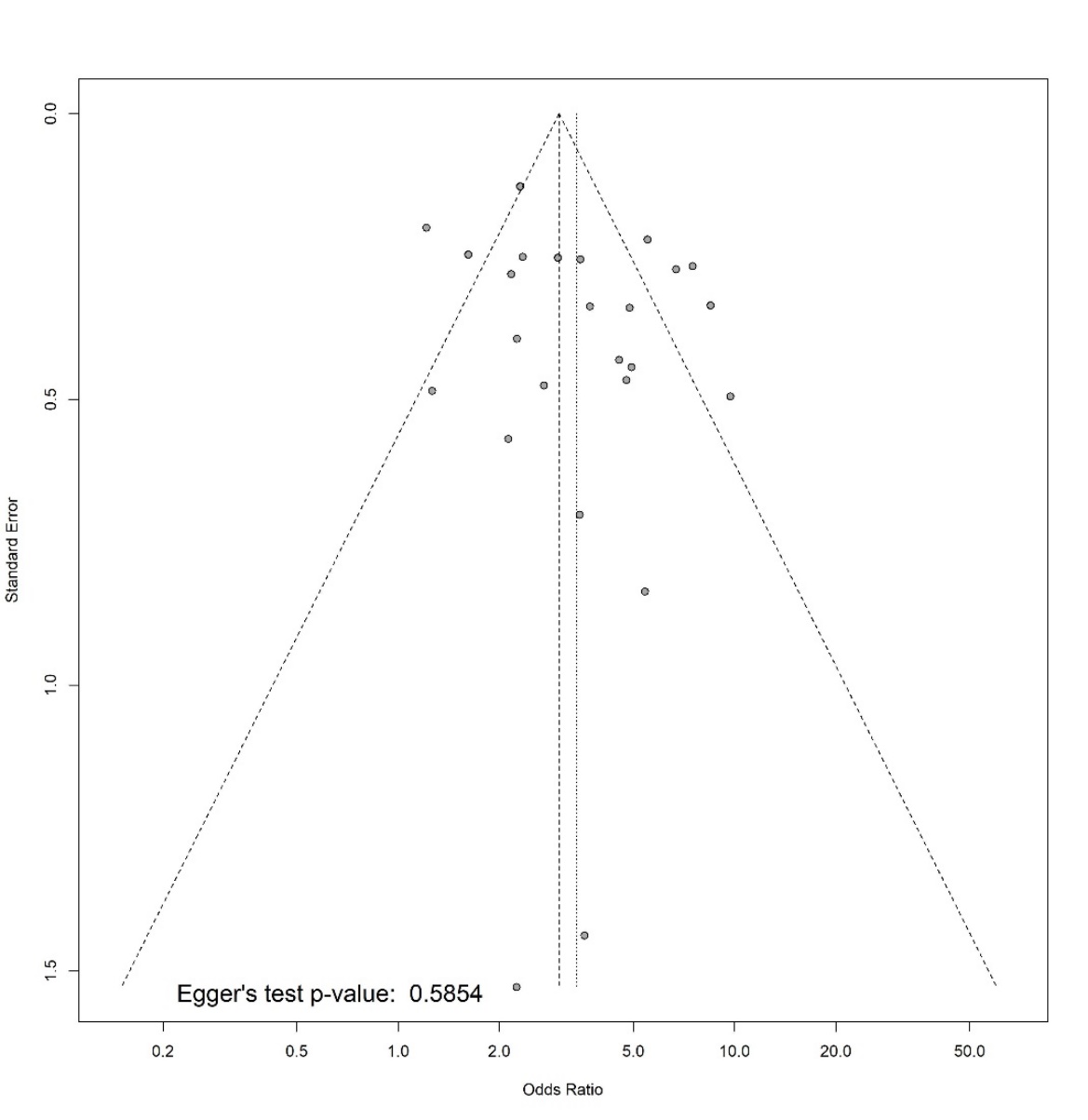


# **Figure S42 Funnel plot for publication bias of association between Tetracyclines and CRKP infections**


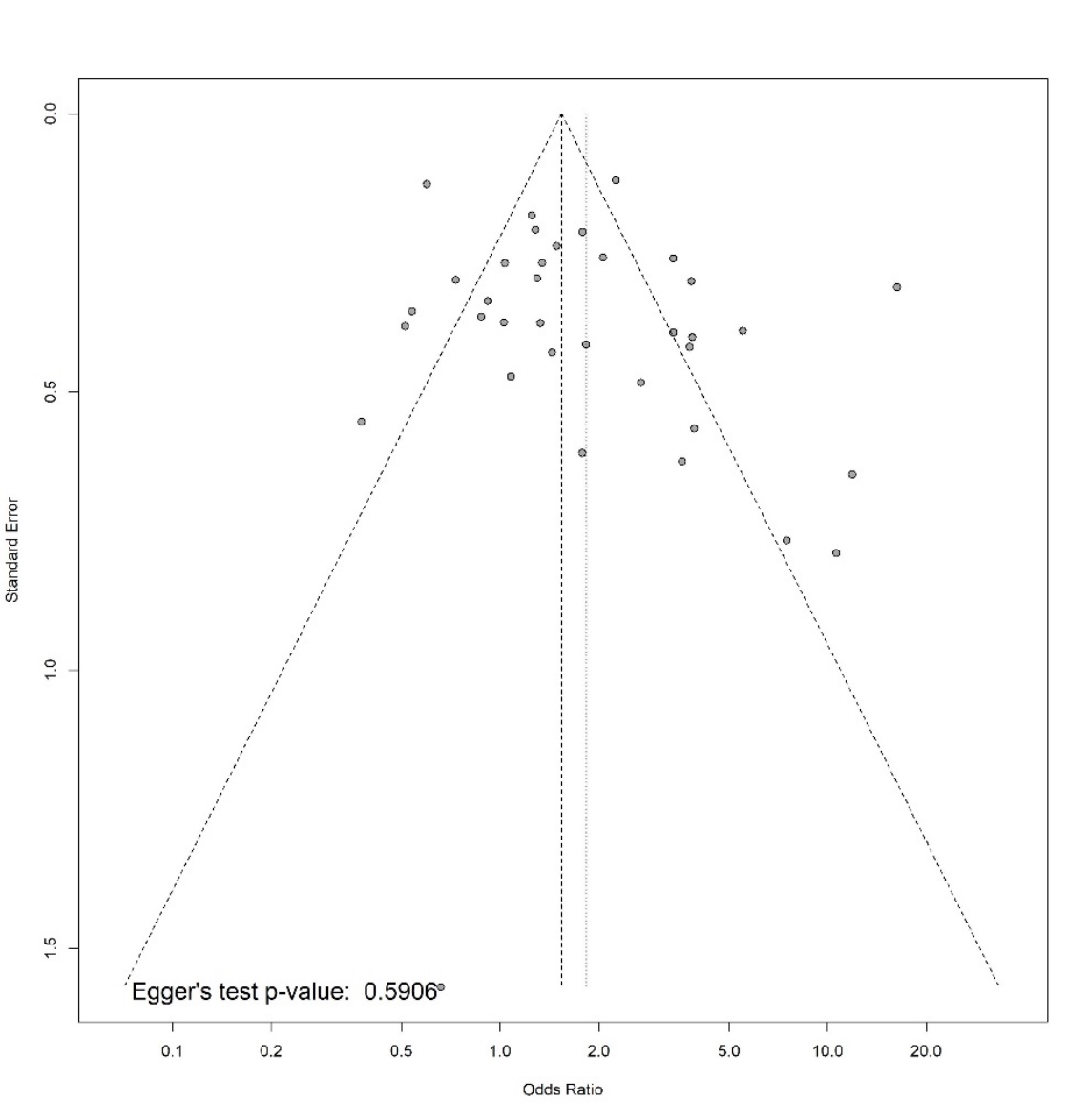


# **Figure S43 Funnel plot for publication bias of association between Cephalosporins and CRKP infections**


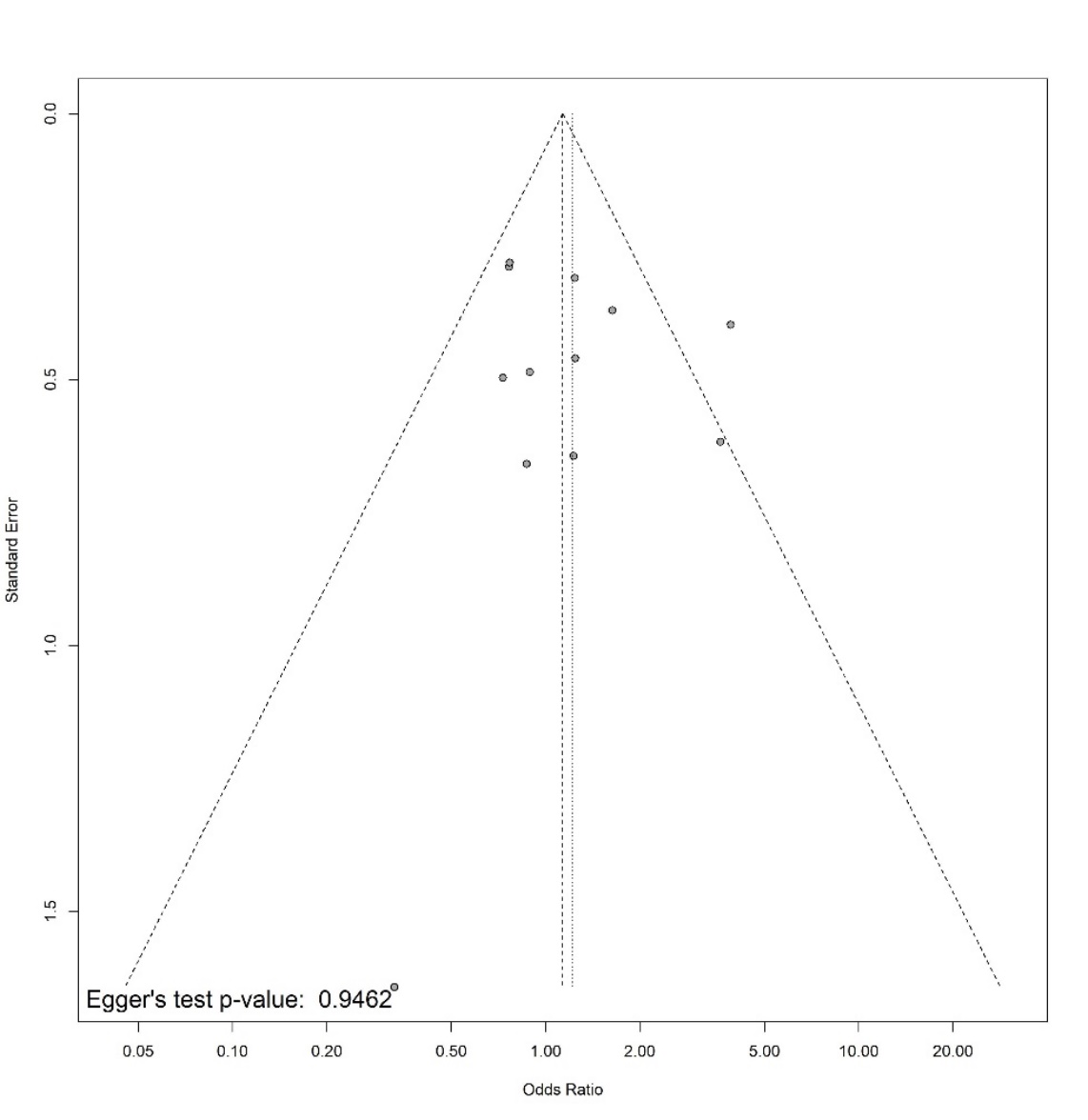


# **Figure S44 Funnel plot for publication bias of association between Nitroimidazoles and CRKP infections**

# **Table S1 Sensitivity analysis for associations between Age and CRKP infections in enrolled studies**

| Removed_Study | Sample_Size_Case | Sample_Size_Control | MD_Random | Lower_CI_Random | Upper_CI_Random | P_Value_Random |
| --- | --- | --- | --- | --- | --- | --- |
| None(n=23) | 2498 | 4775 | 0.45 | -0.69 | 1.6 | 0.44 |
| Falagas, 2007 | 2445 | 4722 | 0.48 | -0.70 | 1.65 | 0.43 |
| Patel, 2008 | 2399 | 4676 | 0.42 | -0.78 | 1.62 | 0.49 |
| Wu, 2011 | 2459 | 4697 | 0.28 | -0.84 | 1.41 | 0.62 |
| Simkins, 2014 | 2485 | 4736 | 0.48 | -0.68 | 1.64 | 0.42 |
| Hu, 2016 | 2433 | 4710 | 0.28 | -0.86 | 1.41 | 0.63 |
| Wang, 2018 | 2450 | 4727 | 0.37 | -0.79 | 1.53 | 0.53 |
| Xiao, 2018 | 2363 | 4482 | 0.47 | -0.75 | 1.69 | 0.45 |
| Zheng, 2018 | 2439 | 4545 | 0.31 | -0.85 | 1.46 | 0.60 |
| Xiao, 2020 | 2394 | 4508 | 0.35 | -0.84 | 1.54 | 0.56 |
| Zhang, 2021 | 2356 | 4633 | 0.54 | -0.66 | 1.74 | 0.38 |
| Chen, 2022 | 2286 | 4281 | 0.29 | -0.89 | 1.47 | 0.63 |
| Lou, 2022 | 2076 | 3827 | 0.43 | -0.82 | 1.68 | 0.50 |
| Arslan, 2023 | 2454 | 4699 | 0.39 | -0.78 | 1.55 | 0.52 |
| lkesen, 2023 | 2366 | 4625 | 0.40 | -0.84 | 1.64 | 0.52 |
| Hussein, 2009 | 2410 | 4402 | 0.60 | -0.57 | 1.76 | 0.32 |
| zheng, 2017 | 2447 | 4724 | 0.41 | -0.75 | 1.58 | 0.49 |
| Li, 2020 | 2334 | 4447 | 0.58 | -0.58 | 1.75 | 0.33 |
| Liu, 2022 | 2424 | 4402 | 0.78 | -0.15 | 1.72 | 0.10 |
| Dai, 2021 | 2407 | 4684 | 0.48 | -0.72 | 1.69 | 0.43 |
| Kritsotakis 2011 | 2402 | 4624 | 0.50 | -0.69 | 1.7 | 0.41 |
| Shilo, 2013 | 2363 | 4648 | 0.65 | -0.49 | 1.8 | 0.26 |
| Liu 2012 | 2473 | 4725 | 0.47 | -0.71 | 1.64 | 0.44 |
| Hussein, 2013 | 2395 | 4561 | 0.56 | -0.62 | 1.75 | 0.35 |

# **Table S2 Sensitivity analysis for associations between sex and CRKP infections in enrolled studies**

| Removed_Study | Sample_Size_Case | Sample_Size_Control | OR_Random | Lower_CI_Random | Upper_CI_Random | P_Value_Random |
| --- | --- | --- | --- | --- | --- | --- |
| None(n=38) | 3056 | 6099 | 1.28 | 1.16 | 1.42 | <0.01 |
| Falagas, 2007 | 3003 | 6046 | 1.29 | 1.16 | 1.43 | <0.01 |
| Patel, 2008 | 2957 | 6000 | 1.30 | 1.17 | 1.44 | <0.01 |
| Wu, 2011 | 3017 | 6021 | 1.29 | 1.17 | 1.43 | <0.01 |
| Correa, 2013 | 3036 | 6059 | 1.28 | 1.15 | 1.42 | <0.01 |
| Simkins, 2014 | 3043 | 6060 | 1.28 | 1.15 | 1.42 | <0.01 |
| Candevir, 2015 | 3009 | 6048 | 1.28 | 1.16 | 1.42 | <0.01 |
| Pereira, 2015 | 3036 | 6063 | 1.27 | 1.15 | 1.4 | <0.01 |
| Pouch, 2015 | 3036 | 6019 | 1.28 | 1.15 | 1.41 | <0.01 |
| Xiao, 2018 | 2921 | 5806 | 1.28 | 1.15 | 1.42 | <0.01 |
| Cienfuegos, 2019 | 3007 | 5810 | 1.27 | 1.15 | 1.41 | <0.01 |
| Liu, 2019 | 3036 | 6030 | 1.28 | 1.16 | 1.42 | <0.01 |
| Chang, 2019 | 3010 | 5860 | 1.28 | 1.16 | 1.43 | <0.01 |
| Wu, 2020 | 3018 | 6069 | 1.28 | 1.15 | 1.42 | <0.01 |
| Xiao, 2020 | 2952 | 5832 | 1.27 | 1.14 | 1.41 | <0.01 |
| Hsu, 2021 | 3020 | 6027 | 1.29 | 1.16 | 1.43 | <0.01 |
| Chen, 2022 | 3027 | 5876 | 1.27 | 1.15 | 1.41 | <0.01 |
| Lou, 2022 | 2634 | 5151 | 1.32 | 1.18 | 1.46 | <0.01 |
| Panda, 2022 | 2948 | 5983 | 1.29 | 1.16 | 1.43 | <0.01 |
| Arslan, 2023 | 3012 | 6023 | 1.28 | 1.16 | 1.43 | <0.01 |
| lkesen, 2023 | 2924 | 5949 | 1.29 | 1.15 | 1.43 | <0.01 |
| Li, 2023 | 2788 | 5680 | 1.27 | 1.14 | 1.42 | <0.01 |
| Wang, 2023 | 2882 | 5880 | 1.30 | 1.17 | 1.45 | <0.01 |
| Cheng, 2024 | 3006 | 6015 | 1.28 | 1.15 | 1.42 | <0.01 |
| Radu, 2024 | 2994 | 5963 | 1.29 | 1.16 | 1.43 | <0.01 |
| Hussein, 2009 | 2968 | 5726 | 1.28 | 1.15 | 1.42 | <0.01 |
| zheng, 2017 | 3005 | 6048 | 1.28 | 1.15 | 1.42 | <0.01 |
| Liu, 2022 | 2982 | 5726 | 1.29 | 1.16 | 1.43 | <0.01 |
| Li, 2022 | 2990 | 6033 | 1.29 | 1.16 | 1.43 | <0.01 |
| Dai, 2021 | 2965 | 6008 | 1.29 | 1.16 | 1.44 | <0.01 |
| Wang, 2022 | 2961 | 5861 | 1.3 | 1.18 | 1.45 | <0.01 |
| Kritsotakis, 2011 | 2960 | 5948 | 1.24 | 1.13 | 1.37 | <0.01 |
| Wu, 2022 | 2988 | 5953 | 1.28 | 1.15 | 1.42 | <0.01 |
| liang, 2022 | 3000 | 6052 | 1.29 | 1.16 | 1.43 | <0.01 |
| Shilo, 2013 | 2921 | 5972 | 1.29 | 1.16 | 1.44 | <0.01 |
| Hoxha, 2016 | 3007 | 6050 | 1.29 | 1.16 | 1.43 | <0.01 |
| Liu 2012 | 3031 | 6049 | 1.29 | 1.16 | 1.43 | <0.01 |
| Hussein, 2013 | 2953 | 5885 | 1.28 | 1.15 | 1.42 | <0.01 |
| Zheng, 2017 | 3025 | 6082 | 1.27 | 1.15 | 1.41 | <0.01 |

# **Table S3 Sensitivity analysis for associations between ICU admission and CRKP infections in enrolled studies**

| Removed_Study | Sample_Size_Case | Sample_Size_Control | OR_Random | Lower_CI_Random | Upper_CI_Random | P_Value_Random |
| --- | --- | --- | --- | --- | --- | --- |
| None(n=34) | 3753 | 7389 | 4.27 | 3.22 | 5.66 | <0.01 |
| Falagas, 2007 | 3700 | 7336 | 4.33 | 3.24 | 5.78 | <0.01 |
| Patel, 2008 | 3654 | 7290 | 4.27 | 3.19 | 5.71 | <0.01 |
| Wu, 2011 | 3714 | 7311 | 4.23 | 3.17 | 5.64 | <0.01 |
| Correa, 2013 | 3733 | 7349 | 4.24 | 3.19 | 5.63 | <0.01 |
| Candevir, 2015 | 3706 | 7338 | 4.27 | 3.2 | 5.69 | <0.01 |
| Wang, 2018 | 3705 | 7341 | 4.15 | 3.13 | 5.50 | <0.01 |
| Xiao, 2018 | 3618 | 7096 | 4.17 | 3.13 | 5.56 | <0.01 |
| Zheng, 2018 | 3694 | 7159 | 4.30 | 3.21 | 5.75 | <0.01 |
| Cienfuegos, 2019 | 3704 | 7100 | 4.39 | 3.3 | 5.84 | <0.01 |
| Chang, 2019 | 3707 | 7150 | 4.18 | 3.14 | 5.58 | <0.01 |
| Xiao, 2020 | 3649 | 7122 | 4.15 | 3.12 | 5.52 | <0.01 |
| Zhang, 2021 | 3611 | 7247 | 4.40 | 3.3 | 5.85 | <0.01 |
| Zuo, 2020 | 3679 | 7315 | 4.45 | 3.37 | 5.88 | <0.01 |
| Cao, 2022 | 3669 | 7302 | 4.22 | 3.16 | 5.64 | <0.01 |
| Chen, 2022 | 3541 | 6895 | 4.37 | 3.27 | 5.83 | <0.01 |
| Chen, 2022 | 3724 | 7166 | 4.14 | 3.12 | 5.49 | <0.01 |
| Lou, 2022 | 3331 | 6441 | 4.33 | 3.24 | 5.80 | <0.01 |
| lkesen, 2023 | 3621 | 7239 | 4.36 | 3.26 | 5.82 | <0.01 |
| Huang, 2023 | 3259 | 6032 | 4.27 | 3.19 | 5.72 | <0.01 |
| Li, 2023 | 3485 | 6970 | 4.22 | 3.15 | 5.65 | <0.01 |
| Wang, 2023 | 3579 | 7170 | 4.28 | 3.19 | 5.73 | <0.01 |
| Cheng, 2024 | 3703 | 7305 | 4.22 | 3.16 | 5.64 | <0.01 |
| Hussein, 2009 | 3665 | 7016 | 4.24 | 3.17 | 5.68 | <0.01 |
| zheng, 2017 | 3702 | 7338 | 4.29 | 3.21 | 5.74 | <0.01 |
| Gupta, 2020 | 3668 | 7363 | 4.35 | 3.26 | 5.8 | <0.01 |
| Li, 2020 | 3589 | 7061 | 4.12 | 3.1 | 5.47 | <0.01 |
| Li, 2022 | 3687 | 7323 | 4.34 | 3.26 | 5.79 | <0.01 |
| Dai, 2021 | 3662 | 7298 | 4.44 | 3.35 | 5.87 | <0.01 |
| Kritsotakis 2011 | 3657 | 7238 | 4.05 | 3.09 | 5.31 | <0.01 |
| Wu, 2022 | 3685 | 7243 | 4.01 | 3.08 | 5.21 | <0.01 |
| liang, 2022 | 3697 | 7342 | 4.37 | 3.28 | 5.82 | <0.01 |
| Shilo, 2013 | 3618 | 7262 | 4.34 | 3.25 | 5.79 | <0.01 |
| Hoxha, 2016 | 3705 | 7340 | 4.45 | 3.37 | 5.87 | <0.01 |
| Liu 2012 | 3728 | 7339 | 4.25 | 3.18 | 5.68 | <0.01 |

# **Table S4 Sensitivity analysis for associations between** **Hospital Stay Before Infection (days) and CRKP infections in enrolled studies**

| Removed_Study | Sample_Size_Case | Sample_Size_Control | MD_Random | Lower_CI_Random | Upper_CI_Random | P_Value_Random |
| --- | --- | --- | --- | --- | --- | --- |
| None(n=6) | 366 | 457 | 14.98 | 3.2 | 26.76 | 0.01 |
| Falagas, 2007 | 313 | 404 | 17.82 | 2.59 | 33.04 | 0.02 |
| Patel, 2008 | 267 | 358 | 14.87 | -1.23 | 30.98 | 0.07 |
| Wu, 2011 | 327 | 379 | 12.97 | 1.66 | 24.28 | 0.02 |
| Kritsotakis, 2011 | 270 | 306 | 16.01 | -0.49 | 32.51 | 0.06 |
| Liu, 2012 | 341 | 407 | 17.68 | 8.65 | 26.71 | <0.01 |
| Gupta, 2020 | 312 | 431 | 11.41 | 1.37 | 21.45 | 0.03 |

# **Table 5 Sensitivity analysis for associations between** **Transfer from other hospital and CRKP infections in enrolled studies**

| Removed_Study | Sample_Size_Case | Sample_Size_Control | OR_Random | Lower_CI_Random | Upper_CI_Random | P_Value_Random |
| --- | --- | --- | --- | --- | --- | --- |
| None(n=4) | 263 | 623 | 2.29 | 1.17 | 4.47 | 0.02 |
| Kritsotakis 2011 | 167 | 472 | 1.97 | 0.87 | 4.45 | 0.10 |
| Cienfuegos, 2019 | 214 | 334 | 3.26 | 2.03 | 5.23 | <0.01 |
| liang, 2022 | 207 | 576 | 2.17 | 0.88 | 5.34 | 0.09 |
| Radu, 2024 | 201 | 487 | 2.06 | 0.87 | 4.86 | 0.10 |

# **Table S6 Sensitivity analysis for associations between Prior hospitalization (within 12 months)and CRKP infections in enrolled studies**

| Removed_Study | Sample_Size_Case | Sample_Size_Control | OR_Random | Lower_CI_Random | Upper_CI_Random | P_Value_Random |
| --- | --- | --- | --- | --- | --- | --- |
| None(n=19) | 1884 | 3577 | 2.08 | 1.67 | 2.6 | <0.01 |
| Falagas, 2007 | 1831 | 3524 | 2.1 | 1.66 | 2.66 | <0.01 |
| Simkins, 2014 | 1871 | 3538 | 2.09 | 1.66 | 2.62 | <0.01 |
| Hu, 2016 | 1819 | 3512 | 2.1 | 1.65 | 2.66 | <0.01 |
| Wang, 2018 | 1836 | 3529 | 2.09 | 1.65 | 2.65 | <0.01 |
| Xiao, 2018 | 1749 | 3284 | 2.03 | 1.6 | 2.58 | <0.01 |
| Cienfuegos, 2019 | 1835 | 3288 | 2.13 | 1.69 | 2.7 | <0.01 |
| Chang, 2019 | 1838 | 3338 | 2.2 | 1.82 | 2.66 | <0.01 |
| Xiao, 2020 | 1780 | 3310 | 2.05 | 1.61 | 2.61 | <0.01 |
| Zhang, 2021 | 1787 | 3536 | 2.05 | 1.63 | 2.58 | <0.01 |
| Chen, 2022 | 1672 | 3083 | 2.12 | 1.66 | 2.72 | <0.01 |
| Lou, 2022 | 1462 | 2629 | 2.02 | 1.59 | 2.56 | <0.01 |
| Çölkesen, 2023 | 1752 | 3427 | 2.08 | 1.63 | 2.65 | <0.01 |
| Radu, 2024 | 1822 | 3441 | 2.01 | 1.62 | 2.49 | <0.01 |
| zheng, 2017 | 1833 | 3526 | 2.06 | 1.63 | 2.6 | <0.01 |
| Gupta, 2020 | 1830 | 3551 | 1.99 | 1.62 | 2.45 | <0.01 |
| Li, 2020 | 1720 | 3249 | 2.15 | 1.69 | 2.72 | <0.01 |
| liang, 2022 | 1828 | 3530 | 2.11 | 1.68 | 2.65 | <0.01 |
| Hoxha, 2016 | 1836 | 3532 | 2.16 | 1.74 | 2.68 | <0.01 |
| Vardakas, 2015 | 1811 | 3559 | 2.06 | 1.64 | 2.59 | <0.01 |

# **Table S7 Sensitivity analysis for associations between prior hospitalization (within 12 months) and CRKP infections in enrolled studies**

| Removed_Study | Sample_Size_Case | Sample_Size_Control | OR_Random | Lower_CI_Random | Upper_CI_Random | P_Value_Random |
| --- | --- | --- | --- | --- | --- | --- |
| None(n=19) | 1884 | 3577 | 2.08 | 1.67 | 2.6 | <0.01 |
| Falagas, 2007 | 1831 | 3524 | 2.1 | 1.66 | 2.66 | <0.01 |
| Simkins, 2014 | 1871 | 3538 | 2.09 | 1.66 | 2.62 | <0.01 |
| Hu, 2016 | 1819 | 3512 | 2.1 | 1.65 | 2.66 | <0.01 |
| Wang, 2018 | 1836 | 3529 | 2.09 | 1.65 | 2.65 | <0.01 |
| Xiao, 2018 | 1749 | 3284 | 2.03 | 1.6 | 2.58 | <0.01 |
| Cienfuegos, 2019 | 1835 | 3288 | 2.13 | 1.69 | 2.7 | <0.01 |
| Chang, 2019 | 1838 | 3338 | 2.2 | 1.82 | 2.66 | <0.01 |
| Xiao, 2020 | 1780 | 3310 | 2.05 | 1.61 | 2.61 | <0.01 |
| Zhang, 2021 | 1787 | 3536 | 2.05 | 1.63 | 2.58 | <0.01 |
| Chen, 2022 | 1672 | 3083 | 2.12 | 1.66 | 2.72 | <0.01 |
| Lou, 2022 | 1462 | 2629 | 2.02 | 1.59 | 2.56 | <0.01 |
| Çölkesen, 2023 | 1752 | 3427 | 2.08 | 1.63 | 2.65 | <0.01 |
| Radu, 2024 | 1822 | 3441 | 2.01 | 1.62 | 2.49 | <0.01 |
| zheng, 2017 | 1833 | 3526 | 2.06 | 1.63 | 2.6 | <0.01 |
| Gupta, 2020 | 1830 | 3551 | 1.99 | 1.62 | 2.45 | <0.01 |
| Li, 2020 | 1720 | 3249 | 2.15 | 1.69 | 2.72 | <0.01 |
| liang, 2022 | 1828 | 3530 | 2.11 | 1.68 | 2.65 | <0.01 |
| Hoxha, 2016 | 1836 | 3532 | 2.16 | 1.74 | 2.68 | <0.01 |
| Vardakas, 2015 | 1811 | 3559 | 2.06 | 1.64 | 2.59 | <0.01 |

# **Table S8 Sensitivity analysis for associations between Nasogastric catheter and CRKP infections in enrolled studies**

| Removed_Study | Sample_Size_Case | Sample_Size_Control | OR_Random | Lower_CI_Random | Upper_CI_Random | P_Value_Random |
| --- | --- | --- | --- | --- | --- | --- |
| None(n=11) | 1819 | 3830 | 2.41 | 1.54 | 3.77 | <0.01 |
| Falagas, 2007 | 1766 | 3779 | 2.64 | 1.68 | 4.14 | <0.01 |
| Candevir, 2015 | 1772 | 3779 | 2.33 | 1.44 | 3.77 | <0.01 |
| Zheng, 2018 | 1760 | 3600 | 2.35 | 1.44 | 3.84 | <0.01 |
| Zhang, 2021 | 1677 | 3688 | 2.32 | 1.42 | 3.79 | <0.01 |
| Cao, 2022 | 1735 | 3743 | 2.24 | 1.4 | 3.58 | <0.01 |
| Chen, 2022 | 1607 | 3336 | 2.75 | 1.81 | 4.17 | <0.01 |
| Lou, 2022 | 1397 | 2882 | 2.43 | 1.48 | 4 | <0.01 |
| Huang, 2023 | 1325 | 2473 | 2.31 | 1.41 | 3.79 | <0.01 |
| zheng, 2017 | 1768 | 3779 | 2.53 | 1.57 | 4.08 | <0.01 |
| Li, 2020 | 1655 | 3502 | 2.1 | 1.4 | 3.15 | <0.01 |
| Dai, 2021 | 1728 | 3739 | 2.55 | 1.58 | 4.12 | <0.01 |

# **Table S9 Sensitivity analysis for associations between Parenteral nutrition and CRKP infections in enrolled studies**

| Removed_Study | Sample_Size_Case | Sample_Size_Control | OR_Random | Lower_CI_Random | Upper_CI_Random | P_Value_Random |
| --- | --- | --- | --- | --- | --- | --- |
| None(n=10) | 1456 | 2987 | 1.78 | 1.18 | 2.68 | 0.01 |
| Wang, 2018 | 1408 | 2939 | 1.92 | 1.27 | 2.89 | <0.01 |
| Zhang, 2021 | 1314 | 2845 | 1.72 | 1.08 | 2.75 | 0.02 |
| Chen, 2022 | 1244 | 2493 | 1.89 | 1.2 | 2.98 | 0.01 |
| Çölkesen, 2023 | 1324 | 2837 | 1.73 | 1.08 | 2.76 | 0.02 |
| Huang, 2023 | 962 | 1630 | 1.89 | 1.2 | 2.99 | 0.01 |
| Hussein, 2009 | 1368 | 2614 | 1.91 | 1.24 | 2.93 | <0.01 |
| zheng, 2017 | 1405 | 2936 | 1.66 | 1.08 | 2.53 | 0.02 |
| Gupta, 2020 | 1402 | 2961 | 1.72 | 1.12 | 2.64 | 0.01 |
| Li, 2020 | 1292 | 2659 | 1.52 | 1.12 | 2.06 | 0.01 |
| Vardakas, 2015 | 1385 | 2969 | 1.84 | 1.18 | 2.86 | 0.01 |

# **Table S10 Sensitivity analysis for associations between Mechanical ventilation and CRKP infections in enrolled studies**

| Removed_Study | Sample_Size_Case | Sample_Size_Control | OR_Random | Lower_CI_Random | Upper_CI_Random | P_Value_Random |
| --- | --- | --- | --- | --- | --- | --- |
| None(n=35) | 3794 | 7357 | 3.61 | 2.72 | 4.78 | <0.01 |
| Falagas, 2007 | 3741 | 7304 | 3.67 | 2.75 | 4.89 | <0.01 |
| Patel, 2008 | 3695 | 7258 | 3.54 | 2.65 | 4.72 | <0.01 |
| Wu, 2011 | 3755 | 7279 | 3.61 | 2.7 | 4.82 | <0.01 |
| Correa, 2013 | 3774 | 7317 | 3.66 | 2.75 | 4.86 | <0.01 |
| Candevir, 2015 | 3747 | 7306 | 3.69 | 2.78 | 4.91 | <0.01 |
| Wang, 2018 | 3746 | 7309 | 3.6 | 2.7 | 4.8 | <0.01 |
| Xiao, 2018 | 3659 | 7064 | 3.47 | 2.62 | 4.58 | <0.01 |
| Zheng, 2018 | 3735 | 7127 | 3.49 | 2.63 | 4.63 | <0.01 |
| Cienfuegos, 2019 | 3745 | 7068 | 3.63 | 2.71 | 4.84 | <0.01 |
| Wu, 2020 | 3756 | 7327 | 3.78 | 2.88 | 4.95 | <0.01 |
| Xiao, 2020 | 3690 | 7090 | 3.46 | 2.62 | 4.56 | <0.01 |
| Zhang, 2021 | 3652 | 7215 | 3.72 | 2.8 | 4.94 | <0.01 |
| Zhang, 2021 | 3697 | 7316 | 3.61 | 2.7 | 4.82 | <0.01 |
| Zuo, 2020 | 3720 | 7283 | 3.7 | 2.79 | 4.92 | <0.01 |
| Cao, 2022 | 3710 | 7270 | 3.57 | 2.67 | 4.76 | <0.01 |
| Chen, 2022 | 3582 | 6863 | 3.73 | 2.81 | 4.95 | <0.01 |
| Chen, 2022 | 3765 | 7134 | 3.52 | 2.65 | 4.67 | <0.01 |
| Lou, 2022 | 3372 | 6409 | 3.58 | 2.68 | 4.79 | <0.01 |
| Çölkesen, 2023 | 3662 | 7207 | 3.69 | 2.77 | 4.91 | <0.01 |
| Huang, 2023 | 3300 | 6000 | 3.63 | 2.72 | 4.86 | <0.01 |
| Li, 2023 | 3526 | 6938 | 3.55 | 2.66 | 4.74 | <0.01 |
| Cheng, 2024 | 3744 | 7273 | 3.45 | 2.63 | 4.54 | <0.01 |
| Hussein, 2009 | 3706 | 6984 | 3.66 | 2.74 | 4.89 | <0.01 |
| zheng, 2017 | 3743 | 7306 | 3.63 | 2.72 | 4.84 | <0.01 |
| Gupta, 2020 | 3740 | 7331 | 3.52 | 2.65 | 4.66 | <0.01 |
| Li, 2020 | 3630 | 7029 | 3.62 | 2.71 | 4.84 | <0.01 |
| Dai, 2021 | 3703 | 7266 | 3.71 | 2.79 | 4.93 | <0.01 |
| Wang, 2022 | 3699 | 7119 | 3.61 | 2.7 | 4.83 | <0.01 |
| Kritsotakis 2011 | 3698 | 7206 | 3.52 | 2.65 | 4.69 | <0.01 |
| Wu, 2022 | 3726 | 7211 | 3.42 | 2.62 | 4.48 | <0.01 |
| liang, 2022 | 3738 | 7310 | 3.65 | 2.74 | 4.87 | <0.01 |
| Shilo, 2013 | 3659 | 7230 | 3.72 | 2.8 | 4.93 | <0.01 |
| Liu 2012 | 3769 | 7307 | 3.59 | 2.69 | 4.78 | <0.01 |
| Hussein, 2013 | 3691 | 7143 | 3.7 | 2.77 | 4.92 | <0.01 |
| Vardakas, 2015 | 3721 | 7339 | 3.63 | 2.74 | 4.82 | <0.01 |

# **Table S11 Sensitivity analysis for associations between Indwelling urinary catheter and CRKP infections in enrolled studies**

| Removed_Study | Sample_Size_Case | Sample_Size_Control | OR_Random | Lower_CI_Random | Upper_CI_Random | P_Value_Random |
| --- | --- | --- | --- | --- | --- | --- |
| None(n=27) | 3296 | 6195 | 2.96 | 2.14 | 4.09 | <0.01 |
| Falagas, 2007 | 3243 | 6142 | 3.08 | 2.22 | 4.27 | <0.01 |
| Correa, 2013 | 3276 | 6155 | 3.11 | 2.26 | 4.28 | <0.01 |
| Candevir, 2015 | 3249 | 6144 | 3.03 | 2.17 | 4.22 | <0.01 |
| Wang, 2018 | 3248 | 6147 | 2.9 | 2.08 | 4.04 | <0.01 |
| Xiao, 2018 | 3161 | 5902 | 2.79 | 2.03 | 3.83 | <0.01 |
| Zheng, 2018 | 3237 | 5965 | 2.9 | 2.07 | 4.05 | <0.01 |
| Cienfuegos, 2019 | 3247 | 5906 | 2.98 | 2.13 | 4.18 | <0.01 |
| Xiao, 2020 | 3192 | 5928 | 2.76 | 2.04 | 3.74 | <0.01 |
| Zhang, 2021 | 3154 | 6053 | 3 | 2.14 | 4.2 | <0.01 |
| Zhang, 2021 | 3199 | 6154 | 2.92 | 2.09 | 4.09 | <0.01 |
| Zuo, 2020 | 3222 | 6121 | 3.08 | 2.22 | 4.27 | <0.01 |
| Chen, 2022 | 3084 | 5701 | 3.09 | 2.22 | 4.29 | <0.01 |
| Lou, 2022 | 2874 | 5247 | 2.91 | 2.08 | 4.08 | <0.01 |
| Çölkesen, 2023 | 3164 | 6045 | 3 | 2.15 | 4.17 | <0.01 |
| Huang, 2023 | 2802 | 4838 | 3.07 | 2.21 | 4.27 | <0.01 |
| Li, 2023 | 3028 | 5776 | 2.89 | 2.06 | 4.04 | <0.01 |
| Cheng, 2024 | 3246 | 6111 | 2.85 | 2.05 | 3.95 | <0.01 |
| Radu, 2024 | 3234 | 6059 | 3.02 | 2.17 | 4.22 | <0.01 |
| zheng, 2017 | 3245 | 6144 | 3.02 | 2.16 | 4.21 | <0.01 |
| Gupta, 2020 | 3211 | 6169 | 2.94 | 2.1 | 4.1 | <0.01 |
| Li, 2020 | 3132 | 5867 | 2.83 | 2.04 | 3.93 | <0.01 |
| Dai, 2021 | 3205 | 6104 | 3.07 | 2.21 | 4.26 | <0.01 |
| Wang, 2022 | 3201 | 5957 | 2.99 | 2.14 | 4.19 | <0.01 |
| Kritsotakis 2011 | 3145 | 6044 | 2.84 | 2.05 | 3.95 | <0.01 |
| Shilo, 2013 | 3161 | 6068 | 3.02 | 2.16 | 4.22 | <0.01 |
| Liu 2012 | 3271 | 6145 | 2.95 | 2.11 | 4.12 | <0.01 |
| Zheng, 2017 | 3265 | 6178 | 2.93 | 2.1 | 4.09 | <0.01 |

# **Table S12 Sensitivity analysis for associations between Endoscopy and CRKP infections in enrolled studies**

| Removed_Study | Sample_Size_Case | Sample_Size_Control | OR_Random | Lower_CI_Random | Upper_CI_Random | P_Value_Random |
| --- | --- | --- | --- | --- | --- | --- |
| None(n=5) | 1089 | 2393 | 4.08 | 1.4 | 11.92 | 0.01 |
| Cao, 2022 | 1005 | 2306 | 3.59 | 1.07 | 12.06 | 0.04 |
| Chen, 2022 | 877 | 1899 | 5.96 | 2.07 | 17.15 | <0.01 |
| Huang, 2023 | 595 | 1036 | 4.89 | 1.2 | 19.85 | 0.03 |
| Li, 2020 | 925 | 2065 | 2.38 | 1.15 | 4.92 | 0.02 |
| Shilo, 2013 | 954 | 2266 | 4.35 | 1.08 | 17.52 | 0.04 |

# **Table S13 Sensitivity analysis for associations between Tracheal cannula and CRKP infections in enrolled studies**

| Removed_Study | Sample_Size_Case | Sample_Size_Control | OR_Random | Lower_CI_Random | Upper_CI_Random | P_Value_Random |
| --- | --- | --- | --- | --- | --- | --- |
| None(n=10) | 1275 | 2530 | 3.72 | 2.1 | 6.6 | <0.01 |
| Wu, 2011 | 1236 | 2452 | 3.84 | 2.05 | 7.23 | <0.01 |
| Candevir, 2015 | 1228 | 2479 | 4.07 | 2.22 | 7.47 | <0.01 |
| Zheng, 2018 | 1216 | 2300 | 3.45 | 1.86 | 6.42 | <0.01 |
| Zhang, 2021 | 1133 | 2388 | 4.16 | 2.28 | 7.57 | <0.01 |
| Chen, 2022 | 1063 | 2036 | 4.32 | 2.46 | 7.58 | <0.01 |
| Lou, 2022 | 853 | 1582 | 3.75 | 1.97 | 7.15 | <0.01 |
| Li, 2020 | 1111 | 2202 | 3.3 | 1.83 | 5.96 | <0.01 |
| Li, 2022 | 1209 | 2464 | 3.63 | 1.93 | 6.82 | <0.01 |
| Wu, 2022 | 1207 | 2384 | 3.1 | 1.86 | 5.19 | <0.01 |
| liang, 2022 | 1219 | 2483 | 3.77 | 2 | 7.12 | <0.01 |

# **Table S14 Sensitivity analysis for associations between Tracheostomy and CRKP infections in enrolled studies**

| Removed_Study | Sample_Size_Case | Sample_Size_Control | OR_Random | Lower_CI_Random | Upper_CI_Random | P_Value_Random |
| --- | --- | --- | --- | --- | --- | --- |
| None(n=19) | 2267 | 4793 | 2.84 | 1.91 | 4.21 | <0.01 |
| Falagas, 2007 | 2214 | 4740 | 2.82 | 1.85 | 4.28 | <0.01 |
| Wu, 2011 | 2228 | 4715 | 2.95 | 1.95 | 4.47 | <0.01 |
| Zheng, 2018 | 2208 | 4563 | 2.75 | 1.82 | 4.18 | <0.01 |
| Chang, 2019 | 2221 | 4554 | 2.64 | 1.79 | 3.9 | <0.01 |
| Chen, 2022 | 2055 | 4299 | 2.98 | 1.97 | 4.5 | <0.01 |
| Lou, 2022 | 1845 | 3845 | 2.67 | 1.79 | 4 | <0.01 |
| Çölkesen, 2023 | 2135 | 4643 | 3.05 | 2.06 | 4.52 | <0.01 |
| Huang, 2023 | 1773 | 3436 | 2.96 | 1.94 | 4.5 | <0.01 |
| Li, 2020 | 2103 | 4465 | 2.78 | 1.82 | 4.23 | <0.01 |
| Dai, 2021 | 2176 | 4702 | 2.95 | 1.94 | 4.47 | <0.01 |
| Wang, 2022 | 2172 | 4555 | 2.83 | 1.85 | 4.33 | <0.01 |
| Kritsotakis 2011 | 2171 | 4642 | 2.5 | 1.78 | 3.51 | <0.01 |
| Wu, 2022 | 2199 | 4647 | 2.69 | 1.82 | 3.96 | <0.01 |
| liang, 2022 | 2211 | 4746 | 2.82 | 1.85 | 4.29 | <0.01 |
| Shilo, 2013 | 2132 | 4666 | 2.99 | 1.99 | 4.49 | <0.01 |
| Hoxha, 2016 | 2218 | 4744 | 2.91 | 1.92 | 4.42 | <0.01 |
| Liu 2012 | 2242 | 4743 | 2.93 | 1.95 | 4.4 | <0.01 |
| Zheng, 2017 | 2236 | 4776 | 2.92 | 1.94 | 4.4 | <0.01 |

# **Table S15 Sensitivity analysis for associations between Dialysis and CRKP infections in enrolled studies**

| Removed_Study | Sample_Size_Case | Sample_Size_Control | OR_Random | Lower_CI_Random | Upper_CI_Random | P_Value_Random |
| --- | --- | --- | --- | --- | --- | --- |
| None(n=15) | 953 | 1684 | 2.68 | 1.82 | 3.96 | <0.01 |
| Correa, 2013 | 933 | 1644 | 2.68 | 1.78 | 4.05 | <0.01 |
| Simkins, 2014 | 940 | 1645 | 2.81 | 1.89 | 4.18 | <0.01 |
| Pouch, 2015 | 933 | 1604 | 3 | 2.09 | 4.31 | <0.01 |
| Wang, 2018 | 905 | 1636 | 2.61 | 1.73 | 3.95 | <0.01 |
| Xiao, 2018 | 818 | 1391 | 2.48 | 1.65 | 3.73 | <0.01 |
| Cienfuegos, 2019 | 904 | 1395 | 2.75 | 1.8 | 4.19 | <0.01 |
| Wu, 2020 | 915 | 1654 | 2.69 | 1.78 | 4.06 | <0.01 |
| Xiao, 2020 | 849 | 1417 | 2.43 | 1.69 | 3.49 | <0.01 |
| Çölkesen, 2023 | 821 | 1534 | 2.84 | 1.9 | 4.26 | <0.01 |
| Gupta, 2020 | 899 | 1658 | 2.56 | 1.7 | 3.86 | <0.01 |
| Dai, 2021 | 862 | 1593 | 2.65 | 1.74 | 4.02 | <0.01 |
| Hoxha, 2016 | 904 | 1635 | 2.74 | 1.82 | 4.14 | <0.01 |
| Liu 2012 | 928 | 1634 | 2.81 | 1.91 | 4.13 | <0.01 |
| Hussein, 2013 | 850 | 1470 | 2.55 | 1.67 | 3.9 | <0.01 |
| Vardakas, 2015 | 881 | 1666 | 2.63 | 1.77 | 3.91 | <0.01 |

# **Table S16 Sensitivity analysis for associations between Surgical drainage and CRKP infections in enrolled studies**

| Removed_Study | Sample_Size_Case | Sample_Size_Control | OR_Random | Lower_CI_Random | Upper_CI_Random | P_Value_Random |
| --- | --- | --- | --- | --- | --- | --- |
| None(n=10) | 1612 | 3225 | 1.52 | 1.15 | 2.01 | <0.01 |
| Zheng, 2017 | 1581 | 3208 | 1.5 | 1.12 | 2 | 0.01 |
| zheng, 2017 | 1561 | 3174 | 1.48 | 1.1 | 2.01 | 0.01 |
| Zheng, 2018 | 1553 | 2995 | 1.46 | 1.08 | 1.98 | 0.01 |
| Zhang, 2021 | 1470 | 3083 | 1.5 | 1.09 | 2.07 | 0.01 |
| Zhang, 2021 | 1515 | 3184 | 1.52 | 1.14 | 2.02 | <0.01 |
| Zuo, 2020 | 1538 | 3151 | 1.63 | 1.25 | 2.12 | <0.01 |
| Lou, 2022 | 1190 | 2277 | 1.5 | 1.07 | 2.09 | 0.02 |
| Wu, 2022 | 1544 | 3079 | 1.49 | 1.09 | 2.03 | 0.01 |
| Huang, 2023 | 1118 | 1868 | 1.45 | 1.05 | 2 | 0.02 |
| Wang, 2023 | 1438 | 3006 | 1.77 | 1.49 | 2.09 | <0.01 |

# **Table S17 Sensitivity analysis for associations between Peripheral Catheter and CRKP infections in enrolled studies**

| Removed_Study | Sample_Size_Case | Sample_Size_Control | OR_Random | Lower_CI_Random | Upper_CI_Random | P_Value_Random |
| --- | --- | --- | --- | --- | --- | --- |
| None(n=6) | 1098 | 2358 | 2.4 | 1.49 | 3.88 | <0.01 |
| Candevir, 2015 | 1051 | 2307 | 2.4 | 1.38 | 4.19 | <0.01 |
| Li, 2020 | 934 | 2030 | 2.03 | 1.28 | 3.22 | <0.01 |
| Zhang, 2021 | 1001 | 2317 | 2.41 | 1.38 | 4.2 | <0.01 |
| Cao, 2022 | 1014 | 2271 | 2.16 | 1.29 | 3.61 | <0.01 |
| Chen, 2022 | 886 | 1864 | 2.65 | 1.51 | 4.65 | <0.01 |
| Huang, 2023 | 604 | 1001 | 2.89 | 1.83 | 4.56 | <0.01 |

# **Table S18 Sensitivity analysis for associations between Central venous catheter and CRKP infections in enrolled studies**

| Removed_Study | Sample_Size_Case | Sample_Size_Control | OR_Random | Lower_CI_Random | Upper_CI_Random | P_Value_Random |
| --- | --- | --- | --- | --- | --- | --- |
| None(n=29) | 3388 | 6623 | 3.39 | 2.4 | 4.79 | <0.01 |
| Falagas, 2007 | 3363 | 6598 | 3.43 | 2.42 | 4.87 | <0.01 |
| Patel, 2008 | 3289 | 6524 | 3.28 | 2.31 | 4.67 | <0.01 |
| Correa, 2013 | 3368 | 6583 | 3.37 | 2.36 | 4.8 | <0.01 |
| Candevir, 2015 | 3341 | 6572 | 3.59 | 2.56 | 5.03 | <0.01 |
| Xiao, 2018 | 3253 | 6330 | 3.23 | 2.28 | 4.57 | <0.01 |
| Zheng, 2018 | 3329 | 6393 | 3.33 | 2.33 | 4.76 | <0.01 |
| Cienfuegos, 2019 | 3339 | 6334 | 3.39 | 2.36 | 4.85 | <0.01 |
| Liu, 2019 | 3368 | 6554 | 3.51 | 2.47 | 4.98 | <0.01 |
| Xiao, 2020 | 3284 | 6356 | 3.2 | 2.28 | 4.5 | <0.01 |
| Zhang, 2021 | 3246 | 6481 | 3.5 | 2.45 | 4.99 | <0.01 |
| Zhang, 2021 | 3291 | 6582 | 3.38 | 2.36 | 4.83 | <0.01 |
| Zuo, 2020 | 3314 | 6549 | 3.31 | 2.32 | 4.72 | <0.01 |
| Cao, 2022 | 3304 | 6536 | 3.29 | 2.31 | 4.69 | <0.01 |
| Chen, 2022 | 3176 | 6129 | 3.53 | 2.49 | 5.01 | <0.01 |
| Lou, 2022 | 2966 | 5675 | 3.45 | 2.41 | 4.93 | <0.01 |
| Çölkesen, 2023 | 3256 | 6473 | 3.44 | 2.4 | 4.92 | <0.01 |
| Huang, 2023 | 2894 | 5266 | 3.41 | 2.37 | 4.89 | <0.01 |
| Li, 2023 | 3120 | 6204 | 3.41 | 2.38 | 4.9 | <0.01 |
| Cheng, 2024 | 3338 | 6539 | 3.29 | 2.31 | 4.69 | <0.01 |
| Hussein, 2009 | 3300 | 6250 | 3.45 | 2.41 | 4.94 | <0.01 |
| Li, 2020 | 3224 | 6295 | 3.24 | 2.29 | 4.58 | <0.01 |
| Li, 2022 | 3322 | 6557 | 3.62 | 2.6 | 5.03 | <0.01 |
| Dai, 2021 | 3297 | 6532 | 3.46 | 2.42 | 4.95 | <0.01 |
| Kritsotakis 2011 | 3292 | 6472 | 3.14 | 2.27 | 4.34 | <0.01 |
| liang, 2022 | 3332 | 6576 | 3.46 | 2.42 | 4.94 | <0.01 |
| Shilo, 2013 | 3253 | 6496 | 3.48 | 2.44 | 4.96 | <0.01 |
| Liu 2012 | 3363 | 6573 | 3.37 | 2.36 | 4.81 | <0.01 |
| Hussein, 2013 | 3285 | 6409 | 3.42 | 2.39 | 4.9 | <0.01 |
| Zheng, 2017 | 3357 | 6606 | 3.29 | 2.33 | 4.66 | <0.01 |

# **Table S19 Sensitivity analysis for associations between cardiovascular disease and CRKP infections in enrolled studies**

| Removed_Study | Sample_Size_Case | Sample_Size_Control | OR_Random | Lower_CI_Random | Upper_CI_Random | P_Value_Random |
| --- | --- | --- | --- | --- | --- | --- |
| None(n=25) | 2640 | 4674 | 1.34 | 1.08 | 1.66 | 0.01 |
| Falagas, 2007 | 2587 | 4621 | 1.38 | 1.1 | 1.71 | <0.01 |
| Patel, 2008 | 2541 | 4575 | 1.33 | 1.06 | 1.67 | 0.01 |
| Hu, 2016 | 2575 | 4609 | 1.32 | 1.06 | 1.66 | 0.01 |
| Wang, 2018 | 2592 | 4626 | 1.3 | 1.05 | 1.62 | 0.02 |
| Zheng, 2018 | 2581 | 4444 | 1.31 | 1.05 | 1.64 | 0.02 |
| Cienfuegos, 2019 | 2591 | 4385 | 1.36 | 1.09 | 1.71 | 0.01 |
| Zhang, 2021 | 2498 | 4532 | 1.39 | 1.12 | 1.72 | <0.01 |
| Zhang, 2021 | 2543 | 4633 | 1.31 | 1.05 | 1.63 | 0.02 |
| Zuo, 2020 | 2566 | 4600 | 1.31 | 1.05 | 1.62 | 0.01 |
| Cao, 2022 | 2556 | 4587 | 1.36 | 1.09 | 1.7 | 0.01 |
| Chen, 2022 | 2428 | 4180 | 1.39 | 1.12 | 1.72 | <0.01 |
| Çölkesen, 2023 | 2508 | 4524 | 1.29 | 1.04 | 1.59 | 0.02 |
| Huang, 2023 | 2146 | 3317 | 1.33 | 1.05 | 1.67 | 0.02 |
| Li, 2023 | 2372 | 4255 | 1.36 | 1.08 | 1.7 | 0.01 |
| Cheng, 2024 | 2590 | 4590 | 1.27 | 1.04 | 1.55 | 0.02 |
| Radu, 2024 | 2578 | 4538 | 1.38 | 1.11 | 1.71 | <0.01 |
| zheng, 2017 | 2589 | 4623 | 1.35 | 1.07 | 1.69 | 0.01 |
| Li, 2020 | 2476 | 4346 | 1.36 | 1.08 | 1.71 | 0.01 |
| Li, 2022 | 2574 | 4608 | 1.36 | 1.09 | 1.7 | 0.01 |
| Dai, 2021 | 2549 | 4583 | 1.36 | 1.09 | 1.71 | 0.01 |
| Wang, 2022 | 2545 | 4436 | 1.35 | 1.07 | 1.7 | 0.01 |
| liang, 2022 | 2584 | 4627 | 1.33 | 1.07 | 1.65 | 0.01 |
| Liu 2012 | 2615 | 4624 | 1.34 | 1.07 | 1.68 | 0.01 |
| Vardakas, 2015 | 2567 | 4656 | 1.32 | 1.06 | 1.64 | 0.01 |
| Zheng, 2017 | 2609 | 4657 | 1.34 | 1.07 | 1.66 | 0.01 |

# **Table S20 Sensitivity analysis for associations between Diabetes mellitus and CRKP infections in enrolled studies**

| Removed_Study | Sample_Size_Case | Sample_Size_Control | OR_Random | Lower_CI_Random | Upper_CI_Random | P_Value_Random |
| --- | --- | --- | --- | --- | --- | --- |
| None(n=39) | 3653 | 6862 | 1.08 | 0.92 | 1.28 | 0.35 |
| Falagas, 2007 | 3600 | 6809 | 1.1 | 0.93 | 1.3 | 0.27 |
| Patel, 2008 | 3554 | 6763 | 1.07 | 0.91 | 1.28 | 0.41 |
| Wu, 2011 | 3614 | 6784 | 1.09 | 0.91 | 1.29 | 0.35 |
| Simkins, 2014 | 3640 | 6823 | 1.09 | 0.92 | 1.29 | 0.32 |
| Candevir, 2015 | 3606 | 6811 | 1.09 | 0.92 | 1.29 | 0.33 |
| Pereira, 2015 | 3633 | 6826 | 1.08 | 0.91 | 1.29 | 0.36 |
| Pouch, 2015 | 3633 | 6782 | 1.06 | 0.9 | 1.26 | 0.47 |
| Hu, 2016 | 3588 | 6797 | 1.08 | 0.91 | 1.28 | 0.40 |
| Wang, 2018 | 3605 | 6814 | 1.08 | 0.91 | 1.28 | 0.38 |
| Zheng, 2018 | 3594 | 6632 | 1.11 | 0.94 | 1.31 | 0.22 |
| Cienfuegos, 2019 | 3604 | 6573 | 1.1 | 0.93 | 1.3 | 0.28 |
| Liu, 2019 | 3633 | 6793 | 1.08 | 0.91 | 1.28 | 0.38 |
| Zhang, 2021 | 3511 | 6720 | 1.09 | 0.91 | 1.29 | 0.36 |
| Zhang, 2021 | 3556 | 6821 | 1.07 | 0.9 | 1.26 | 0.46 |
| Zuo, 2020 | 3579 | 6788 | 1.08 | 0.91 | 1.29 | 0.36 |
| Cao, 2022 | 3569 | 6775 | 1.09 | 0.92 | 1.3 | 0.31 |
| Chen, 2022 | 3441 | 6368 | 1.1 | 0.93 | 1.31 | 0.27 |
| Chen, 2022 | 3624 | 6639 | 1.07 | 0.9 | 1.27 | 0.43 |
| Lou, 2022 | 3231 | 5914 | 1.1 | 0.92 | 1.31 | 0.29 |
| Panda, 2022 | 3545 | 6746 | 1.06 | 0.9 | 1.25 | 0.49 |
| Arslan, 2023 | 3609 | 6786 | 1.06 | 0.9 | 1.26 | 0.47 |
| Çölkesen, 2023 | 3521 | 6712 | 1.06 | 0.9 | 1.26 | 0.47 |
| Huang, 2023 | 3159 | 5505 | 1.09 | 0.92 | 1.3 | 0.33 |
| Wang, 2023 | 3479 | 6643 | 1.1 | 0.92 | 1.3 | 0.30 |
| Cheng, 2024 | 3603 | 6778 | 1.09 | 0.92 | 1.3 | 0.31 |
| Radu, 2024 | 3591 | 6726 | 1.08 | 0.91 | 1.28 | 0.40 |
| Hussein, 2009 | 3565 | 6489 | 1.1 | 0.92 | 1.3 | 0.30 |
| zheng, 2017 | 3602 | 6811 | 1.07 | 0.9 | 1.26 | 0.45 |
| Gupta, 2020 | 3599 | 6836 | 1.06 | 0.9 | 1.25 | 0.50 |
| Li, 2020 | 3489 | 6534 | 1.12 | 0.96 | 1.31 | 0.16 |
| Li, 2022 | 3587 | 6796 | 1.09 | 0.92 | 1.29 | 0.33 |
| Dai, 2021 | 3562 | 6771 | 1.1 | 0.93 | 1.3 | 0.28 |
| Wang, 2022 | 3558 | 6624 | 1.05 | 0.89 | 1.24 | 0.53 |
| Wu, 2022 | 3585 | 6716 | 1.11 | 0.94 | 1.31 | 0.22 |
| liang, 2022 | 3597 | 6815 | 1.1 | 0.92 | 1.3 | 0.29 |
| Shilo, 2013 | 3518 | 6735 | 1.08 | 0.91 | 1.29 | 0.37 |
| Liu 2012 | 3628 | 6812 | 1.09 | 0.92 | 1.29 | 0.33 |
| Vardakas, 2015 | 3580 | 6844 | 1.07 | 0.91 | 1.27 | 0.41 |
| Zheng, 2017 | 3622 | 6845 | 1.09 | 0.92 | 1.29 | 0.34 |

# **Table S21 Sensitivity analysis for associations between Kidney disease and CRKP infections in enrolled studies**

| Removed_Study | Sample_Size_Case | Sample_Size_Control | OR_Random | Lower_CI_Random | Upper_CI_Random | P_Value_Random |
| --- | --- | --- | --- | --- | --- | --- |
| None(n=31) | 2695 | 4905 | 1.47 | 1.2 | 1.8 | <0.01 |
| Falagas, 2007 | 2642 | 4852 | 1.48 | 1.2 | 1.83 | <0.01 |
| Falagas, 2007 | 2642 | 4852 | 1.48 | 1.2 | 1.82 | <0.01 |
| Candevir, 2015 | 2648 | 4854 | 1.46 | 1.19 | 1.79 | <0.01 |
| Hu, 2016 | 2630 | 4840 | 1.45 | 1.18 | 1.78 | <0.01 |
| Zheng, 2018 | 2636 | 4675 | 1.45 | 1.18 | 1.79 | <0.01 |
| Cienfuegos, 2019 | 2646 | 4616 | 1.51 | 1.24 | 1.85 | <0.01 |
| Chang, 2019 | 2649 | 4666 | 1.47 | 1.19 | 1.83 | <0.01 |
| Cao, 2022 | 2611 | 4818 | 1.42 | 1.16 | 1.74 | <0.01 |
| Chen, 2022 | 2483 | 4411 | 1.5 | 1.2 | 1.86 | <0.01 |
| Lou, 2022 | 2273 | 3957 | 1.48 | 1.19 | 1.84 | <0.01 |
| Panda, 2022 | 2587 | 4789 | 1.51 | 1.22 | 1.86 | <0.01 |
| Çölkesen, 2023 | 2563 | 4755 | 1.46 | 1.18 | 1.81 | <0.01 |
| Wang, 2023 | 2521 | 4686 | 1.47 | 1.19 | 1.82 | <0.01 |
| Cheng, 2024 | 2645 | 4821 | 1.46 | 1.18 | 1.81 | <0.01 |
| Radu, 2024 | 2633 | 4769 | 1.45 | 1.17 | 1.79 | <0.01 |
| Li, 2020 | 2531 | 4577 | 1.43 | 1.16 | 1.76 | <0.01 |
| Liu, 2022 | 2621 | 4532 | 1.45 | 1.17 | 1.8 | <0.01 |
| Li, 2022 | 2629 | 4839 | 1.52 | 1.25 | 1.84 | <0.01 |
| Dai, 2021 | 2604 | 4814 | 1.46 | 1.18 | 1.8 | <0.01 |
| Wu, 2022 | 2627 | 4759 | 1.51 | 1.24 | 1.84 | <0.01 |
| liang, 2022 | 2639 | 4858 | 1.45 | 1.18 | 1.79 | <0.01 |
| Shilo, 2013 | 2560 | 4778 | 1.52 | 1.25 | 1.85 | <0.01 |
| Mouloudi, 2010 | 2676 | 4883 | 1.48 | 1.2 | 1.82 | <0.01 |
| Orsi 2011 | 2657 | 4843 | 1.43 | 1.17 | 1.75 | <0.01 |
| Orsi 2011 | 2657 | 4843 | 1.48 | 1.2 | 1.83 | <0.01 |
| Liu 2012 | 2670 | 4855 | 1.43 | 1.17 | 1.76 | <0.01 |
| Liu 2012 | 2670 | 4855 | 1.44 | 1.17 | 1.76 | <0.01 |
| Hussein, 2013 | 2592 | 4691 | 1.46 | 1.17 | 1.82 | <0.01 |
| Vardakas, 2015 | 2622 | 4887 | 1.46 | 1.19 | 1.8 | <0.01 |
| Vardakas, 2015 | 2622 | 4887 | 1.46 | 1.19 | 1.79 | <0.01 |
| Zheng, 2017 | 2664 | 4888 | 1.47 | 1.2 | 1.81 | <0.01 |

# **Table S22 Sensitivity analysis for associations between chronic lung disease and CRKP infections in enrolled studies**

| Removed_Study | Sample_Size_Case | Sample_Size_Control | OR_Random | Lower_CI_Random | Upper_CI_Random | P_Value_Random |
| --- | --- | --- | --- | --- | --- | --- |
| None(n=23) | 2043 | 3769 | 1.28 | 0.93 | 1.75 | 0.13 |
| Falagas, 2007 | 1990 | 3716 | 1.24 | 0.9 | 1.72 | 0.19 |
| Hu, 2016 | 1978 | 3704 | 1.31 | 0.94 | 1.82 | 0.11 |
| Wang, 2018 | 1995 | 3721 | 1.29 | 0.93 | 1.79 | 0.13 |
| Xiao, 2018 | 1908 | 3476 | 1.17 | 0.9 | 1.52 | 0.25 |
| Zheng, 2018 | 1984 | 3539 | 1.25 | 0.91 | 1.73 | 0.17 |
| Cienfuegos, 2019 | 1994 | 3480 | 1.31 | 0.94 | 1.81 | 0.11 |
| Chang, 2019 | 1997 | 3530 | 1.3 | 0.94 | 1.8 | 0.11 |
| Zhang, 2021 | 1901 | 3627 | 1.32 | 0.95 | 1.82 | 0.10 |
| Zhang, 2021 | 1946 | 3728 | 1.28 | 0.92 | 1.79 | 0.15 |
| Zuo, 2020 | 1969 | 3695 | 1.26 | 0.91 | 1.76 | 0.16 |
| Chen, 2022 | 1831 | 3275 | 1.27 | 0.91 | 1.78 | 0.16 |
| Panda, 2022 | 1935 | 3653 | 1.28 | 0.92 | 1.79 | 0.15 |
| Çölkesen, 2023 | 1911 | 3619 | 1.27 | 0.91 | 1.78 | 0.16 |
| Cheng, 2024 | 1993 | 3685 | 1.24 | 0.9 | 1.72 | 0.19 |
| Hussein, 2009 | 1955 | 3396 | 1.33 | 0.97 | 1.83 | 0.08 |
| zheng, 2017 | 1992 | 3718 | 1.36 | 1.02 | 1.82 | 0.04* |
| Li, 2020 | 1879 | 3441 | 1.22 | 0.89 | 1.67 | 0.21 |
| Dai, 2021 | 1952 | 3678 | 1.23 | 0.9 | 1.68 | 0.19 |
| Wang, 2022 | 1948 | 3531 | 1.28 | 0.91 | 1.79 | 0.15 |
| Wu, 2022 | 1975 | 3623 | 1.33 | 0.96 | 1.83 | 0.08 |
| liang, 2022 | 1987 | 3722 | 1.31 | 0.95 | 1.79 | 0.10 |
| Shilo, 2013 | 1908 | 3642 | 1.32 | 0.95 | 1.83 | 0.10 |
| Liu 2012 | 2018 | 3719 | 1.21 | 0.9 | 1.64 | 0.21 |

# **Table S23 Sensitivity analysis for associations between mental illness and CRKP infections in enrolled studies**

| Removed_Study | Sample_Size_Case | Sample_Size_Control | OR_Random | Lower_CI_Random | Upper_CI_Random | P_Value_Random |
| --- | --- | --- | --- | --- | --- | --- |
| None(n=13) | 1176 | 1375 | 1.15 | 0.91 | 1.45 | 0.23 |
| Falagas, 2007 | 1123 | 1322 | 1.15 | 0.9 | 1.48 | 0.26 |
| Hu, 2016 | 1111 | 1310 | 1.13 | 0.88 | 1.44 | 0.34 |
| Wang, 2018 | 1128 | 1327 | 1.09 | 0.87 | 1.36 | 0.45 |
| Zhang, 2021 | 1034 | 1233 | 1.22 | 0.95 | 1.55 | 0.11 |
| Zhang, 2021 | 1079 | 1334 | 1.14 | 0.89 | 1.46 | 0.31 |
| Zuo, 2020 | 1102 | 1301 | 1.22 | 0.99 | 1.5 | 0.07 |
| Çölkesen, 2023 | 1044 | 1225 | 1.18 | 0.9 | 1.54 | 0.23 |
| Li, 2023 | 908 | 956 | 1.18 | 0.91 | 1.52 | 0.21 |
| zheng, 2017 | 1125 | 1324 | 1.15 | 0.9 | 1.47 | 0.26 |
| Gupta, 2020 | 1122 | 1349 | 1.15 | 0.91 | 1.46 | 0.24 |
| Wang, 2022 | 1081 | 1137 | 1.11 | 0.88 | 1.42 | 0.38 |
| Liu 2012 | 1151 | 1325 | 1.12 | 0.89 | 1.42 | 0.34 |
| Vardakas, 2015 | 1104 | 1357 | 1.13 | 0.9 | 1.43 | 0.29 |

# **Table S24 Sensitivity analysis for associations between Respiratory system disease and CRKP infections in enrolled studies**

| Removed_Study | Sample_Size_Case | Sample_Size_Control | OR_Random | Lower_CI_Random | Upper_CI_Random | P_Value_Random |
| --- | --- | --- | --- | --- | --- | --- |
| None(n=7) | 1188 | 2530 | 2.67 | 2.14 | 3.34 | <0.01 |
| Wu, 2011 | 1149 | 2452 | 2.81 | 2.3 | 3.44 | <0.01 |
| Li, 2023 | 920 | 2111 | 2.5 | 2.04 | 3.07 | <0.01 |
| Li, 2022 | 1122 | 2464 | 2.73 | 2.04 | 3.65 | <0.01 |
| Vardakas, 2015 | 1115 | 2512 | 2.69 | 2.08 | 3.46 | <0.01 |
| Huang, 2023 | 694 | 1173 | 2.62 | 1.77 | 3.87 | <0.01 |
| Wang, 2023 | 1014 | 2311 | 2.57 | 1.89 | 3.49 | <0.01 |
| Liu, 2022 | 1114 | 2157 | 2.64 | 1.89 | 3.69 | <0.01 |

# **Table S25 Sensitivity analysis for associations between Hypertension and CRKP infections in enrolled studies**

| Removed_Study | Sample_Size_Case | Sample_Size_Control | OR_Random | Lower_CI_Random | Upper_CI_Random | P_Value_Random |
| --- | --- | --- | --- | --- | --- | --- |
| None(n=14) | 1395 | 2853 | 1.36 | 0.98 | 1.89 | 0.07 |
| Pouch, 2015 | 1375 | 2773 | 1.36 | 0.96 | 1.93 | 0.08 |
| Zheng, 2018 | 1336 | 2623 | 1.34 | 0.94 | 1.91 | 0.11 |
| Zhang, 2021 | 1298 | 2812 | 1.32 | 0.93 | 1.87 | 0.11 |
| Zuo, 2020 | 1321 | 2779 | 1.37 | 0.95 | 1.96 | 0.09 |
| Cao, 2022 | 1311 | 2766 | 1.3 | 0.92 | 1.83 | 0.13 |
| Chen, 2022 | 1366 | 2630 | 1.21 | 0.93 | 1.58 | 0.16 |
| Panda, 2022 | 1287 | 2737 | 1.39 | 0.96 | 1.99 | 0.08 |
| Huang, 2023 | 901 | 1496 | 1.44 | 1.02 | 2.05 | 0.04* |
| Radu, 2024 | 1333 | 2717 | 1.47 | 1.11 | 1.96 | 0.01* |
| zheng, 2017 | 1344 | 2802 | 1.4 | 0.98 | 1.99 | 0.06 |
| Li, 2020 | 1231 | 2525 | 1.35 | 0.94 | 1.95 | 0.10 |
| Li, 2022 | 1329 | 2787 | 1.4 | 0.98 | 1.99 | 0.06 |
| liang, 2022 | 1339 | 2806 | 1.34 | 0.96 | 1.86 | 0.08 |
| Zheng, 2017 | 1364 | 2836 | 1.4 | 0.99 | 1.97 | 0.06 |

# **Table S26 Sensitivity analysis for associations between liver disease and CRKP infections in enrolled studies**

| Removed_Study | Sample_Size_Case | Sample_Size_Control | OR_Random | Lower_CI_Random | Upper_CI_Random | P_Value_Random |
| --- | --- | --- | --- | --- | --- | --- |
| None(n=16) | 1497 | 2582 | 1.16 | 0.85 | 1.59 | 0.35 |
| Falagas, 2007 | 1444 | 2529 | 1.19 | 0.86 | 1.65 | 0.30 |
| Patel, 2008 | 1398 | 2483 | 1.13 | 0.81 | 1.58 | 0.48 |
| Xiao, 2018 | 1362 | 2289 | 1.25 | 0.9 | 1.73 | 0.18 |
| Zheng, 2018 | 1438 | 2352 | 1.13 | 0.82 | 1.55 | 0.46 |
| Liu, 2019 | 1477 | 2513 | 1.09 | 0.81 | 1.47 | 0.57 |
| Hsu, 2021 | 1461 | 2510 | 1.09 | 0.8 | 1.48 | 0.58 |
| Zhang, 2021 | 1355 | 2440 | 1.22 | 0.87 | 1.71 | 0.25 |
| Zhang, 2021 | 1400 | 2541 | 1.19 | 0.84 | 1.68 | 0.33 |
| Lou, 2022 | 1075 | 1634 | 1.25 | 0.89 | 1.74 | 0.19 |
| zheng, 2017 | 1446 | 2531 | 1.13 | 0.82 | 1.56 | 0.44 |
| Wang, 2022 | 1402 | 2344 | 1.23 | 0.87 | 1.73 | 0.24 |
| liang, 2022 | 1441 | 2535 | 1.18 | 0.86 | 1.62 | 0.31 |
| Liu 2012 | 1472 | 2532 | 1.17 | 0.84 | 1.64 | 0.35 |
| Hussein, 2013 | 1394 | 2368 | 1.04 | 0.78 | 1.39 | 0.77 |
| Vardakas, 2015 | 1424 | 2564 | 1.15 | 0.84 | 1.58 | 0.39 |
| Zheng, 2017 | 1466 | 2565 | 1.17 | 0.84 | 1.62 | 0.35 |

# **Table S27 Sensitivity analysis for associations between β-lactamase inhibitor and CRKP infections in enrolled studies**

| Removed_Study | Sample_Size_Case | Sample_Size_Control | OR_Random | Lower_CI_Random | Upper_CI_Random | P_Value_Random |
| --- | --- | --- | --- | --- | --- | --- |
| None(n=24) | 2793 | 5763 | 2.24 | 1.8 | 2.79 | <0.01 |
| Patel, 2008 | 2694 | 5664 | 2.24 | 1.78 | 2.82 | <0.01 |
| Simkins, 2014 | 2780 | 5724 | 2.2 | 1.77 | 2.75 | <0.01 |
| Hu, 2016 | 2728 | 5698 | 2.33 | 1.88 | 2.88 | <0.01 |
| Wang, 2018 | 2745 | 5715 | 2.19 | 1.76 | 2.73 | <0.01 |
| Xiao, 2018 | 2658 | 5470 | 2.24 | 1.78 | 2.83 | <0.01 |
| Zheng, 2018 | 2734 | 5533 | 2.24 | 1.78 | 2.82 | <0.01 |
| Liu, 2019 | 2773 | 5694 | 2.23 | 1.79 | 2.78 | <0.01 |
| Chang, 2019 | 2747 | 5524 | 2.25 | 1.79 | 2.83 | <0.01 |
| Xiao, 2020 | 2689 | 5496 | 2.21 | 1.76 | 2.77 | <0.01 |
| Zhang, 2021 | 2651 | 5621 | 2.26 | 1.79 | 2.84 | <0.01 |
| Zhang, 2021 | 2696 | 5722 | 2.29 | 1.83 | 2.87 | <0.01 |
| Zuo, 2020 | 2719 | 5689 | 2.29 | 1.82 | 2.87 | <0.01 |
| Chen, 2022 | 2581 | 5269 | 2.32 | 1.86 | 2.9 | <0.01 |
| Chen, 2022 | 2764 | 5540 | 2.21 | 1.77 | 2.76 | <0.01 |
| Lou, 2022 | 2371 | 4815 | 2.33 | 1.89 | 2.89 | <0.01 |
| Huang, 2023 | 2299 | 4406 | 2.32 | 1.85 | 2.9 | <0.01 |
| Wang, 2023 | 2619 | 5544 | 2.31 | 1.85 | 2.89 | <0.01 |
| Cheng, 2024 | 2743 | 5679 | 2.21 | 1.76 | 2.76 | <0.01 |
| Li, 2020 | 2629 | 5435 | 2.18 | 1.74 | 2.72 | <0.01 |
| Li, 2022 | 2727 | 5697 | 2.24 | 1.78 | 2.81 | <0.01 |
| Dai, 2021 | 2702 | 5672 | 2.24 | 1.79 | 2.81 | <0.01 |
| Kritsotakis, 2011 | 2697 | 5612 | 2.2 | 1.75 | 2.75 | <0.01 |
| Wu, 2022 | 2725 | 5617 | 2.13 | 1.73 | 2.63 | <0.01 |
| Liu, 2012 | 2768 | 5713 | 2.2 | 1.77 | 2.75 | <0.01 |

# **Table S28 Sensitivity analysis for associations between Aminoglycoside and CRKP infections in enrolled studies**

| Removed_Study | Sample_Size_Case | Sample_Size_Control | OR_Random | Lower_CI_Random | Upper_CI_Random | P_Value_Random |
| --- | --- | --- | --- | --- | --- | --- |
| None(n=28) | 2943 | 6386 | 2.3 | 1.68 | 3.15 | <0.01 |
| Falagas, 2007 | 2899 | 6342 | 2.28 | 1.65 | 3.14 | <0.01 |
| Patel, 2008 | 2844 | 6287 | 2.23 | 1.63 | 3.07 | <0.01 |
| Wu, 2011 | 2904 | 6308 | 2.39 | 1.73 | 3.3 | <0.01 |
| Simkins, 2014 | 2930 | 6347 | 2.32 | 1.68 | 3.2 | <0.01 |
| Hu, 2016 | 2878 | 6321 | 2.31 | 1.68 | 3.18 | <0.01 |
| Wang, 2018 | 2895 | 6338 | 2.34 | 1.7 | 3.22 | <0.01 |
| Zheng, 2018 | 2884 | 6156 | 2.2 | 1.62 | 2.98 | <0.01 |
| Cienfuegos, 2019 | 2894 | 6097 | 2.24 | 1.63 | 3.09 | <0.01 |
| Liu, 2019 | 2923 | 6317 | 2.24 | 1.64 | 3.07 | <0.01 |
| Xiao, 2020 | 2839 | 6119 | 2.19 | 1.62 | 2.98 | <0.01 |
| Zhang, 2021 | 2846 | 6345 | 2.35 | 1.71 | 3.24 | <0.01 |
| Zuo, 2020 | 2869 | 6312 | 2.27 | 1.65 | 3.12 | <0.01 |
| Chen, 2022 | 2731 | 5892 | 2.34 | 1.68 | 3.26 | <0.01 |
| Lou, 2022 | 2521 | 5438 | 2.14 | 1.57 | 2.9 | <0.01 |
| Huang, 2023 | 2449 | 5029 | 2.4 | 1.73 | 3.34 | <0.01 |
| Wang, 2023 | 2769 | 6167 | 2.44 | 1.8 | 3.32 | <0.01 |
| Hussein, 2009 | 2855 | 6013 | 2.39 | 1.73 | 3.31 | <0.01 |
| zheng, 2017 | 2892 | 6335 | 2.39 | 1.73 | 3.29 | <0.01 |
| Li, 2020 | 2779 | 6058 | 2.27 | 1.64 | 3.14 | <0.01 |
| Liu, 2022 | 2869 | 6013 | 2.3 | 1.66 | 3.19 | <0.01 |
| Li, 2022 | 2877 | 6320 | 2.28 | 1.66 | 3.14 | <0.01 |
| Wang, 2022 | 2848 | 6148 | 2.37 | 1.7 | 3.3 | <0.01 |
| Kritsotakis 2011 | 2847 | 6235 | 2.29 | 1.65 | 3.18 | <0.01 |
| Wu, 2022 | 2875 | 6240 | 2.17 | 1.6 | 2.94 | <0.01 |
| Liu 2012 | 2918 | 6336 | 2.36 | 1.72 | 3.25 | <0.01 |
| Hussein, 2013 | 2840 | 6172 | 2.28 | 1.64 | 3.17 | <0.01 |
| Vardakas, 2015 | 2874 | 6368 | 2.4 | 1.75 | 3.29 | <0.01 |
| Zheng, 2017 | 2912 | 6369 | 2.32 | 1.68 | 3.19 | <0.01 |

# **Table S29 Sensitivity analysis for associations between Macrolides and CRKP infections in enrolled studies**

| Removed_Study | Sample_Size_Case | Sample_Size_Control | OR_Random | Lower_CI_Random | Upper_CI_Random | P_Value_Random |
| --- | --- | --- | --- | --- | --- | --- |
| None(n=6) | 872 | 2114 | 2.76 | 1.22 | 6.23 | 0.01 |
| Wu, 2011 | 833 | 2036 | 2.53 | 1.07 | 5.98 | 0.03 |
| Zhang, 2021 | 775 | 2073 | 3.05 | 1.12 | 8.32 | 0.03 |
| Huang, 2023 | 378 | 757 | 2.61 | 0.95 | 7.17 | 0.06 |
| Hussein, 2009 | 784 | 1741 | 3.66 | 1.5 | 8.91 | <0.01 |
| zheng, 2017 | 821 | 2063 | 3.11 | 1.35 | 7.16 | 0.01 |
| Hussein, 2013 | 769 | 1900 | 1.7 | 1.02 | 2.86 | 0.04 |

# **Table S30 Sensitivity analysis for associations between Polymyxin and CRKP infections in enrolled studies**

| Removed_Study | Sample_Size_Case | Sample_Size_Control | OR_Random | Lower_CI_Random | Upper_CI_Random | P_Value_Random |
| --- | --- | --- | --- | --- | --- | --- |
| None(n=8) | 963 | 1854 | 4.25 | 2.02 | 8.95 | <0.01 |
| Hussein, 2009 | 875 | 1481 | 4.04 | 1.72 | 9.47 | <0.01 |
| Hussein, 2013 | 860 | 1640 | 3.34 | 1.6 | 6.95 | <0.01 |
| Vardakas, 2015 | 894 | 1836 | 5.34 | 2.55 | 11.18 | <0.01 |
| Çölkesen,, 2023 | 831 | 1704 | 5.3 | 2.43 | 11.57 | <0.01 |
| Lou, 2022 | 541 | 906 | 4.06 | 1.78 | 9.26 | <0.01 |
| Wu, 2011 | 924 | 1776 | 4.33 | 1.82 | 10.35 | <0.01 |
| liang, 2022 | 907 | 1807 | 4.07 | 1.87 | 8.86 | <0.01 |
| Gupta, 2020 | 909 | 1828 | 3.91 | 1.82 | 8.39 | <0.01 |

# **Table S31 Sensitivity analysis for associations between Linezolid and CRKP infections in enrolled studies**

| Removed_Study | Sample_Size_Case | Sample_Size_Control | OR_Random | Lower_CI_Random | Upper_CI_Random | P_Value_Random |
| --- | --- | --- | --- | --- | --- | --- |
| None(n=7) | 1552 | 3335 | 2.38 | 1.4 | 4.06 | <0.01 |
| Zheng, 2017 | 1521 | 3318 | 2.21 | 1.3 | 3.76 | <0.01 |
| Li, 2020 | 1388 | 3007 | 1.97 | 1.28 | 3.02 | <0.01 |
| Zhang, 2021 | 1455 | 3294 | 2.59 | 1.4 | 4.78 | <0.01 |
| Chen, 2022 | 1340 | 2841 | 2.71 | 1.5 | 4.9 | <0.01 |
| Lou, 2022 | 1130 | 2387 | 2.28 | 1.19 | 4.36 | 0.01 |
| Çölkesen, 2023 | 1420 | 3185 | 2.75 | 1.56 | 4.86 | <0.01 |
| Huang, 2023 | 1058 | 1978 | 2.41 | 1.22 | 4.78 | 0.01 |

# **Table S32 Sensitivity analysis for associations between Fluoroquinolone and CRKP infections in enrolled studies**

| Removed_Study | Sample_Size_Case | Sample_Size_Control | OR_Random | Lower_CI_Random | Upper_CI_Random | P_Value_Random |
| --- | --- | --- | --- | --- | --- | --- |
| None(n=36) | 3653 | 7634 | 2.37 | 1.81 | 3.11 | <0.01 |
| Falagas, 2007 | 3609 | 7590 | 2.32 | 1.76 | 3.06 | <0.01 |
| Patel,2008 | 3554 | 7535 | 2.39 | 1.81 | 3.17 | <0.01 |
| Wu,2011 | 3614 | 7556 | 2.42 | 1.83 | 3.19 | <0.01 |
| Simkins,2014 | 3640 | 7595 | 2.3 | 1.76 | 3.01 | <0.01 |
| Hu,2016 | 3588 | 7569 | 2.38 | 1.81 | 3.15 | <0.01 |
| Wang,2018 | 3605 | 7586 | 2.36 | 1.79 | 3.12 | <0.01 |
| Xiao,2018 | 3518 | 7341 | 2.36 | 1.78 | 3.13 | <0.01 |
| Zheng,2018 | 3594 | 7404 | 2.4 | 1.81 | 3.17 | <0.01 |
| Cienfuegos,2019 | 3604 | 7345 | 2.36 | 1.78 | 3.12 | <0.01 |
| Liu,2019 | 3633 | 7565 | 2.37 | 1.8 | 3.12 | <0.01 |
| Chang,2019 | 3607 | 7395 | 2.32 | 1.76 | 3.07 | <0.01 |
| Xiao,2020 | 3549 | 7367 | 2.37 | 1.79 | 3.14 | <0.01 |
| Zhang,2021 | 3511 | 7492 | 2.36 | 1.79 | 3.13 | <0.01 |
| Zhang,2021 | 3556 | 7593 | 2.34 | 1.78 | 3.09 | <0.01 |
| Zuo,2020 | 3579 | 7560 | 2.35 | 1.78 | 3.11 | <0.01 |
| Chen,2022 | 3441 | 7140 | 2.43 | 1.84 | 3.21 | <0.01 |
| Chen,2022 | 3624 | 7411 | 2.39 | 1.81 | 3.16 | <0.01 |
| Lou,2022 | 3231 | 6686 | 2.37 | 1.79 | 3.14 | <0.01 |
| Çölkesen ,2023 | 3521 | 7484 | 2.11 | 1.75 | 2.54 | <0.01 |
| Huang,2023 | 3159 | 6277 | 2.49 | 1.91 | 3.24 | <0.01 |
| Wang,2023 | 3479 | 7415 | 2.4 | 1.81 | 3.18 | <0.01 |
| Cheng,2024 | 3603 | 7550 | 2.38 | 1.8 | 3.15 | <0.01 |
| Hussein,2009 | 3565 | 7261 | 2.36 | 1.78 | 3.13 | <0.01 |
| zheng,2017 | 3602 | 7583 | 2.43 | 1.85 | 3.21 | <0.01 |
| Gupta,2020 | 3568 | 7608 | 2.39 | 1.81 | 3.16 | <0.01 |
| Li,2020 | 3489 | 7306 | 2.36 | 1.78 | 3.13 | <0.01 |
| Liu,2022 | 3579 | 7261 | 2.36 | 1.78 | 3.13 | <0.01 |
| Li,2022 | 3587 | 7568 | 2.44 | 1.86 | 3.21 | <0.01 |
| Dai,2021 | 3562 | 7543 | 2.4 | 1.82 | 3.17 | <0.01 |
| Wang,2022 | 3558 | 7396 | 2.39 | 1.8 | 3.16 | <0.01 |
| Kritsotakis, 2011 | 3557 | 7483 | 2.34 | 1.77 | 3.09 | <0.01 |
| Wu,2022 | 3585 | 7488 | 2.4 | 1.81 | 3.17 | <0.01 |
| Liu,2012 | 3628 | 7584 | 2.38 | 1.8 | 3.15 | <0.01 |
| Hussein,2013 | 3550 | 7420 | 2.34 | 1.77 | 3.1 | <0.01 |
| Vardakas,2015 | 3584 | 7616 | 2.44 | 1.86 | 3.19 | <0.01 |
| Zheng,2017 | 3622 | 7617 | 2.38 | 1.81 | 3.13 | <0.01 |

# **Table S33 Sensitivity analysis for associations between Tigecycline and CRKP infections in enrolled studies**

| Removed_Study | Sample_Size_Case | Sample_Size_Control | OR_Random | Lower_CI_Random | Upper_CI_Random | P_Value_Random |
| --- | --- | --- | --- | --- | --- | --- |
| None(n=15) | 2084 | 4405 | 5.97 | 3.8 | 9.38 | <0.01 |
| Xiao, 2018 | 1949 | 4112 | 5.92 | 3.62 | 9.69 | <0.01 |
| Zheng, 2018 | 2025 | 4175 | 5.86 | 3.6 | 9.54 | <0.01 |
| Liu, 2019 | 2064 | 4336 | 5.72 | 3.64 | 9 | <0.01 |
| Xiao, 2020 | 1980 | 4138 | 5.56 | 3.48 | 8.9 | <0.01 |
| Zuo, 2020 | 2010 | 4331 | 6.17 | 3.81 | 10 | <0.01 |
| Chen, 2022 | 1872 | 3911 | 7.04 | 4.88 | 10.15 | <0.01 |
| Lou, 2022 | 1662 | 3457 | 5.46 | 3.43 | 8.69 | <0.01 |
| Huang, 2023 | 1590 | 3048 | 6.38 | 3.91 | 10.42 | <0.01 |
| Gupta, 2020 | 1999 | 4379 | 6.09 | 3.85 | 9.65 | <0.01 |
| Li, 2020 | 1920 | 4077 | 5.7 | 3.52 | 9.25 | <0.01 |
| Dai, 2021 | 1993 | 4314 | 6.01 | 3.74 | 9.67 | <0.01 |
| Wu, 2022 | 2016 | 4259 | 5.72 | 3.56 | 9.19 | <0.01 |
| liang, 2022 | 2028 | 4358 | 5.82 | 3.66 | 9.25 | <0.01 |
| Vardakas, 2015 | 2015 | 4387 | 6.36 | 4.01 | 10.09 | <0.01 |
| Zheng, 2017 | 2053 | 4388 | 5.98 | 3.77 | 9.48 | <0.01 |

# **Table S34 Sensitivity analysis for associations between Sulfanilamides and CRKP infections in enrolled studies**

| Removed_Study | Sample_Size_Case | Sample_Size_Control | OR_Random | Lower_CI_Random | Upper_CI_Random | P_Value_Random |
| --- | --- | --- | --- | --- | --- | --- |
| None(n=3) | 168 | 161 | 1.28 | 0.37 | 4.37 | 0.70 |
| Zheng, 2017 | 117 | 110 | 1.46 | 0.19 | 11.26 | 0.72 |
| Liu, 2019 | 148 | 92 | 3.38 | 0.53 | 21.72 | 0.20 |
| Zhang, 2021 | 71 | 120 | 0.84 | 0.33 | 2.12 | 0.71 |

# **Table S35 Sensitivity analysis for associations between Antifungal agents and CRKP infections in enrolled studies**

| Removed_Study | Sample_Size_Case | Sample_Size_Control | OR_Random | Lower_CI_Random | Upper_CI_Random | P_Value_Random |
| --- | --- | --- | --- | --- | --- | --- |
| None(n=10) | 1510 | 3396 | 3.07 | 2.09 | 4.53 | <0.01 |
| Wang, 2018 | 1462 | 3348 | 3.28 | 2.23 | 4.83 | <0.01 |
| Chang, 2019 | 1464 | 3157 | 2.73 | 1.97 | 3.77 | <0.01 |
| Zhang, 2021 | 1368 | 3254 | 3.28 | 2.19 | 4.91 | <0.01 |
| Zhang, 2021 | 1413 | 3355 | 3.18 | 2.08 | 4.84 | <0.01 |
| Lou, 2022 | 1088 | 2448 | 2.99 | 1.92 | 4.67 | <0.01 |
| Huang, 2023 | 1016 | 2039 | 3.37 | 2.28 | 5.01 | <0.01 |
| Cheng, 2024 | 1460 | 3312 | 2.93 | 1.94 | 4.42 | <0.01 |
| Liu, 2022 | 1436 | 3023 | 2.92 | 1.88 | 4.54 | <0.01 |
| Wu, 2022 | 1442 | 3250 | 2.94 | 1.92 | 4.5 | <0.01 |
| Vardakas, 2015 | 1441 | 3378 | 3.19 | 2.1 | 4.83 | <0.01 |

# **Table S36 Sensitivity analysis for associations between Clindamycin and CRKP infections in enrolled studies**

| Removed_Study | Sample_Size_Case | Sample_Size_Control | OR_Random | Lower_CI_Random | Upper_CI_Random | P_Value_Random |
| --- | --- | --- | --- | --- | --- | --- |
| None(n=3) | 563 | 1451 | 1.41 | 0.59 | 3.33 | 0.44 |
| Falagas, 2007 | 519 | 1407 | 1.11 | 0.78 | 1.58 | 0.55 |
| Liu, 2012 | 538 | 1401 | 1.98 | 0.38 | 10.33 | 0.42 |
| Huang, 2023 | 69 | 94 | 2.85 | 0.44 | 18.48 | 0.27 |

# **Table S37 Sensitivity analysis for associations between Penicillins and CRKP infections in enrolled studies**

| Removed_Study | Sample_Size_Case | Sample_Size_Control | OR_Random | Lower_CI_Random | Upper_CI_Random | P_Value_Random |
| --- | --- | --- | --- | --- | --- | --- |
| None(n=16) | 2031 | 4547 | 1.54 | 1.06 | 2.25 | 0.02 |
| Falagas, 2007 | 1987 | 4503 | 1.46 | 0.99 | 2.14 | 0.05 |
| Wu, 2011 | 1992 | 4469 | 1.49 | 1 | 2.21 | 0.05 |
| Candevir, 2015 | 1984 | 4496 | 1.49 | 1 | 2.21 | 0.05 |
| Cienfuegos, 2019 | 1982 | 4258 | 1.58 | 1.04 | 2.39 | 0.03 |
| Liu, 2019 | 2011 | 4478 | 1.45 | 1.02 | 2.07 | 0.04 |
| Chen, 2022 | 1819 | 4053 | 1.64 | 1.12 | 2.4 | 0.01 |
| Lou, 2022 | 1609 | 3599 | 1.55 | 1.01 | 2.38 | 0.05 |
| Çölkesen, ,2023 | 1899 | 4397 | 1.56 | 1.02 | 2.38 | 0.04 |
| Huang, 2023 | 1537 | 3190 | 1.65 | 1.16 | 2.36 | 0.01 |
| Hussein, 2009 | 1943 | 4174 | 1.52 | 1 | 2.33 | 0.05 |
| zheng, 2017 | 1980 | 4496 | 1.6 | 1.07 | 2.37 | 0.02 |
| Li, 2020 | 1867 | 4219 | 1.66 | 1.15 | 2.39 | 0.01 |
| Li, 2022 | 1965 | 4481 | 1.62 | 1.11 | 2.36 | 0.01 |
| Hussein, 2013 | 1928 | 4333 | 1.4 | 1.03 | 1.9 | 0.03 |
| Vardakas, 2015 | 1962 | 4529 | 1.54 | 1.03 | 2.32 | 0.04 |
| Zheng, 2017 | 2000 | 4530 | 1.52 | 1.02 | 2.26 | 0.04 |

# **Table S38 Sensitivity analysis for associations between Tetracyclines and CRKP infections in enrolled studies**

| Removed_Study | Sample_Size_Case | Sample_Size_Control | OR_Random | Lower_CI_Random | Upper_CI_Random | P_Value_Random |
| --- | --- | --- | --- | --- | --- | --- |
| None(n=3) | 322 | 311 | 1.04 | 0.22 | 4.81 | 0.96 |
| zheng, 2017 | 271 | 260 | 1.52 | 0.21 | 10.87 | 0.68 |
| Zhang, 2021 | 225 | 270 | 1.37 | 0.14 | 12.96 | 0.78 |
| Wang, 2023 | 148 | 92 | 0.45 | 0.15 | 1.3 | 0.14 |

# **Table S39 Sensitivity analysis for associations between Carbapenems and CRKP infections in enrolled studies**

| Removed_Study | Sample_Size_Case | Sample_Size_Control | OR_Random | Lower_CI_Random | Upper_CI_Random | P_Value_Random |
| --- | --- | --- | --- | --- | --- | --- |
| None(n=44) | 4310 | 8608 | 4.79 | 3.72 | 6.18 | <0.01 |
| Falagas, 2007 | 4266 | 8564 | 4.83 | 3.73 | 6.26 | <0.01 |
| Patel, 2008 | 4211 | 8509 | 4.65 | 3.62 | 5.98 | <0.01 |
| Wu, 2011 | 4271 | 8530 | 4.82 | 3.72 | 6.25 | <0.01 |
| Simkins, 2014 | 4297 | 8569 | 4.74 | 3.67 | 6.12 | <0.01 |
| Candevir 2015 | 4263 | 8557 | 4.83 | 3.73 | 6.26 | <0.01 |
| Hu, 2016 | 4245 | 8543 | 4.82 | 3.71 | 6.24 | <0.01 |
| Wang, 2018 | 4262 | 8560 | 4.76 | 3.68 | 6.15 | <0.01 |
| Xiao, 2018 | 4175 | 8315 | 4.72 | 3.64 | 6.11 | <0.01 |
| Zheng, 2018 | 4251 | 8378 | 4.7 | 3.64 | 6.09 | <0.01 |
| Cienfuegos 2019 | 4261 | 8319 | 4.73 | 3.65 | 6.12 | <0.01 |
| Liu, 2019 | 4290 | 8539 | 5.06 | 4.02 | 6.38 | <0.01 |
| Pan, 2019 | 4244 | 8476 | 4.75 | 3.67 | 6.15 | <0.01 |
| Chang, 2019 | 4264 | 8369 | 4.81 | 3.71 | 6.24 | <0.01 |
| Wu, 2020 | 4272 | 8578 | 4.84 | 3.74 | 6.27 | <0.01 |
| Xiao, 2020 | 4206 | 8341 | 4.77 | 3.67 | 6.19 | <0.01 |
| Hsu, 2021 | 4274 | 8536 | 4.69 | 3.64 | 6.06 | <0.01 |
| Zhang Hj 2021 | 4168 | 8466 | 4.84 | 3.73 | 6.27 | <0.01 |
| Zhang H 2021 | 4213 | 8567 | 4.82 | 3.72 | 6.24 | <0.01 |
| Zuo, 2020 | 4236 | 8534 | 4.81 | 3.71 | 6.24 | <0.01 |
| Cao, 2022 | 4226 | 8521 | 4.76 | 3.67 | 6.18 | <0.01 |
| Chen, 2022 | 4098 | 8114 | 5.01 | 3.92 | 6.39 | <0.01 |
| Chen, 2022 | 4281 | 8385 | 4.71 | 3.64 | 6.1 | <0.01 |
| Lou, 2022 | 3888 | 7660 | 4.76 | 3.66 | 6.18 | <0.01 |
| Çölkesen, 2023 | 4178 | 8458 | 4.64 | 3.61 | 5.96 | <0.01 |
| Huang, 2023 | 3816 | 7251 | 4.9 | 3.78 | 6.33 | <0.01 |
| Li, 2023 | 4042 | 8189 | 4.73 | 3.65 | 6.13 | <0.01 |
| Wang, 2023 | 4136 | 8389 | 4.68 | 3.62 | 6.04 | <0.01 |
| Cheng, 2024 | 4260 | 8524 | 4.81 | 3.71 | 6.23 | <0.01 |
| Radu, 2024 | 4248 | 8472 | 4.74 | 3.68 | 6.12 | <0.01 |
| Hussein, 2009 | 4222 | 8235 | 4.84 | 3.74 | 6.28 | <0.01 |
| zheng B 2017 | 4259 | 8557 | 4.85 | 3.74 | 6.28 | <0.01 |
| Gupta, 2020 | 4225 | 8582 | 4.8 | 3.7 | 6.22 | <0.01 |
| Li, 2020 | 4146 | 8280 | 4.71 | 3.64 | 6.09 | <0.01 |
| Liu, 2022 | 4236 | 8235 | 4.73 | 3.65 | 6.13 | <0.01 |
| Li, 2022 | 4244 | 8542 | 5 | 3.91 | 6.38 | <0.01 |
| Dai, 2021 | 4219 | 8517 | 4.76 | 3.67 | 6.18 | <0.01 |
| Wang, 2022 | 4215 | 8370 | 4.86 | 3.75 | 6.3 | <0.01 |
| Kritsotakis 2011 | 4214 | 8457 | 4.72 | 3.65 | 6.11 | <0.01 |
| Wu, 2022 | 4242 | 8462 | 4.67 | 3.62 | 6.03 | <0.01 |
| liang, 2022 | 4254 | 8561 | 4.94 | 3.84 | 6.35 | <0.01 |
| Liu 2012 | 4285 | 8558 | 4.85 | 3.75 | 6.28 | <0.01 |
| Hussein, 2013 | 4207 | 8394 | 4.79 | 3.69 | 6.22 | <0.01 |
| Vardakas, 2015 | 4241 | 8590 | 4.96 | 3.86 | 6.36 | <0.01 |
| Zheng X 2017 | 4279 | 8591 | 4.7 | 3.65 | 6.06 | <0.01 |

# **Table S40 Sensitivity analysis for associations between Glycopeptides and CRKP infections in enrolled studies**

| Removed_Study | Sample_Size_Case | Sample_Size_Control | OR_Random | Lower_CI_Random | Upper_CI_Random | P_Value_Random |
| --- | --- | --- | --- | --- | --- | --- |
| None(n=25) | 2903 | 6074 | 3.4 | 2.65 | 4.35 | <0.01 |
| Falagas, 2007 | 2859 | 6030 | 3.36 | 2.6 | 4.33 | <0.01 |
| Wu, 2011 | 2864 | 5996 | 3.43 | 2.65 | 4.43 | <0.01 |
| Wang, 2018 | 2855 | 6026 | 3.4 | 2.64 | 4.38 | <0.01 |
| Zheng, 2018 | 2844 | 5844 | 3.25 | 2.54 | 4.15 | <0.01 |
| Liu, 2019 | 2883 | 6005 | 3.4 | 2.65 | 4.36 | <0.01 |
| Chang, 2019 | 2857 | 5835 | 3.34 | 2.58 | 4.32 | <0.01 |
| Zhang, 2021 | 2761 | 5932 | 3.39 | 2.61 | 4.39 | <0.01 |
| Zhang, 2021 | 2806 | 6033 | 3.51 | 2.74 | 4.5 | <0.01 |
| Zuo, 2020 | 2829 | 6000 | 3.46 | 2.68 | 4.47 | <0.01 |
| Chen, 2022 | 2691 | 5580 | 3.61 | 2.86 | 4.55 | <0.01 |
| Lou, 2022 | 2481 | 5126 | 3.3 | 2.56 | 4.26 | <0.01 |
| Çölkesen, 2023 | 2771 | 5924 | 3.54 | 2.76 | 4.54 | <0.01 |
| Huang, 2023 | 2409 | 4717 | 3.49 | 2.69 | 4.52 | <0.01 |
| Wang, 2023 | 2729 | 5855 | 3.48 | 2.69 | 4.5 | <0.01 |
| Cheng, 2024 | 2853 | 5990 | 3.35 | 2.6 | 4.33 | <0.01 |
| Hussein, 2009 | 2815 | 5701 | 3.42 | 2.64 | 4.45 | <0.01 |
| zheng, 2017 | 2852 | 6023 | 3.36 | 2.6 | 4.34 | <0.01 |
| Gupta, 2020 | 2818 | 6048 | 3.41 | 2.65 | 4.37 | <0.01 |
| Li, 2020 | 2739 | 5746 | 3.25 | 2.54 | 4.16 | <0.01 |
| Liu, 2022 | 2829 | 5701 | 3.27 | 2.55 | 4.2 | <0.01 |
| Dai, 2021 | 2812 | 5983 | 3.45 | 2.67 | 4.44 | <0.01 |
| Wang, 2022 | 2808 | 5836 | 3.47 | 2.68 | 4.49 | <0.01 |
| Wu, 2022 | 2835 | 5928 | 3.27 | 2.56 | 4.19 | <0.01 |
| Hussein, 2013 | 2800 | 5860 | 3.4 | 2.62 | 4.41 | <0.01 |
| Zheng, 2017 | 2872 | 6057 | 3.37 | 2.62 | 4.33 | <0.01 |

# **Table S41 Sensitivity analysis for associations between Cephalosporins and CRKP infections in enrolled studies**

| Removed_Study | Sample_Size_Case | Sample_Size_Control | OR_Random | Lower_CI_Random | Upper_CI_Random | P_Value_Random |
| --- | --- | --- | --- | --- | --- | --- |
| None(n=36) | 3603 | 7644 | 1.83 | 1.38 | 2.43 | <0.01 |
| Falagas, 2007 | 3559 | 7600 | 1.82 | 1.36 | 2.42 | <0.01 |
| Patel, 2008 | 3504 | 7545 | 1.79 | 1.35 | 2.38 | <0.01 |
| Wu, 2011 | 3564 | 7566 | 1.84 | 1.37 | 2.45 | <0.01 |
| Simkins, 2014 | 3590 | 7605 | 1.79 | 1.35 | 2.37 | <0.01 |
| Candevir, 2015 | 3556 | 7593 | 1.81 | 1.36 | 2.41 | <0.01 |
| Hu, 2016 | 3538 | 7579 | 1.85 | 1.39 | 2.48 | <0.01 |
| Wang, 2018 | 3555 | 7596 | 1.8 | 1.35 | 2.4 | <0.01 |
| Xiao, 2018 | 3468 | 7351 | 1.8 | 1.35 | 2.4 | <0.01 |
| Zheng, 2018 | 3544 | 7414 | 1.87 | 1.4 | 2.49 | <0.01 |
| Cienfuegos, 2019 | 3554 | 7355 | 1.76 | 1.34 | 2.32 | <0.01 |
| Liu, 2019 | 3583 | 7575 | 1.85 | 1.39 | 2.45 | <0.01 |
| Chang, 2019 | 3557 | 7405 | 1.9 | 1.44 | 2.51 | <0.01 |
| Xiao, 2020 | 3499 | 7377 | 1.8 | 1.35 | 2.39 | <0.01 |
| Zhang, 2021 | 3461 | 7502 | 1.86 | 1.39 | 2.48 | <0.01 |
| Zuo, 2020 | 3529 | 7570 | 1.88 | 1.41 | 2.5 | <0.01 |
| Chen, 2022 | 3391 | 7150 | 1.86 | 1.39 | 2.49 | <0.01 |
| Chen, 2022 | 3574 | 7421 | 1.8 | 1.35 | 2.39 | <0.01 |
| Lou, 2022 | 3181 | 6696 | 1.82 | 1.36 | 2.44 | <0.01 |
| Çölkesen, 2023 | 3471 | 7494 | 1.69 | 1.32 | 2.16 | <0.01 |
| Huang, 2023 | 3109 | 6287 | 1.91 | 1.44 | 2.52 | <0.01 |
| Wang, 2023 | 3429 | 7425 | 1.84 | 1.37 | 2.46 | <0.01 |
| Cheng, 2024 | 3553 | 7560 | 1.77 | 1.34 | 2.35 | <0.01 |
| Hussein, 2009 | 3515 | 7271 | 1.85 | 1.38 | 2.47 | <0.01 |
| zheng, 2017 | 3552 | 7593 | 1.85 | 1.38 | 2.47 | <0.01 |
| Gupta, 2020 | 3518 | 7618 | 1.86 | 1.4 | 2.48 | <0.01 |
| Li, 2020 | 3439 | 7316 | 1.86 | 1.39 | 2.49 | <0.01 |
| Liu, 2022 | 3529 | 7271 | 1.83 | 1.37 | 2.45 | <0.01 |
| Li, 2022 | 3537 | 7578 | 1.9 | 1.44 | 2.52 | <0.01 |
| Dai, 2021 | 3512 | 7553 | 1.89 | 1.42 | 2.51 | <0.01 |
| Wang, 2022 | 3508 | 7406 | 1.85 | 1.39 | 2.48 | <0.01 |
| Kritsotakis 2011 | 3507 | 7493 | 1.87 | 1.4 | 2.5 | <0.01 |
| Wu, 2022 | 3535 | 7498 | 1.87 | 1.41 | 2.5 | <0.01 |
| Liu 2012 | 3578 | 7594 | 1.78 | 1.34 | 2.35 | <0.01 |
| Hussein, 2013 | 3500 | 7430 | 1.8 | 1.35 | 2.4 | <0.01 |
| Vardakas, 2015 | 3534 | 7626 | 1.9 | 1.44 | 2.51 | <0.01 |
| Zheng, 2017 | 3572 | 7627 | 1.84 | 1.38 | 2.45 | <0.01 |

# **Table S42 Sensitivity analysis for associations between Nitroimidazoles and CRKP infections in enrolled studies**

| Removed_Study | Sample_Size_Case | Sample_Size_Control | OR_Random | Lower_CI_Random | Upper_CI_Random | P_Value_Random |
| --- | --- | --- | --- | --- | --- | --- |
| None(n=12) | 1720 | 3845 | 1.21 | 0.86 | 1.71 | 0.27 |
| Falagas, 2007 | 1676 | 3801 | 1.25 | 0.86 | 1.81 | 0.24 |
| Wu, 2011 | 1681 | 3767 | 1.27 | 0.88 | 1.83 | 0.21 |
| Wang, 2018 | 1672 | 3797 | 1.21 | 0.84 | 1.75 | 0.30 |
| Zheng, 2018 | 1661 | 3615 | 1.03 | 0.8 | 1.32 | 0.83 |
| Chen, 2022 | 1508 | 3351 | 1.17 | 0.8 | 1.71 | 0.41 |
| Lou, 2022 | 1298 | 2897 | 1.30 | 0.9 | 1.88 | 0.17 |
| Huang, 2023 | 1226 | 2488 | 1.30 | 0.9 | 1.88 | 0.17 |
| zheng, 2017 | 1669 | 3794 | 1.13 | 0.81 | 1.59 | 0.47 |
| Li, 2020 | 1556 | 3517 | 1.21 | 0.83 | 1.77 | 0.32 |
| Li, 2022 | 1654 | 3779 | 1.23 | 0.87 | 1.74 | 0.24 |
| Kritsotakis 2011 | 1624 | 3694 | 1.21 | 0.82 | 1.80 | 0.33 |
| Liu 2012 | 1695 | 3795 | 1.24 | 0.86 | 1.78 | 0.25 |

# Table S43 Pooled results of risk factors for CRKP infection after excluding low-quality studies

| Risk factors | n | OR/MD (95%CI) | Effect model | *I^2^* |
| --- | --- | --- | --- | --- |
| Demo |  |  |  |  |
| Sex (Male) | 17 | 1.36[1.12;1.70] | Random | 54.0% |
| Age | 12 | 0.69[-0.38;1.75] | Fix | 26.5% |
| Malignant tumors | 14 | 0.73 [0.53; 1.02] | Random | 56.6%， |
| liver disease | 7 | 1.17 [0.79; 1.73] | Fix | 23.6%， |
| Hypertension | 7 | 1.75 [1.05;2.94] | Random | 60.0%， |
| Respiratory system disease | 3 | 3.44 [2.32; 5.12] | Fix | 10.9% |
| Mental illness | 6 | 1.27 [0.89; 1.82] | Fix | 0.0% |
| Chronic lung disease | 11 | 1.22 [0.75; 1.97] | Random | 67.3% |
| Kidney disease | 15 | 1.47 [1.20;1.80] | Random | 41.1% |
| Diabetes mellitus | 16 | 0.99 [0.73; 1.35] | Random | 68.7% |
| Cardiovascular disease | 12 | 1.20 [0.87; 1.64] | Random | 48.9%, |
| Hematological malignancy | 6 | 0.76[0.26;2.23] | Random | 93.6% |
| Invasive operation |  |  |  |  |
| Nasogastric catheter | 6 | 1.96[0.89;4.35] | random | 93.7% |
| Parenteral nutrition | 6 | 2.04[1.08;3.88] | random | 85.4% |
| Mechanical ventilation | 16 | 3.63[2.31;5.68] | random | 85.9% |
| Indwelling urinary catheter | 11 | 3.03[1.83;5.03] | random | 90.0% |
| Endoscopy | 4 | 4.89[1.20;19.85] | random | 90.0% |
| Tracheal cannula | 4 | 5.11[1.47;17.74] | random | 96.5% |
| Tracheostomy | 10 | 2.96[1.66;5.31] | random | 77.7% |
| Dialysis | 5 | 3.49[2.20;5.54] | Fix | 0% |
| Surgical drainage | 3 | 1.97 [1.24; 3.14] | Fix | 0% |
| Peripheral Catheter | 3 | 3.12 [1.55; 6.29] | random | 75.5%， |
| Central venous catheter | 13 | 3.75 [2.19;6.42] | random | 88.6% |
| Hospital Environment |  |  |  |  |
| ICU admission | 15 | 4.22[2.61;7.11] | random | 88.2% |
| Hospital Stay Before Infection | 3 | 16.40[2.26;30.55] | random | 75.3% |
| Prior hospitalization | 7 | 2.01[1.21;3.34] | random | 55.8% |
| Antibiotic |  |  |  |  |
| B-lactamase inhibitor | 6 | 2.96 [1.87; 4.71] | random | 83.2%， |
| Aminoglycoside | 10 | 2.43 [1.76; 3.36] | Fix | 30.5% |
| Macrolides | 3 | 2.26 [0.44;11.71] | random | 83.3% |
| Polymyxin | 6 | 5.65 [2.20; 14.48] | random | 60.3% |
| Oxazolidones | 3 | 4.00 [0.94; 16.99] | random | 82.9% |
| Fluoroquinolone | 14 | 2.05 [1.59; 2.66] | random | 56.3% |
| Glycyrylcyclines | 8 | 4.53 [2.10; 9.76] | random | 74.3% |
| Antifungal agents | 4 | 3.87 [2.55; 5.88] | Fix | 0.0% |
| Penicillins | 8 | 1.57 [0.90; 2.74] | random | 74.3% |
| Carbapenems | 17 | 4.53 [3.07; 6.68] | random | 87.1%， |
| Glycopeptides | 12 | 4.01 [2.71; 5.95] | random | 77.6% |
| Cephalosporins | 14 | 1.47 [1.07; 2.02] | random | 68.9% |
| Nitroimidazoles | 5 | 1.41 [0.98; 2.01] | Fix | 0.0% |

# Table S44 Pooled Odds Ratios and Heterogeneity (I²) of Risk Factors for CRKP infections by Time Period and Study Design

| **risk factors** | **overall** | | **Year** | | | | **Study design** | | | |
| --- | --- | --- | --- | --- | --- | --- | --- | --- | --- | --- |
|  |  |  | **Pre-2015** | | **After-2015** | | **case-control study** | | **cohort study** | |
|  | **OR 95%CI** | ***I^2^*** | **OR 95%CI** | ***I^2^*** | **OR 95%CI** | ***I^2^*** | **OR 95%CI** | ***I^2^*** | **OR 95%CI** | ***I^2^*** |
| Chronic lung disease | 1.28 [ 0.93; 1.75] | 70.90% | 1.54 [0.48; 4.97] | 52.1% | 1.27 [0.91; 1.77] | 52.9% | 1.42 [0.94; 2.15] | 59.3% | 1.12 [0.85; 1.48] | 19.6% |
| Hematological malignancy | 0.85 [ 0.38; 1.94] | 90.20% | 1.73 [1.00; 3.00] | 62.4% | 0.77 [0.29; 2.08] | 44.5% | 0.87 [0.26; 2.87] | 24.8% | 0.82 [0.23; 2.90] | 7.4% |
| Endoscopy | 4.08 [1.40; 11.9] | 86.70% | 4.50 [2.18; 9.28] | 68.5% | 3.35 [1.08; 17.52] | 35.0% | 6.39 [1.80; 22.7] | 67.4% | 2.60 [1.29; 23.5] | 46.9% |
| Tracheal cannula | 3.72 [2.10; 6.60] | 91.50% | 2.02 [1.12; 3.66] | 64.1% | 4.27 [2.18; 8.36] | 33.6% | 4.13 [2.29; 7.44] | 49.3% | 3.22 [0.92; 11.3] | 6.4% |
| Mechanical ventilation | 3.61 [2.72; 4.78] | 84.70% | 2.53 [1.69;3.79] | 55.1% | 4.03 [2.81; 5.79] | 52.1% | 3.71 [1.70; 7.66] | 32.1% | 3.39 [2.54; 4.52] | 22.5% |
| Central venous catheter | 3.39 [2.40; 4.79] | 86.80% | 2.86 [1.40; 5.85] | 42.9% | 3.42 [2.32; 5.05] | 30.2% | 3.34 [2.30; 4.85] | 74.2% | 2.91 [1.17; 7.24] | 62.4% |
| Tracheostomy | 2.96 [2.14; 4.09] | 89.50% | 3.61 [1.07; 6.34] | 71.4% | 2.80 [1.88;4.19] | 52.7% | 3.75 [1.51; 9.28] | 45.0% | 2.57 [1.67; 3.95] | 42.6% |
| Indwelling urinary catheter | 2.84 [1.91; 4.21] | 80.10% | 3.30 [2.30; 4.73] | 59.6% | 1.95[1.04; 3.68] | 34.4% | 3.41 [0.99;11.74] | 49.5% | 2.85 [2.07; 3.92] | 28.9% |
| Nasogastric catheter | 2.41 [1.54; 3.77] | 89.10% | 1.69 [1.14; 6.77] | 52.9% | 2.57 [1.58; 4.19] | 42.9% | 2.48 [1.53; 4.04] | 58.1% | 2.32 [0.72; 7.51] | 48.4% |
| Parenteral nutrition | 1.78 [1.18; 2.68] | 82.40% | 1.29 [1.06; 2.19] | 47.3% | 1.99 [1.26; 3.16] | 40.2% | 1.98 [1.28; 3.06] | 76.9% | 1.19 [0.86; 1.64] | 32.9% |
| ICU admission | 4.27 [3.22; 5.66] | 85.30% | 5.24 [3.18;8.64] | 52.9% | 4.02 [2.86; 5.63] | 18.8% | 6.67 [3.45; 12.9] | 43.3% | 3.72 [2.77; 5.00] | 6.1% |
| Carbapenems | 4.79 [3.72; 6.18] | 85.20% | 4.33 [2.77; 6.76] | 63.7% | 4.93 [3.67; 6.62] | 49.6% | 5.28 [4.02; 6.92] | 72.0% | 3.49 [2.04;5.95] | 10.3% |
| Glycopeptides | 3.40 [2.65; 4.35] | 72.60% | 3.20 [2.39; 4.29] | 56.4% | 3.42 [2.54; 4.59] | 18.3% | 3.50[2.78; 4.42] | 53.1% | 2.83 [1.05; 7.61] | 48.2% |
| Fluoroquinolone | 2.37 [1.81; 3.11] | 83.90% | 2.30[1.65;3.19] | 76.7% | 2.52 [1.80;3.52] | 16.2% | 2.42 [1.79; 3.27] | 48.9% | 1.97 [1.21; 3.20] | 26.4% |
| Polymyxin | 4.25 [2.02, 8.95] | 62.90% | 4.36 [1.56;12.2] | 73.2% | 4.30 [1.21;15.4] | 50.0% | 5.17 [2.38; 11.2] | 60.8% | 2.15 [1.29; 16.1] | 49.7% |
| β-lactamase inhibitor | 2.24 [1.80; 2.79] | 71.10% | 2.94 [2.07; 4.17] | 44.2% | 2.10 [1.65; 2.67] | 38.6% | 2.44 [1.47; 4.05] | 50.7% | 2.14 [1.68; 2.72] | 24.1% |
| Cephalosporins | 1.83 [1.38; 2.43] | 85.10% | 1.77 [1.25;2.52] | 70.7% | 2.12 [1.27; 3.55] | 9.3% | 1.99 [1.45; 2.75] | 40.5% | 1.40 [1.07;2.81] | 36.6% |
| Penicillins | 1.54 [1.06; 2.25] | 70.30% | 1.70[1.05;2.13] | 60.8% | 1.08 [1.02;1.53] | 32.3% | 1.53 [1.01; 2.30] | 47.2% | 1.63 [1.38;3.17] | 25.9% |

# Table S45 Pooled Odds Ratios and Heterogeneity (I²) of Risk Factors for CRKP infections by region

| **Risk factors** | **Overall** | | **Eastern** | **Western** |
| --- | --- | --- | --- | --- |
|  | **OR 95%CI** | ***I^2^*** | **OR 95%CI** | **OR 95%CI** |
| Chronic lung disease | 1.28 [ 0.93; 1.75] | 70.90% | 1.38 [0.93; 2.05] | 1.00 [0.63; 1.59] |
| Hematological malignancy | 0.85 [ 0.38; 1.94] | 90.20% | 0.78 [0.63; 0.97] | 1.23 [0.57; 2.66] |
| Endoscopy | 4.08 [1.40; 11.9] | 86.70% | 4.35 [1.08; 17.5] | 4.50 [2.18; 9.28] |
| Mechanical ventilation | 3.61 [2.72; 4.78] | 84.70% | 4.22 [2.95; 6.04] | 2.34 [1.60; 3.42] |
| Central venous catheter | 3.39 [2.40; 4.79] | 86.80% | 3.53 [2.33; 5.36] | 2.77 [1.48; 5.17] |
| Tracheostomy | 2.96 [2.14; 4.09] | 89.50% | 2.49 [1.03; 6.03] | 2.91 [1.99; 4.26] |
| Indwelling urinary catheter | 2.84 [1.91; 4.21] | 80.10% | 3.54 [2.41; 5.18] | 1.88 [1.15; 3.10] |
| Nasogastric catheter | 2.41 [1.54; 3.77] | 89.10% | 2.57 [1.58; 4.19] | 1.69 [0.42; 6.77] |
| Parenteral nutrition | 1.78 [1.18; 2.68] | 82.40% | 1.97 [1.14; 3.41] | 1.60 [1.03; 2.50] |
| ICU admission | 4.27 [3.22; 5.66] | 85.30% | 4.59 [3.30; 6.38] | 3.51 [2.00; 6.15] |
| Carbapenems | 4.79 [3.72; 6.18] | 85.20% | 4.48 [3.37; 5.97] | 5.74 [3.50; 9.41] |
| Glycopeptides | 3.40 [2.65; 4.35] | 72.60% | 3.54 [2.66; 4.73] | 2.73 [1.89; 3.94] |
| Fluoroquinolone | 2.37 [1.81; 3.11] | 83.90% | 1.89 [1.54; 2.32] | 3.58 [1.61; 7.96] |
| Polymyxin | 4.25 [2.02, 8.95] | 62.90% | 6.10 [2.59; 14.4] | 3.26 [1.05; 10.1] |
| β-lactamase inhibitor | 2.24 [1.80; 2.79] | 71.10% | 2.20 [1.75; 2.77] | 2.37 [1.45; 3.87] |
| Cephalosporins | 1.83 [1.38; 2.43] | 85.10% | 1.55 [1.18; 2.03] | 2.68 [1.38; 5.20] |
| Penicillin | 1.54 [1.06; 2.25] | 70.30% | 1.14 [0.59; 2.19] | 1.91 [1.28; 2.83] |
